# Identification, Expression of AaSQSTM1 in *Aedes albopictus* and Its Autophagic Function Analysis

**DOI:** 10.3390/insects16100994

**Published:** 2025-09-24

**Authors:** Haodong Xu, Yijia Huang, Zihan Liang, Xiao Feng, Nan Wang, Haojie Wang, Sheng Gao, Hongbo Li, Wenquan Liu, Shaohui Liang

**Affiliations:** 1 Department of Parasitology, School of Basic Medical Sciences, Wenzhou Medical University, Wenzhou 325035, China; ssee6141@outlook.com (H.X.); huangyjyj@163.com (Y.H.); 15271213193@163.com (Z.L.); 19861121136@163.com (X.F.); 15168572369@163.com (N.W.); w13857577929@163.com (H.W.); gs171706zh@163.com (S.G.); lhb2772909211@163.com (H.L.); 2 Department of Pathogenic Biology, School of Basic Medical Sciences, Soochow University, Suzhou 215123, China

**Keywords:** *Aedes albopictus*, autophagy, functional analysis, SQSTM1

## Abstract

Autophagy is a central pathway involved in maintaining cellular homeostasis during development, metabolism, and regeneration. The selective autophagy receptor p62/SQSTM1 is a multifunctional protein that plays a critical role in regulating autophagic activity by interacting with LC3/Atg8 proteins and ubiquitinated substrates. *Aedes albopictus* has rapidly spread worldwide and poses a serious threat to human health by transmitting dengue and other arboviral diseases. In the present study, we identified a putative p62/SQSTM1 homolog protein (AaSQSTM1) in *Ae. albopictus*, which contains conserved structural regions, including the Atg8 family interaction motif (AIM) and ubiquitin-associated (UBA) domain. The expression levels of AaSQSTM1 and AaAtg8 varied from the egg stage to the adult stage in *Ae. albopictus*. Quantitative analyses revealed that blood-feeding upregulated the expression levels of AaSQSTM1 and AaAtg8 in the ovaries of female adults. AaSQSTM1 was applied to monitor autophagic activity alone or with AaAtg8 by assaying endogenous protein levels and overexpressing fluorescent reporters in C6/36 cells. Our study revealed that AaSQSTM1 regulates the autophagic activity with AaAtg8, providing a basis for studying the autophagy process in mosquitoes.

## Simple Summary

*Aedes albopictus* (the Asian tiger mosquito) poses a serious threat to human health worldwide by transmitting dengue and other arboviral diseases. Autophagy is an evolutionarily conserved catabolic process in eukaryotic cells that regulates the metamorphosis, reproduction, and lifespan of insects. We identified a mammal autophagy receptor p62/SQSTM1 homolog in *Ae. albopictus*, named AaSQSTM1. Quantitative analysis showed distinct AaSQSTM1 expression levels across various developmental stages and female tissues. We also demonstrated that AaSQSTM1 could be used as a useful marker to estimate autophagy activity with the autophagy-related protein Atg8 in C6/36 cells. This study provides a basis for exploring the function of autophagy in *Ae. albopictus* mosquitoes.

## 1. Introduction

Autophagy, a highly conserved cellular degradation mechanism in eukaryotic cells, is responsible for breaking down impaired organelles and aggregated proteins via lysosomal pathways [1]. The induction of autophagy can be triggered by a range of signals, including nutrient starvation, drug treatment, and pathogen infection [2,3]. In terms of how cargo is delivered, autophagy can be categorized into three types: macroautophagy, microautophagy, and chaperone-mediated autophagy (CMA). Macroautophagy (usually referred to as autophagy) is the most common form of autophagy and plays essential roles in development, immunity, and cell death [4,5].

Autophagy can be either selective or nonselective [6]. Unlike starvation-induced nonselective autophagy indiscriminately sequesters random cytosolic regions, selective autophagy recognizes and binds to specific cargoes by utilizing autophagy receptors, subsequently delivers them to the autophagic machinery [1,6]. During selective autophagy, autophagy substrates are labeled with ubiquitin, and autophagy receptors bind with ubiquitin cargos and deliver them to the inner surface of the growing phagophore by interactions with the autophagy-related proteins LC3/Atg8 [6]. The phagophore elongates and subsequently encloses the ubiquitin cargo to form an autophagosome. Then the autophagosome fuses with the lysosome to form the autolysosome. After fusion, the enclosed cytoplasmic materials are degraded by lysosomal enzymes, and the degradation products are delivered back to the cytoplasm for recycling [6]. To date, several autophagy receptors, including p62/SQSTM1, NBR1, NDP52, OPTN, and TAX1BP1, have been reported in mammals [7,8].

p62/SQSTM1 (hereafter referred to as SQSTM1) is the first selective autophagy receptor identified in mammalian cells [9]. SQSTM1 interacts with various binding partners via highly conserved functional domains, including a Phox1 and Bem1p (PB1) domain, a ZZ-type zinc finger (ZZ) domain, a ubiquitin-associated (UBA) domain, and an LC3 interaction region (LIR), which is also known as the Atg8 family interaction motif (AIM) [10]. The PB1 domain is located at the N-terminus of SQSTM1 and is responsible for oligomerization, packaging of ubiquitinated cargos, and delivery of SQSTM1-coated cargos to the autophagy pathway [11]. The ZZ domain binds to receptor-interacting protein kinase 1 (RIP1), a key regulator of inflammatory signaling [12]. The UBA domain is in the C-terminus of SQSTM1, which interacts directly with ubiquitinated cargoes and targets them for degradation by autophagy and/or the proteasome pathway [12,13]. In addition, SQSTM1 contains an LIR/AIM domain, which consists of an acidic cluster of “DDD” residues and hydrophobic “WxxL” residues (x represents a negatively charged amino acid) [14]. SQSTM1 directly binds with LC3/Atg8 to deliver ubiquitinated cargoes for degradation in autolysosomes [15]. The activation and inhibition of the autophagy process lead to the degradation and accumulation of SQSTM1 individually [10]. Therefore, SQSTM1 can be used as a marker for monitoring autophagic activity in mammalian cells under the treatment with Hank’s Balanced Salt Solution (HBSS) and several chemical drugs such as rapamycin, BafA1 and MG132 [16,17].

*Aedes albopictus* (Skuse, 1895), also known as the Asian tiger mosquito, is a major vector for several arboviruses, including dengue virus (DENV), chikungunya virus (CHIV), and Zika virus (ZIKV) [18]. *Aedes albopictus* (*Ae. albopictus*) is currently among the top 100 invasive species worldwide and has spread from the Western Pacific and Southeast Asia to Europe, Africa, and Central and South America over the last two decades [19]. Its rapid spread and increased resistance to pesticides underscore concern regarding the potential global expansion of arboviral diseases [18]. Autophagy is not only involved in insect development, regeneration body remodeling, and metabolism [7,20], but also regulates the interaction between *Aedes* mosquitoes and arbovirus [21,22]. *Drosophila* Ref(2)P (refractory to sigma P) is the ortholog of SQSTM1 and is not only required for the control of sigma virus multiplication but also plays an important role in regulating proteostasis and metabolism [23,24,25]. Previous studies have suggested that the upregulation of Ref(2)P prolongs health and longevity in several invertebrates, such as *Drosophila melanogaster* (*D. melanogaster*) and *Caenorhabditis elegans* (*C. elegans*) [24,26]. Like SQSTM1, Ref(2)P contains a putative AIM domain, and inhibition of autophagy leads to the accumulation of Ref(2)P protein, indicating that Ref(2)P may serve as a marker to monitor autophagic activity in *Drosophila* [17,24]. However, until now, SQSTM1, as an autophagy receptor, has not been well characterized in mosquitoes.

In this study, we identified the *Ae. albopictus* ortholog of SQSTM1 (AaSQSTM1) and characterized its sequence features and functional domain. We also investigated the expression characteristics of AaSQSTM1 in different developmental stages and female tissues. A functional assay of the ability of AaSQSTM1 and AaAtg8 to monitor autophagy activity was carried out in C6/36 cells in response to starvation and rapamycin induction. The results of this study revealed that AaSQSTM1 can be a useful marker for detecting autophagy activity in *Ae. albopictus*.

## 2. Materials and Methods

### 2.1. Sequence Alignments and Phylogenetic Tree Analysis

To identify SQSTM1 homologs in *Ae. albopictus*, the amino acid sequence of Ref(2)P from *D. melanogaster* (GenBank ID: AAA98838.1) was used as a query in BLAST (https://blast.ncbi.nlm.nih.gov/Blast.cgi, accessed on 6 June 2024) searches. Comprehensive searches were conducted to generate the putative AaSQSTM1 sequences using the reference protein database of *Ae. albopictus* (NCBI taxid: 7160). Similarly, the SQSTM1 homolog sequences from other mosquito species: *Aedes aegypti* (*Ae. aegypti*) *Anopheles gambiae* (*An. gambiae*), *Culex quinquefasciatus* (*Cx. quinquefasciatus*), and *Culex pipiens pallens* (*Cx. pipiens pallens*), as well as the representative model organisms: *Bombyx mori* (*B. mori*), *D. melanogaster*, *C. elegans*, *Homo sapiens* (*H. sapiens*) and *Mus musculus* (*M. musculus*) were blasted on the NCBI website using Clustal-X. The amino acid alignments of SQSTM1 were performed in ESPript 3.0 (https://espript.ibcp.fr/ESPript/cgi-bin/ESPript.cgi, accessed on 6 June 2024). The phylogenetic analysis of SQSTM1 was performed using the neighbor-joining (NJ) method in the MEGA 11 phylogenetic software package [27]. The accession numbers of the SQSTM1 sequences in NCBI are shown in the phylogenetic tree.

### 2.2. Structural Modeling of Mosquito SQSTM1

The conserved amino acids in the motif and functional domains of SQSTM1 were predicted using the MEME website (Introduction-MEME Suite) and identified by comparison with previously published sequences of SQSTM1 from model organisms. The domain architectures were plotted on the IBS 2.0 website (https://www.ibs.renlab.org, accessed on 6 June 2024). The PDB structure files of the *Ae*. *albopictus* AaSQSTM1 and the autophagy-related protein AaAtg8 were obtained from the PHYRE2 website (https://www.sbg.bio.ic.ac.uk/phyre2, accessed on 6 June 2024). The interaction model of these two proteins was simulated and predicted using the ClusPro 2.0 website (https://cluspro.org/help.php, accessed on 6 June 2024), and the model with the highest score and confidence in ClusPro 2.0 was visually analyzed in PyMOL version 2.5.0 [27].

### 2.3. Cell Culture and Mosquito Rearing, Blood Feeding, and Tissue Dissection

*Aedes albopictus* C6/36 cells (ATCC, CRL-1660) were maintained in Leibovitz L-15 medium (Gibco) supplemented with 10% fetal bovine serum (FBS) and antibiotics. C6/36 cells were cultured at 28 °C in a 5% CO_2_ atmosphere. Mosquito *Ae. albopictus* (Foshan strain) were reared at 28 °C and 80% humidity with a 12:12 h light: dark cycle. Larvae and pupae were grown in cups of distilled water and fed with ground yeast. Upon eclosion, adult mosquitoes were maintained with 10% sucrose solution. Mosquito samples from egg, early larva (L1 to L2 stages), late larva (L3 to L4 stages), pupa, as well as female and male adults were collected to examine the temporal expression of the AaSQSTM1 and AaAtg8 genes. Blood feeding of female adults were performed as described previously [28]. In brief, female adults were starved overnight before blood feeding. Artificial blood was purchased from JUSHI BioTech (Changyuan, China). Mosquitoes were allowed to feed on blood meals for 2 h and used for subsequent tissue dissections according to previous methods [29]. In brief, mosquitoes were sterile in 75% ethanol, and washed twice with sterile PBS. The cleaned mosquito was place onto a drop of sterile PBS on the microscope slide and dissected under the stereomicroscope (CEWEI PXS6555Pro, Shanghai, China). Firstly, hold the mosquito’s thorax with tweezers and gently pull on the mosquito’s head to separate it from the thorax. Then, cut at the thorax-abdomen junction to collect the thorax. Finally, the abdomen is open with sterilized forceps and/or needles, and the midgut, ovary and fat body were separately collected into 1.5 mL RNase-free tubes with 100 µL of sterile 1× PBS buffer. Approximately 20 mosquitoes were processed per treatment group. The tissues were homogenized and centrifuged at 12,000× *g* for 5 min at 4 °C, and the supernatants were transferred to new tubes for RNA and protein extraction.

### 2.4. Starvation Induction, Chemical Treatments and Antibodies

For starvation induction, C6/36 cells were maintained in Hank’s Balanced Salt Solution (HBSS; Gibco, Waltham, MA, USA) for 15 min, 30 min, 1 h, or 2 h [30]. When chemical treatment was performed, the C6/36 cells were treated with 5 µM rapamycin (MedChemExpress, Shanghai, China), 100 nM BafA1 (Sigma-Aldrich, St. Louis, MO, USA), or 20 µM MG132 (Sigma-Aldrich, St. Louis, MO, USA) for different durations as indicated [31]. The polyclonal anti-p62/SQSTM1 antibody (GeneTex, GTX100685, Irvine, CA, USA) has been applied for the detection of p62 in mosquito [32]. The anti-LC3/Atg8 antibody (MBL, PM037) was purchased form MBL life science (Woburn, MA, USA) and the anti-β-actin antibody (CST, #4967) and HRP-conjugated anti-rabbit IgG antibody (CST, #7074) were purchased from Cell Signaling Technology (Danvers, MA, USA).

### 2.5. RNA Extraction and RT-qPCR

Total RNA was extracted using the TRIzol reagent (Invitrogen, Waltham, MA, USA) according to the manufacturer’s protocols. After the treatment with RNase-free DNase, the isolated RNA was used as the template for reverse transcription–quantitative PCR (RT-qPCR) using ChamQ SYBR qPCR Master Mix (Vazyme Biotech, Nanjing, China) according to the manufacturer’s instructions in a Real-Time PCR System (Applied Biosystems, CA, USA) with the following program: 95 °C for 10 min and cycling at 95 °C for 15 s and 60 °C for 60 s for 40 cycles. The ribosomal S7 (*rps*7) gene was used as the reference gene. The relative mRNA transcription levels at different developmental stages and female tissues were quantified using the 2^−ΔΔCt^ method and expressed as a fold difference. The primers for RT-qPCR were listed in Table S1.

### 2.6. Protein Sample Preparation and Western Blot Analysis

The samples from mosquito cells and tissue homogenates were lysed in 150 μL of RIPA lysis buffer containing phosphatase and protease inhibitors. The supernatants were collected after centrifugation at 12,000× *g* for 10 min. The protein concentrations were detected using a BCA protein assay kit (Beyotime, Shanghai, China). Total protein samples were separated on 12% SDS-PAGE (Bio-Rad, Hercules, CA, USA) polyacrylamide gels and transferred to polyvinyl difluoride (PVDF) membranes (Thermo Scientific, Waltham, MA, USA). After being blocked with 5% milk in Tris-buffered Tween-20 (TBS-T), the PVDF membranes were labeled with primary antibodies: anti-SQSTM1, anti-Atg8 and anti-β-actin, which were separately used for detecting AaSQSTM1 (1:1000), AaAtg8 (1:2000) and β-actin (1:2000) overnight at 4 °C. A secondary antibody, a goat anti-rabbit mAb conjugated to horseradish peroxidase (HRP), was applied at a 1:5000 dilution and incubated with each membrane for 1 h at room temperature. The band signals were detected with Supersignal West Pico Chemiluminescent Reagent (Pierce, Appleton, WI, USA) for 5 min and the protein levels was quantified by densitometric analysis using ImageJ version 1.8.0. The background was subtracted using a rolling ball radius of 50 pixels. The integrated density of each target band was measured and normalized to the corresponding loading control band intensity. Relative band intensity was calculated as the ratio of normalized target protein (AaSQSTM1 and AaAtg8-II) intensity to the control group (β-actin and AaAtg8-I) with results presented as mean ± standard deviation from three independent experiments.

### 2.7. Plasmids Construction and Immunofluorescence Assay

The expression vector pIB-V5 plasmid containing OpIE2 promoter (Invitrogen) was applied for the recombinant plasmid construction. The OpIE2 promoter is from the baculovirus *Orgyia pseudotsugata* multicapsid nuclear polyhedrosis virus (OpMNPV), which can provide relatively high levels of constitutive expression in insect cells [33]. The gene fragments coding fluorescent proteins (mCherry and EGFP) were fused with the target genes (AaSQSTM1 and AaAtg8) by using GS linker (Gly-Gly-Gly-Gly-Ser), individually. The cDNAs of mCherry-AaSQSTM1, EGFP-AaAtg8, and mCherry-EGFP-AaSQSTM1 were synthetized by GenScript (Nanjing, China), and, respectively, inserted into the multiple cloning site (*Hind* III/*Xba* I) of pIB-V5 plasmid under the control of OpIE2 promoter (Figure S2). The recombinant plasmids were confirmed by sequencing.

C6/36 cells (5 × 10^5^ cells/mL) were separately plated on coverslips in 24-well culture plates overnight. The medium was then replaced with Opti-MEM for serum-free culture. The cells were co-transfected with 1 μg of pIB-EGFP-AaAtg8 and pIB-mCherry-AaSQSTM1 plasmids or with pIB-mCherry-EGFP-AaSQSTM1 using TransIT DNA Transfection Reagent (Mirus, Merck KGaA, Darmstadt, Germany). After treatment, the cells were incubated for 48 h and fixed with 4% (vol/vol) formaldehyde for 15 min. The slides were washed with PBS buffer containing 0.1% Triton X-100 (Sigma) for 10 min and blocked with 5% BSA in PBS-T for 1 h. The slides were incubated with DAPI (Solarbio, Beijing, China) before being mounted in Prolong Antifade Diamond Mountant (Life Technologies, Carlsbad, CA, USA) and analyzed by inverted confocal microscopy using a Leica DMI8 (Leica Microsystems, Wetzlar, Germany). Images of each single cell were cropped randomly, and Pearson’s colocalization coefficient were analyzed by using ImageJ color 2. At least 40 cells per group were included in each experiment.

### 2.8. Data Analysis

Data processing and analysis were performed using GraphPad Prism 10 software (GraphPad Software Inc., San Diego, CA, USA). Statistical analyses were performed using one-way ANOVA with a multiple comparison *t*-test according to the previous studies [34]. The values of gene expression were presented as the mean standard deviations (±SDs). The number of fluorescent puncta were presented as the standard error of mean (SEM). At least three independent experiments were performed for each assay (* *p* < 0.05, ** *p* < 0.01, *** *p* < 0.001 and **** *p* < 0.0001).

## 3. Results

### 3.1. Identification and Sequence Characterization of AaSQSTM1

**Figure 1:**
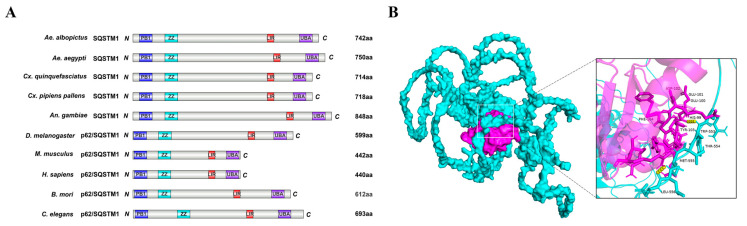
Domain architecture of SQSTM1 (**A**) and the interaction model of AaSQSTM1 and AaAtg8 (**B**). Domain architecture of SQSTM1 from *Ae. albopictus*, *Ae. aegypti*, *An. gambiae*, *Cx. quinquefasciatus, Cx. pipiens pallens*, *B. mori*, *D. melanogaster*, *C. elegans*, *H. sapiens* and *M. musculus*. The blue bar represents the Phox1 and Bem1p (PB1) domain, the cyan bar represents the ZZ-type zinc finger (ZZ) domain, the red bar represents the LC3 interaction region (LIR) domain, and the purple bar represents the ubiquitin-associated (UBA) domain. The amino acid numbers are indicated after the corresponding SQSTM1. The interaction model of AaSQSTM1 and AaAtg8 was predicted using the ClusPro website (https://cluspro.org/help.php, accessed on 6 June 2024), and green represents AaAtg8 and pink represents AaSQSTM1. The model with the highest score and confidence in ClusPro was visually analyzed in PyMOL version 2.5.0.

By performing a BLAST search on the NCBI website, we identified a putative SQSTM1 homolog (AaSQSTM1) among the reference proteins of *Ae. albopictus*. The open reading frame (ORF) of AaSQSTM1 comprises 2226 nucleotides encoding an 81.62 kDa protein with 742 amino acids (aa) (Figure 1). Moreover, SQSTM1 homologs were also identified in *Ae. aegypti*, *Cx. quinquefasciatus*, *Cx. pipiens pallens* and *An. gambiae*. The sequence lengths of the SQSTM1 proteins are 750 aa in *Ae. aegypti*, 714 aa in *Cx. quinquefasciatus*, 718 aa in *Cx. pipiens pallens* and 848 aa in *An. gambiae*. The SQSTM1 homologs from *D. melanogaster* and *B. mori* are 599 aa and 612 aa long, respectively, much shorter than those in mosquitoes. The SQSTM1 orthologs from *C. elegans*, *H. sapiens,* and *M. musculus* were included as reference sequences in the comparative alignment analysis. The percentages of amino acid conservation in SQSTM1 proteins between *Ae. albopictus* and *Ae. aegypti*, *Cx. quinquefasciatus*, *Cx. pipiens pallens* and *An. gambiae* and *D. melanogaster* were 89.1%, 67.4%, 67.1%, 44.2%, and 38.2%, respectively (Figure S1).

### 3.2. Phylogenetic Tree and Functional Domains of AaSQSTM1

The phylogenetic relationships of SQSTM1 derived from the orders Diptera, Lepidoptera, Nematoda and Vertebrata were analyzed based on amino acid sequence distances. The phylogenetic tree revealed that SQSTM1 proteins from *Ae. albopictus* and *Ae. aegypti* shared high homology and formed a single clade with the homologous SQSTM1 sequences from *Culex* and *Anopheles* mosquitoes (Figure S2). The phylogenetic analysis also revealed that SQSTM1 from mosquitoes was closer to the Ref(2)P protein from *D. melanogaster* than to homologs from *B. mori* and *C. elegans.* Bootstrap analysis indicated that the lineages of SQSTM1 were evolutionarily conserved across both invertebrate and vertebrate species, especially mosquito species.

Through functional domain analysis, we confirmed that AaSQSTM1 contains a PB1 domain (aa 27–92), a ZZ domain (aa 138–183), and a UBA domain (aa 696–742) (Figure 1A). The LIR (also referred to as AIM) domain of AaSQSTM1, located in the region from aa 524–556, shares a conserved acidic amino acid cluster (DDD) and four hydrophobic residues (WTML) (Figure S1). An interaction model of AaSQSTM1 and AaAtg8 was generated by adapting 3D prediction to confirm that the amino acid sequences of “WTML” in AaSQSTM1 and “HEEDYFL” in AaAtg8 are responded for the binding of these two proteins (Figure 1B).

### 3.3. Temporal Expression Patterns of AaSQSTM1 and AaAtg8 in Mosquito Development

**Figure 2:**
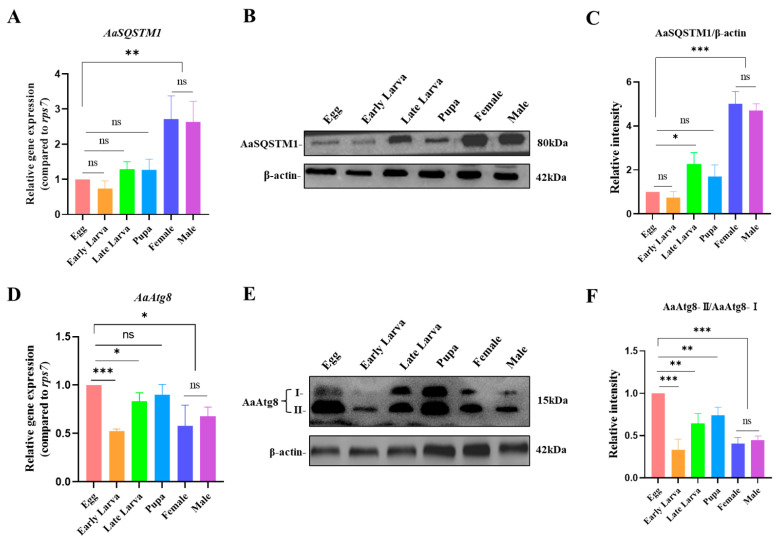
Expression patterns of AaSQSTM1 and AaAtg8 at different developmental stages. Mosquito samples from Egg, Early Larva (L1 to L2 stages), Late Larva (L3 to L4 stages), Pupa, Female and Male adults were homogenized on ice and subjected to RNA and protein extraction. (**A**,**D**) RNA samples were subjected to RT-qPCR to assess the mRNA transcription levels of AaSQSTM1 and AaAtg8. (**B**,**E**) The protein samples were subjected to Western blotting to assess the relative expression levels of AaSQSTM1 and AaAtg8. The RNA levels of the samples were normalized to those of the *rps*7 gene using the 2^−ΔΔCt^ method. (**C**,**F**) Protein levels of AaSQSTM1/β-actin and AaAtg8-II/AaAtg8-I between samples were normalized using band density analysis. The data represent three biological replicates with about 20 individuals in each treatment and are shown as the mean ± SEM; ns represents a nonsignificant difference, * *p* < 0.05, ** *p* < 0.01, *** *p* < 0.001.

Before studying the temporal expression patterns of AaSQSTM1 in *Ae. albopictus*, we firstly investigated whether the anti-p62/SQSTM1 antibody could be applied for the detection of AaSQSTM1 protein. We confirmed that the anti-p62/SQSTM1 antibody can specifically recognize the AaSQSTM1 protein in both cell and mosquito samples (Figure S3). Subsequently, we conducted a comprehensive assay of mRNA transcription and protein expression from the egg to adult stages. Relative expression analyses of mRNA transcription revealed that the mRNA levels of AaSQSTM1 from the egg to pupa stages were much lower than those in the adult stages (Figure 2A). We observed similar trends in the protein levels of AaSQSTM1 to its mRNA levels during the lifespan of *Ae. albopictus* (Figure 2B,C). The highest expression level of AaSQSTM1 was detected in the adult stages, with no significant difference between female and male adults.

Because AaSQSTM1 expression varies throughout the lifespan of *Ae. albopictus*, we aimed to examine whether AaAtg8 also exhibited different expression patterns. High levels of AaAtg8 mRNA transcription were detected in the egg and pupa stages and low levels of mRNA transcription were detected in the early larva (L1 to L2 stages) and adult stages (Figure 2D). The protein levels of AaAtg8 were consistent with its mRNA transcription levels in each developmental stage. We observed that the AaAtg8-II (AaAtg8-PE) protein was expressed at high levels in the egg and pupa stages and expressed at low levels in the larva and adult stages (Figure 2E,F). The expression levels of AaSQSTM1 and AaAtg8-II exhibited different tendencies from the egg to adult stages but were not significantly different between male and female adults. These findings suggest that AaSQSTM1 and AaAtg8 might be involved in regulating the metamorphosis of mosquitoes rather than sex determination.

### 3.4. Spatial Expression Patterns of AaSQSTM1 and AaAtg8 in Tissues from Female Adults

**Figure 3:**
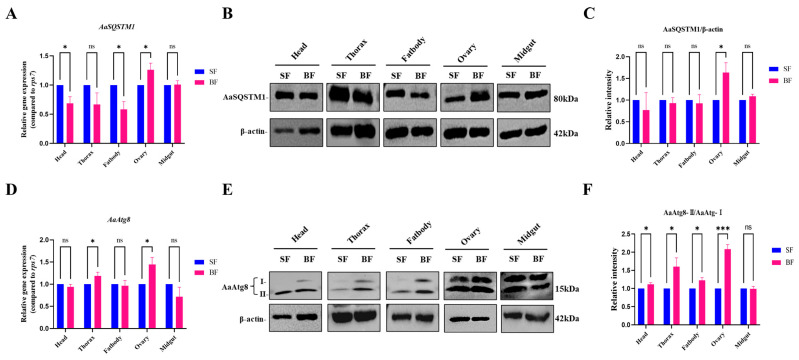
Relative expression levels of AaSQSTM1 and AaAtg8 in female mosquito tissues after sugar feeding (SF) and blood feeding (BF). After sugar-feeding and 24 h postblood-feeding, female adults were collected. Tissue samples, including the head, thorax, fat body, midgut, and ovary, from each treatment group were homogenized on ice and subjected to RNA and protein extraction. (**A**,**D**) RNA samples were subjected to RT-qPCR to assess the mRNA transcription levels of AaSQSTM1 and AaAtg8. The RNA levels of the samples were normalized to those of the *rps*7 gene using the 2^−ΔΔCt^ method. (**B**,**E**) The protein samples were subjected to Western blotting to assess the relative expression levels of AaSQSTM1 and AaAtg8. (**C**,**F**) The protein levels of AaSQSTM1/β-actin and AaAtg8-II/AaAtg8-I in the samples were normalized using band density analysis. The data represent three biological replicates with about 20 individuals in each and are shown as the mean ± SEM; ns represents a nonsignificant difference, * *p* < 0.05, *** *p* < 0.001.

Female mosquitoes need blood meals to develop their eggs. We collected female adults after sugar feeding (SF) and blood feeding (BF) individually to explore whether the expression patterns of AaSQSTM1 and AaAtg8 are affected by the diet switch from sugar to blood. The mRNA transcription and protein expression of AaSQSTM1 and AaAtg8 in the different tissues such as head, thorax, fatbody, ovary and midgut were investigated individually. The mRNA transcription levels of AaSQSTM1 were decreased in the head, thorax, and fat body but increased in the ovary after blood feeding (Figure 3A). Moreover, we observed that the protein levels of AaSQSTM1 were lower in the head, thorax, and fatbody in the blood-feeding (BF) groups than in the sugar-feeding (SF) groups. The protein expression of AaSQSTM1 was increased in the ovary after blood feeding (Figure 3B,C). Compared with those in sugar-feeding female adults, the mRNA transcription of AaAtg8 was increased in the thorax and ovary after blood meal consumption (Figure 3D). The protein levels of AaAtg8-II in the fatbody and ovary were also increased after blood feeding (Figure 3E,F). These expression patterns of the AaSQSTM1 and AaAtg8 genes suggest a possible role of autophagy in regulating mosquito ovary development after taking blood meals.

### 3.5. Endogenous AaSQSTM1 and AaAtg8 Expression Levels to Monitor Autophagy Activity

**Figure 4:**
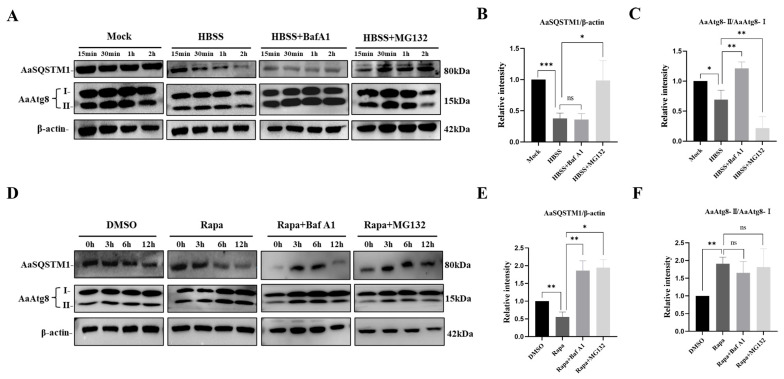
Western blot assays of AaSQSTM1 and AaAtg8 in autophagy-induced C6/36 cells. (**A**) C6/36 cells were treated with HBSS, HBSS plus BafA1, or HBSS plus MG132 for 15 min, 30 min, 1 h, or 2 h, respectively. C6/36 cells cultured with nutrient-rich base medium were used as the mock control. (**D**) C6/36 cells were treated with rapamycin (Rapa), Rapa plus BafA1, or Rapa plus MG132 for 0, 3, 6 and 12 h, respectively. C6/36 cells treated with DMSO were used as the control. The expression levels of AaSQSTM1 and AaAtg8 (AaAtg8-I and AaAtg8-II) were analyzed by Western blotting. The relative levels of AaSQSTM1/β-actin (**B**,**E**), AaAtg8-II/AaAtg8-I (**C**,**F**) between samples were quantified from three biological replicates by the detection of the mean signal intensity. The statistical significance was calculated by one-way ANOVA; ns represents a nonsignificant difference, * *p* < 0.05, ** *p* < 0.01, *** *p* < 0.001.

The detection of endogenous SQSTM1 expression levels has been applied to monitor autophagy activity in mammalian cells [16] and *Drosophila* [17]. However, reports on the use of SQSTM1-based assays for estimating autophagic activity in mosquito cells have not been reported. C6/36 cells were treated with starvation medium (HBSS) or rapamycin (Rapa) to investigate the AaSQSTM1 expression levels during autophagy induction. The lysosome inhibitor BafA1 and the proteasome inhibitor MG132 were separately added to C6/36 cells to inhibit autolysosome and proteasome degradation processes. Compared with those of the nutrient-rich base medium (Mock) treatment, the expression levels of both the AaSQSTM1 and AaAtg8-II proteins decreased after HBSS treatment for 2 h (Figure 4A,B). After BafA1 was added to HBSS, compared with the HBSS treatment group, AaSQSTM1 expression was still decreased, but AaAtg8-II expression recovered (Figure 4A,C). SQSTM1 not only regulates the autophagy process but also gets involved in the proteasome degradation pathway [13]. Therefore, MG132 was applied to inhibit the proteasome-mediated protein degradation pathway. As expected, AaSQSTM1 expression was significantly restored, but AaAtg8-II protein expression was dramatically decreased in the HBSS plus MG132 treatment group (Figure 4B,C). These findings indicated that AaSQSTM1 might be degraded by both the autolysosome and proteasome pathways under starvation induction.

Next, we used rapamycin, a widely used autophagy inducer, to assay the expression characteristics of AaSQSTM1 and AaAtg8. Compared with that in the DMSO treatment group, AaSQSTM1 protein expression decreased, whereas that of AaAtg8-II increased, after 6 h of rapamycin (Rapa) treatment (Figure 4D). Compared with those in the Rapa treatment alone, the protein expression levels of AaSQSTM1 in the Rapa plus BafA1 or Rapa plus MG132 treatments were significantly restored (Figure 4D,E). However, the endogenous expression of AaAtg8-II exhibited a decreasing trend without statistical significance in the Rapa treatments after adding either BafA1 or MG132 (Figure 4D,F). It suggested that AaSQSTM1 is more likely to be degraded by the autolysosome rather than the proteasome pathway under rapamycin induction.

### 3.6. Overexpressing Fluorescent AaSQSTM1 and the AaAtg8 Reporter to Monitor Autophagy Flux

**Figure 5:**
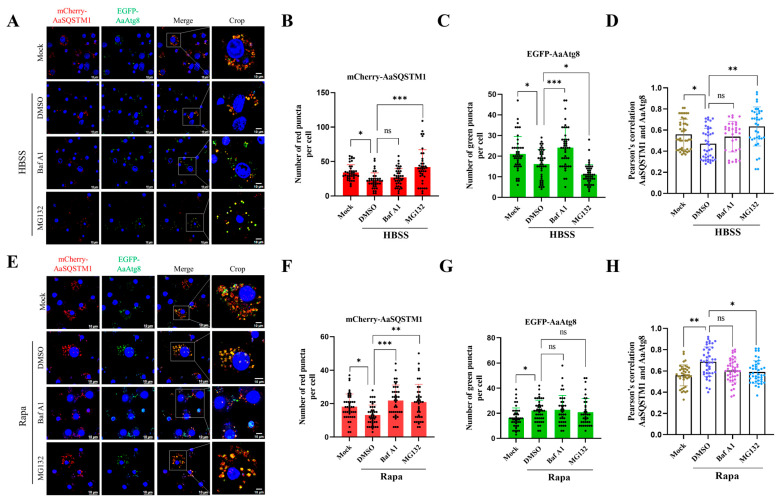
Fluorescence assays of AaSQSTM1 and AaAtg8 in autophagy-induced C6/36 cells. mCherry-AaSQSTM1 and EGFP-AaAtg8 plasmids were co-transfected into C6/36 cells for 48 h. (**A**) C6/36 cells were treated with HBSS, HBSS plus BafA1, or HBSS plus MG132 for 2 h; (**E**) C6/36 cells were treated with rapamycin, rapamycin plus BafA1, or rapamycin plus MG132 for 6 h. The cells were observed, and images of the cells were taken under a fluorescence microscope. DAPI was used to stain nuclear DNA, and the scale bar represents 10 μm. The average numbers of mCherry-AaSQSTM1 (**B**,**F**) and GFP-AaAtg8 (**C**,**G**) puncta in over 40 cells were determined using ImageJ. (**D**,**H**) The Pearson’s colocalization coefficient of mCherry-AaSQSTM1 and EGFP-AaAtg8 puncta. The statistical significance was calculated by one-way ANOVA; ns represents a nonsignificant difference, * *p* < 0.05, ** *p* < 0.01, *** *p* < 0.001.

Fluorescent p62/SQSTM1 and LC3/Atg8 assessments have been successfully applied for monitoring autophagy flux in many model organisms, including *Drosophila*, nematodes, zebrafish, and mice [14,16,17]. However, fluorescent SQSTM1 has not been investigated in mosquito cells. To determine whether fluorescent AaSQSTM1 and AaAtg8 could be used for monitoring autophagy activity in C6/36 cells, mCherry-AaSQSTM1 and EGFP-AaAtg8 plasmids were co-transfected into C6/36 cells, and autophagy activity was visualized by analyzing of fluorescent puncta via fluorescence microscopy. Compared with the number of fluorescent puncta (mCherry-AaSQSTM1 and EGFP-AaAtg8) in the mock control, in the HBSS treatment group, the numbers of red and green fluorescent puncta significantly decreased in C6/36 cells (red puncta: 34.0  ±  1.8 versus 22.8  ±  1.8; green puncta: 21.0  ±  1.3 versus 16.1  ±  1.1) (Figure 5A–C). We observed that the fluorescent puncta of mCherry-AaSQSTM1 and EGFP-AaAtg8 were individually modulated and significantly upregulated in the HBSS plus BafA1 treatment group compared with those in the HBSS treatment group (red puncta: 27.0 ±  2.0 versus 22.8  ±  1.8; green puncta: 24.1  ±  1.4 versus 16.1  ±  1.1). Furthermore, compared with HBSS treatment, HBSS plus MG132 treatment significantly changed the number of fluorescent puncta, increasing the number of mCherry-AaSQSTM1 fluorescent puncta (red puncta: 42.0  ±  3.9 versus 22.8  ±  1.8) and decreasing the number of EGFP-AaAtg8 fluorescent puncta (green puncta: 11.0  ±  0.7 versus 16.1  ±  1.1) (Figure 5A–C). Pearson’s coefficient showed that the colocalization of mCherry-AaSQSTM1 and EGFP-AaAtg8 puncta was decreased in the HBSS treatment but restored after adding either BafA1 or MG132 (Figure 5D).

Compared with those in the DMSO treatment, the number of mCherry-AaSQSTM1 fluorescent puncta decreased (red puncta: 13.0  ±  0.9 versus 19.4  ±  1.1), and the number of EGFP-AaAtg8 fluorescent puncta increased (green puncta: 22.1  ±  1.2 versus 16.3  ±  1.2) in the Rapa-treated C6/36 cells (Figure 5E–G). Compared with Rapa treatment alone, the addition of either BafA1 or MG132 significantly increased the number of red fluorescent puncta (red puncta: 21.5  ±  1.6 in Rapa plus BafA1, 20.3  ±  1.6 in Rapa plus MG132 versus 13.0  ±  0.9 in Rapa) (Figure 5E,F). Moreover, we did not observe any significant difference in the fluorescence levels of EGFP-AaAtg8 between the Rapa treatment group and the Rapa plus either BafA1 or MG132 treatment group (Figure 5E,G). The fluorescent puncta levels were consistent with the expression trends of the endogenous AaSQSTM1 and AaAtg8 proteins observed by Western blotting. In addition, we observed that yellow fluorescence puncta are increased in the Rapa treatment and subsequently decreased after adding either Baf A1 or MG132 (Figure 5E), which is consistent with the colocalization of green fluorescence and red fluorescence by Pearson’s coefficient analysis (Figure 5H).

**Figure 6:**
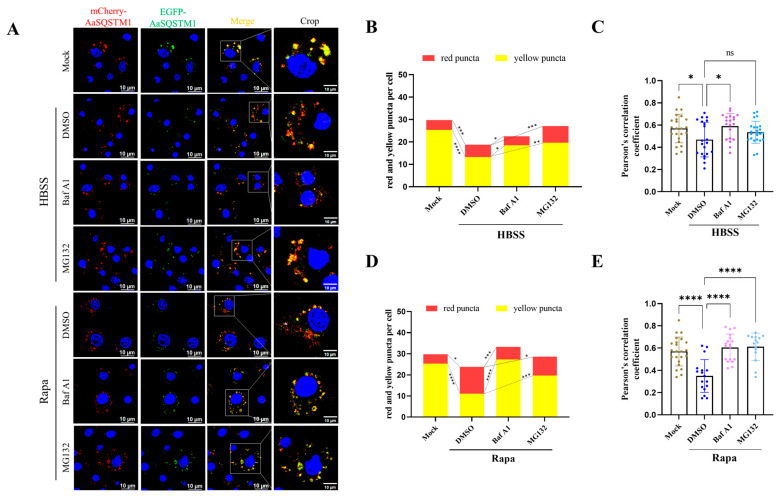
Dual fluorescence assay of mCherry-eGFP-AaSQSTM1 in autophagy-induced C6/36 cells. mCherry-EGFP-AaSQSTM1 plasmids were transfected into C6/36 cells for 48 h. In starvation-induced autophagy experiments, C6/36 cells were treated with HBSS, HBSS plus BafA1, or HBSS plus MG132 for 2 h. For the rapamycin-induced autophagy experiments, C6/36 cells were treated with rapamycin, rapamycin plus BafA1, or rapamycin plus MG132 for 6 h. C6/36 cells treated with nutrient-rich base medium were used as the mock control. (**A**) Cells were observed, and images of the cells were taken under a fluorescence microscope. DAPI was used to stain nuclear DNA, and the scale bar represents 10 μm. (**B**,**D**) The average numbers of red (mCherry) and yellow (Merge of EGFP and mCherry) fluorescent puncta in over 40 cells were determined using ImageJ. (**C**,**E**) The quantitative analysis of the Pearson’s colocalization coefficient of mCherry and EGFP puncta. The statistical significance was calculated by one-way ANOVA; ns represents a nonsignificant difference, * *p* < 0.05, ** *p* < 0.01, *** *p* < 0.001, **** *p* < 0.0001.

Dual-fluorescence-labeled LC3/Atg8 has been successfully applied to assess the populations of autophagosomes and autolysosomes [35]. To explore whether dual-fluorescence-labeled AaSQSTM1 can be used to monitor autophagy flux, the expression vector pIB- mCherry-EGFP-AaSQSTM1 was used to detect autophagy activity in C6/36 cells. The number of mCherry puncta (red fluorescence indicates autolysosomes) and the merging of mCherry and EGFP puncta (yellow flu indicates autophagosomes) were observed by fluorescence microscopy. In mCherry-EGFP-AaSQSTM1-overexpressing C6/36 cells, the numbers of red fluorescent and yellow puncta were lower in the HBSS treatment group than in the mock control group (red puncta: 18.8  ±  0.8 versus 29.7 ± 1.7; yellow puncta: 13.3  ±  0.8 versus 25.4  ±  1.4) (Figure 6A,B). Compared with HBSS treatment, HBSS plus BafA or MG132 treatment increased the numbers of red and yellow fluorescent puncta (red puncta: 23.3  ±  1.0 for BafA1, 27.1  ±  1.7 for MG132; yellow puncta: 18.5  ±  1.2 for BafA1, 19.7  ±  1.3 for MG132). Similarly to HBSS treatment, Rapa treatment also decreased the numbers of red and yellow fluorescent puncta compared with the mock control (red puncta: 23.9  ±  1.1 versus 29.7  ±  1.7; yellow puncta: 11.2  ±  1.0 versus 25.4  ±  1.4). Moreover, compared with DMSO treatment, Rapa plus BafA1 or MG132 treatment increased the numbers of red and yellow fluorescent puncta (red puncta: 33.2  ±  1.7 for BafA1, 28.7  ±  2.1 for MG132; yellow puncta: 27.2  ±  1.9 for BafA1, 19.7  ±  1.8) (Figure 6A,D). Meanwhile, we observed that the colocalization of mCherry and EGFP puncta is significantly decreased in both HBSS and Rapa treatments, but restored after adding BafA1 (Figure 6C,E). Our results suggest that analysis of fluorescent puncta by using the mCherry-EGFP-AaSQSTM1-overexpressing system provides a useful method for studying autophagy flux in mosquito cells.

## 4. Discussion

Autophagy is involved in diverse fundamental biological processes, including development, aging, cell death, and defense against pathogens [4,22,36]. In selective autophagy, autophagy receptors bind with several specific cargos, including aggregated proteins, dysfunctional mitochondria, and invading pathogens, and facilitate cargo turnover [37]. SQSTM1(also known as p62) is one of the most important selective autophagy receptors identified in metazoans; it links the cargo to the autophagic machinery and elicits directed autophagosome formation [9]. In this study, we identified the homolog of SQSTM1 (AaSQSTM1) in *Ae. albopictus* and investigated the spatial-temporal expression characteristics of AaSQSTM1 as well as its function in monitoring autophagy activity in mosquito cells.

SQSTM1 is highly evolutionarily conserved across invertebrates, such as *C. elegans* and *D. melanogaster*, to vertebrate species, including *H. sapiens* and *M. musculus* [8,9,24]. BLAST revealed that AaSQSTM1 shares highly conserved domains with mammalian SQSTM1 and *Drosophila* Ref(2)P, including the PB1, UBA, and LIR/AIM domains. SQSTM1 acts as an autophagy receptor through the above domain interactions to mediate the autophagy process [11]. First, SQSTM1 interacts directly with selected cargoes by using its C-terminal UBA domain. Subsequently, the LIR/AIM domain interacts with ATG8s anchored via their lipid tails to the inner membrane of the phagophore [9]. Through sequence analysis and 3D model prediction, we predicted that the “DDD” and “WTML” residues in the LIR/AIM domain of AaSQSTM1 are involved in binding with AaAtg8; however, further functional experiments are needed for confirmation. In addition, the homopolymerization of SQSTM1 mediated by its PB1 domain facilitates its coaggregation with the cargo [10,12]. Polymerization also enables a tight interaction of the SQSTM1-coated cargo with lipidated ATG8s at the phagophore [9]. Finally, SQSTM1, Atg8, and cellular components or cargos are sequestered into double-membrane vesicles called autophagosomes and subsequently recycled by lysosomal degradation [38].

Autophagy plays an important role in the lives of invertebrates from embryonic development to maturation [8]. In *Drosophila*, larval tissues undergo autophagic degradation during metamorphosis [20]. SQSTM1/ Ref(2)P maintains the cellular homeostasis through the removal of protein aggregates and dysfunctional mitochondria [8,39]. We found that AaSQSTM1 exhibited much higher expression levels in the adult stage than in the egg to pupa stages. In contrast, Atg8-II exhibited much higher expression levels from the egg to pupa stages than in the adult stages. The autophagic activity was upregulated when the expression level of p62/SQSTM1 was decreased and that of Atg8-II was increased [14]. Our results suggested that AaSQSTM1 and AaAtg8 might be involved in *Ae. albopictus* mosquito development by regulating autophagic activity. The inhibition of autophagy activity in *D. melanogaster* is lethal at the larval stage, indicating that autophagy is essential for insect development [39]. Recent studies indicated that there is a correlation between the levels of SQSTM1/Ref(2)P and the longevity in both *D. melanogaster* and *C. elegans* [8,26], indicating that SQSTM1 is a potent target for controlling mosquito development. We believed that the functional validation of AaSQSTM1 would be significant for understanding its role in the lifespan of *Ae. albopictus* mosquito.

Autophagy is critical for proper nutrient utilization during *Drosophila* development and oogenesis [40,41]. Blood meal supplies the necessary nutrients for female mosquitoes to promote ovary development and oogenesis. Autophagy enables the fat body to adapt to insect energy metabolism by degrading and recycling proteins, lipids, and carbohydrates [37,42]. In this study, we observed that blood-feeding decreased the mRNA level of the SQSTM1 gene and increased the expression of the Atg8-II gene in the fat body, which indicated that blood meals induce autophagy in the fat body. Moreover, autophagy plays an important role in maintaining egg maturation cycles in female mosquitoes [32]. Blood meals increased both the mRNA and protein levels of the SQSTM1 and Atg8 genes in female ovaries, suggesting a possible role in regulating ovary development after blood feeding. However, further investigations are needed to elucidate the role of AaSQSTM1 in mosquito production.

*Aedes* Mosquitoes (mainly *Ae. aegypti* and *Ae. albopictus*) are the main vectors for transmitting several serious arboviral diseases, such as Dengue and Zika [22]. Autophagy plays an important role in regulating arbovirus infection in mosquito vector [7,21,22]. Recent studies have proved that arboviruses including DENV and ZIKA exploited autophagy to facilitate the viral replication [31,43]. Meanwhile, the activation of autophagy pathway inhibited DENV replication in *Ae. aegypti* cells [44]. Autophagy is a continuous process including the formation of autophagosomes and the fusion of lysosomes to autolysosomes [6]. Therefore, monitoring autophagy flux will be helpful for elucidating the interaction of arboviruses and mosquito’s autophagy. Until now, there are limited reporting tools for studying autophagic flux in mosquito cells [30]. In this study, we applied AaSQSTM1-based assay methods to monitor autophagy activity in C6/36 cells. The protein levels of AaSQSTM1 and AaAtg8 were decreased after HBSS treatment, suggesting autophagy was activated under starvation. Rapamycin, as a specific mTOR inhibitor, could efficiently activate autophagy in mosquito cells [30]. We observed the endogenous AaSQSTM1 and Atg8-II protein levels were decreased and increased individually after Rapamycin treatment, which is consistent with the previous studies in *Drosophila* and mammal cells [45,46,47].

Furthermore, we analyzed the fluorescent puncta in C6/36 cells which were co-transfection of mCherry-AaSQSTM1 and EGFP-AaAtg8. The number of red and green fluorescent puncta represents the expression levels of mCherry-AaSQSTM1 and EGFP-AaAtg8 individually. We observed that the fluorescent puncta under varied treatment were consistent with the expression trends of the endogenous AaSQSTM1 and AaAtg8 proteins observed by Western blotting. BafA1 and MG132 have different impacts on the turnover of AaSQSTM1 and AaAtg8. BafA1 suppressed the degradation of AaAtg8 by blocking the fusion of autophagosomes and lysosomes, whereas MG132 inhibited the degradation of AaSTSTM1 by blocking the ubiquitin-proteasome pathway. MG132 was also applied to activate autophagy in mammal cells [48]. We demonstrated that the protein expression assays of AaSQSTM1 and AaAtg8 under the treatment of BafA1 or MG132 can be applied to explain the turnover of autophagy flux in C6/36 cells. Additionally, we developed a dual fluorescent reporter plasmid mCherry-EGFP-AaSQSTM1 to monitor autophagy flux. EGFP emits strong fluorescence at neutral pH in autophagosomes, but the fluorescence is quenched at acidic pH in autolysosomes. Hence, any autophagosomes that fuse with lysosomes will lose the green fluorescent signal while retaining the red fluorescent signal [49]. Our study revealed that C6/36 cells expressing mCherry-EGFP-AaSQSTM1 reflected the real-time state of autophagosome and lysosome fusion, thus providing a useful system to monitor the autophagy flux in mosquito cells.

## 5. Conclusions

Our study successfully identified the autophagy receptor SQSTM1 homolog protein AaSQSTM1 in *Ae. albopictus* and analyzed its functional domain and phylogenetic characteristics. Our results further indicate that the expression levels of AaSQSTM1 and AaAtg8 vary across different developmental stages and female tissues. In addition, we demonstrated that AaSQSTM1 regulates the autophagic process with AaAtg8, which can be applied to monitor autophagic activity by assaying endogenous protein levels and overexpressing fluorescent reporters at the cellular level. These findings provide a basis for understanding the function of AaSQSTM1 in regulating *Ae. albopictus* autophagic activity. Future research should further clarify the roles of AaSQSTM1 in regulating mosquito development, metabolism, and lifespan to develop strategies for vector control.

## Data Availability

The original contributions presented in this study are included in the article/Supplementary Material. Further inquiries can be directed to the corresponding authors.

## Supplementary Materials

The following supporting information can be downloaded at: https://www.mdpi.com/article/10.3390/insects16100994/s1, Figure S1. Amino acid sequence alignment of AaSQSTM1 and homologous p62/SQSTM1 proteins. Red highlights indicate all identical residues; yellow highlights indicate the majority of conserved residues (black bold text indicates the conserved residues). The amino acid sequences of p62/SQSTM1 were derived from *Ae. albopictus*, *Ae. aegypti*, *An. gambiae*, *Cx. quinquefasciatus, Cx. pipiens pallens*, *B. mori*, *D. melanogaster*, *C. elegans*, *H. sapiens* and *M. musculus*. The amino acid sequences were aligned using ESPript 3.0. Figure S2. Phylogenetic neighbor-joining tree of SQSTM1. SQSTM1 from *Ae. albopictus* (AaSQSTM1) and other representative species: *Ae. aegypti*, *An. gambiae*, *Cx. quinquefasciatus, Cx. pipiens pallens*, *B. mori*, *D. melanogaster*, *C. elegans*, *H. sapiens* and *M. musculus*. AaSQSTM1 is indicated with red triangle. The phylogenetic tree of SQSTM1 proteins, which are available on GenBank, was constructed using the neighbor-joining method in MEGA 11.0. Figure S3. Western blot analysis of AaSQSTM1 in *Ae. albopictus* mosquito and C6/36 cell line samples. The protein samples from C6/36 cells and adult mosquitoes (Female and Male) were submitted for SDS-PAGE and Western blot analysis. Bovine serum albumin (BSA) was taken as the negative control (NC). Figure S4. Schematic diagram of the recombinant vector (pIB-mCherry-AaSQSTM1, pIB-EGFP-AaAtg8, and pIB-mCherry-EGFP-AaSQSTM1) construction. Table S1: RT-qPCR primers for this study. Figure S5: Original Western blot images for Figure 2; Figure S6: Original Western blot images for Figure 3; Figure S7: Original Western blot images for Figure 4.

## Abbreviations

HBSS: Hank’s Balanced Salt Solution; Rapamycin: Rapa; SF: sugar feeding; BF: blood feeding.
