# Supplementary material for: Identification, Expression of AaSQSTM1 in Aedes albopictus and Its Autophagic Function Analysis
Source: Insects. 2025 Sep 24;16(10):994. doi: 10.3390/insects16100994 (PMC12564118; doi:10.3390/insects16100994)
Supplement: Supplementary file 1 [file insects-16-00994-s001.zip › Figure S1-S4, Table S1.pdf]

**Figure S1.** Amino acid sequence alignment of AaSQSTM1 and homologous p62/SQSTM1 proteins.

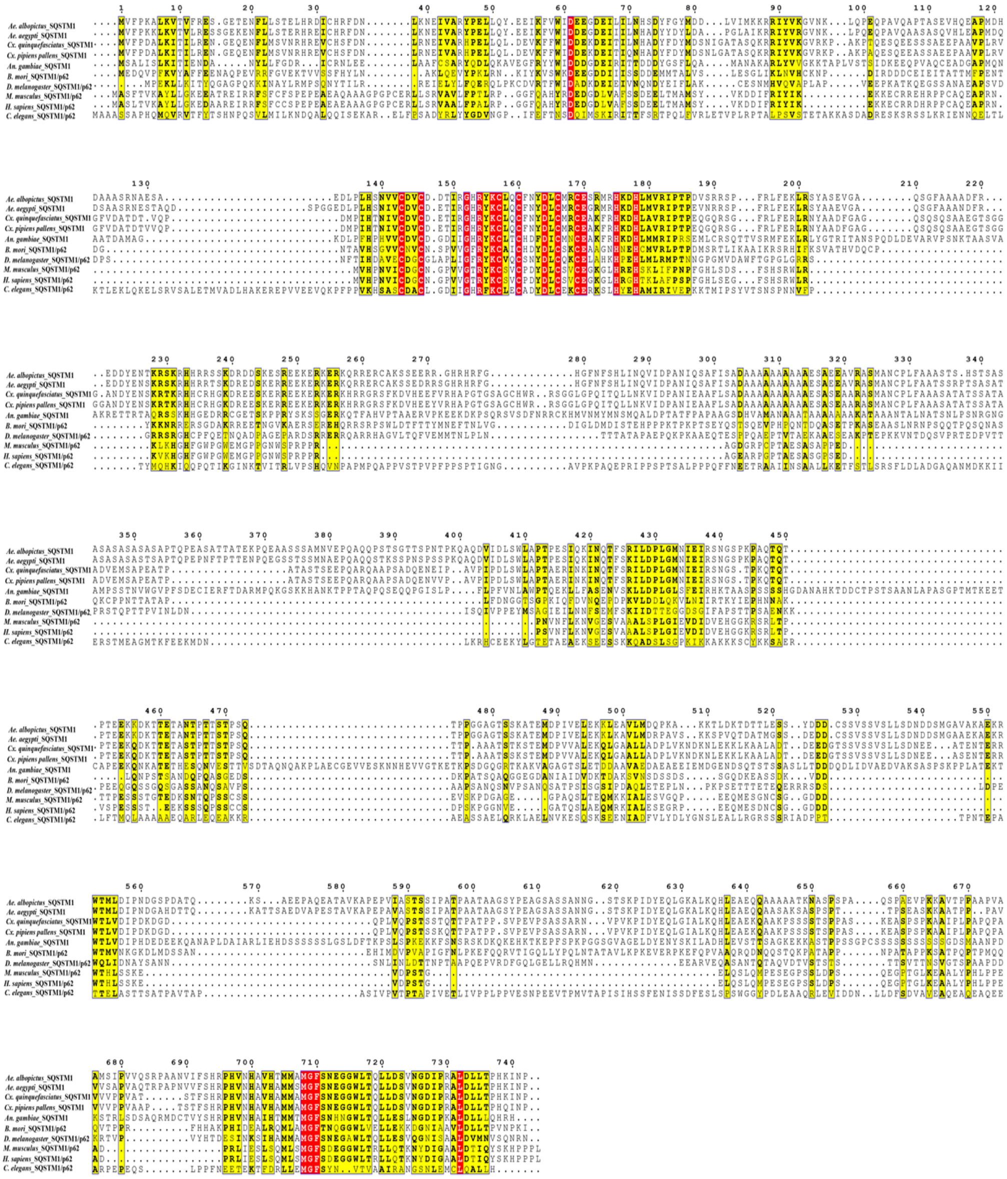

Red highlights indicate all identical residues; yellow highlights indicate the majority of conserved residues (black bold text indicates the conserved residues). The amino acid sequences of p62/SQSTM1 were derived from *Ae. albopictus*, *Ae. aegypti*, *An. gambiae*, *Cx. quinquefasciatus, Cx. pipiens pallens*, *B. mori*, *D. melanogaster*, *C. elegans*, *H. sapiens* and *M. musculus*. The amino acid sequences were aligned using ESPript 3.0.

**Figure S2.** Phylogenetic neighbor-joining tree of SQSTM1. SQSTM1 from *Ae. albopictus* (AaSQSTM1) and other representative species: *Ae. aegypti*, *An. gambiae*, *Cx. quinquefasciatus, Cx. pipiens pallens*, *B. mori*, *D. melanogaster*, *C. elegans*, *H. sapiens* and *M. musculus*.

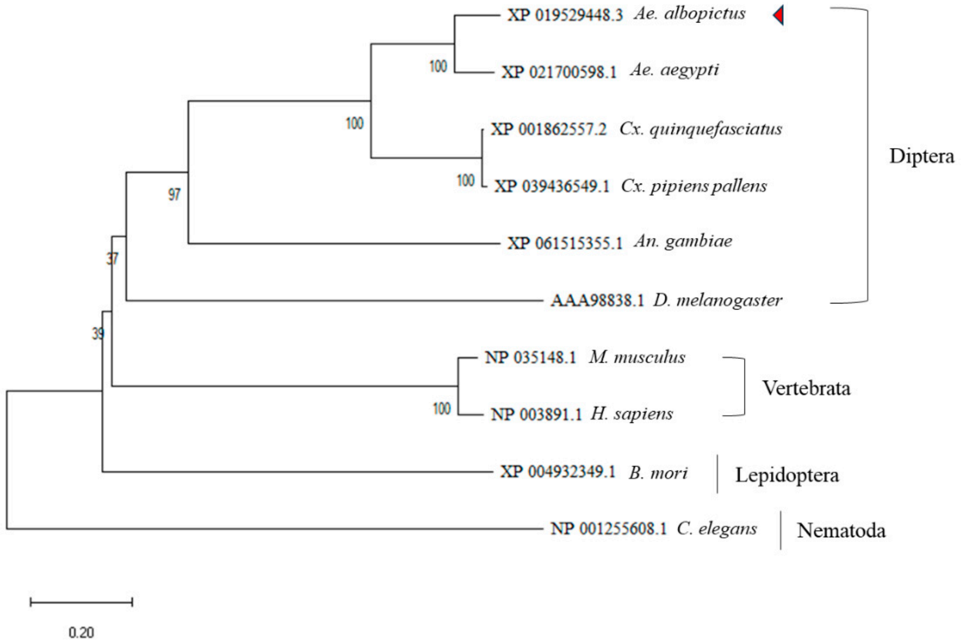


AaSQSTM1 is indicated with red triangle. The phylogenetic tree of SQSTM1 proteins, which are available on GenBank, was constructed using the neighbor-joining method in MEGA 11.0.

**Figure S3.** Western blot analysis of AaSQSTM1 in *Ae. albopictus* mosquito and C6/36 cell line samples.

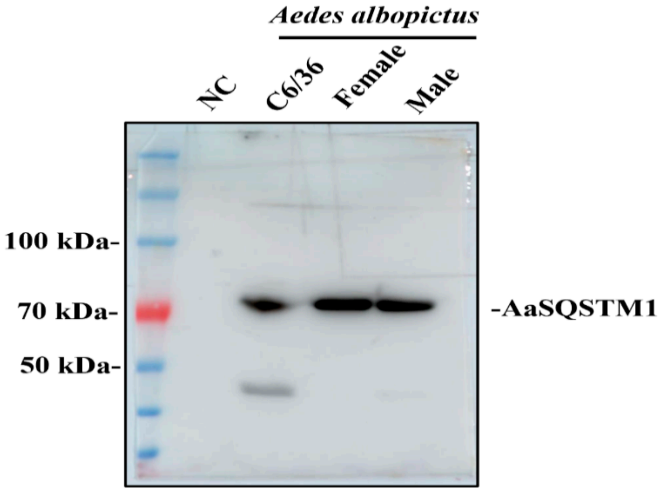

**Figure S4.** Schematic diagram of the recombinant vector (pIB-mCherry-AaSQSTM1, pIB-EGFP-AaAtg8, and pIB-mCherry-EGFP-AaSQSTM1) construction.

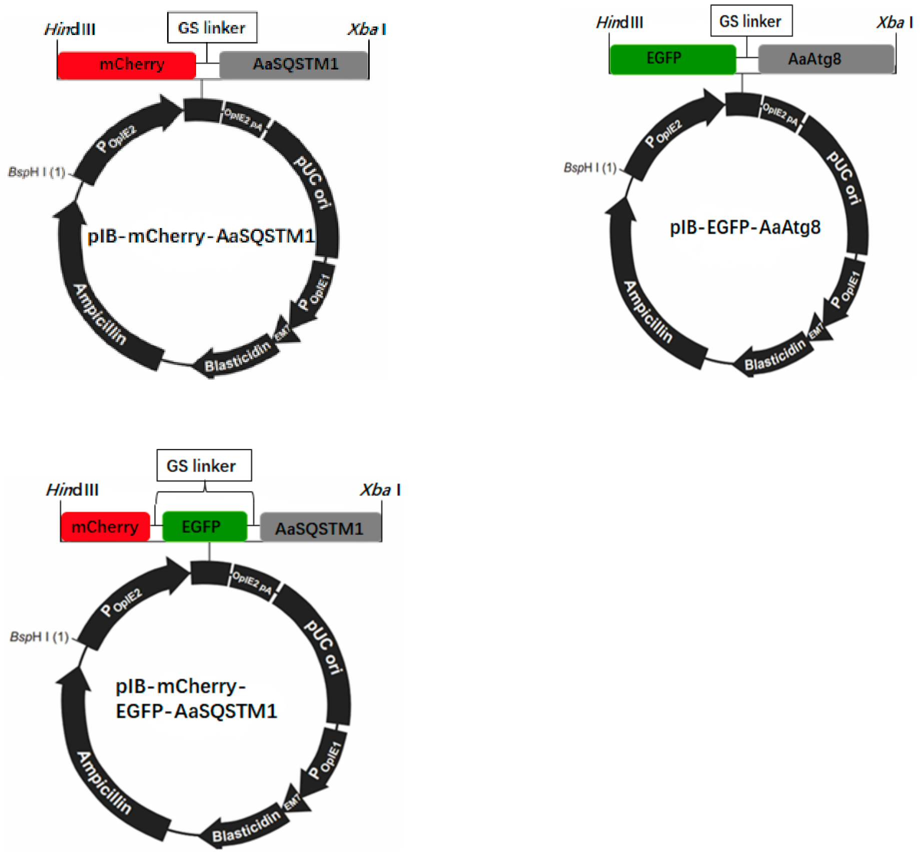

**Table S1.** The primer sequences for RT-qPCR analysis.

| Target genes | Primer Sequences |
|---|---|
| AaAtg8 | F: 5’- CGGAACGAGTACCCGTGAT-3’ |
| | R: 5’-AAATAGAACTGGCCGACGGT-3’ |
| AaSQSTM1 | F: 5’-GAAACTTCGCAGCTACGCCA-3’ |
| | R: 5’-TCATCGCGATCCTTGCTGGA-3’ |
| RPS7 | F: 5’-GAAGTTGTCGGAAAGCGTATGC-3’ |
| | R: 5’-TTCAATGGTGGTCTGCTGGTTC-3’ |

F: forward; R: reverse
