# Supplementary figures and images for: Identification, Expression of AaSQSTM1 in Aedes albopictus and Its Autophagic Function Analysis (part 2 of 2)
Source: Insects. 2025 Sep 24;16(10):994. doi: 10.3390/insects16100994 (PMC12564118; doi:10.3390/insects16100994)

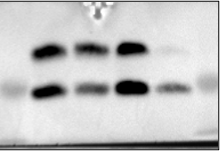

Supplement: Supplementary file 1 [file insects-16-00994-s001.zip › Figure S7/Figure 4A/3/HBSS+MG132---ATG8.tif]

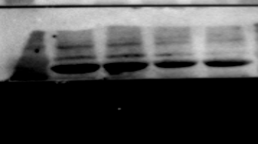

Supplement: Supplementary file 1 [file insects-16-00994-s001.zip › Figure S7/Figure 4A/3/HBSS+MG132---SQSTM1.tif]

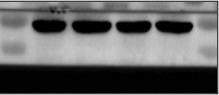

Supplement: Supplementary file 1 [file insects-16-00994-s001.zip › Figure S7/Figure 4A/3/HBSS+MG132-actin.tif]

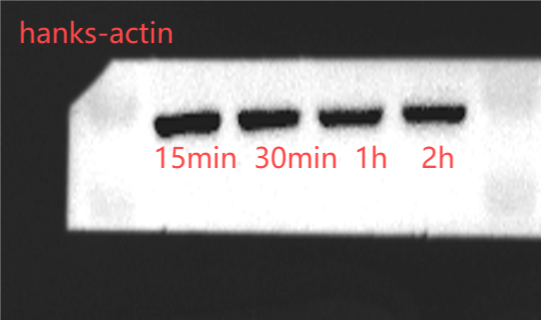

Supplement: Supplementary file 1 [file insects-16-00994-s001.zip › Figure S7/Figure 4A/3/HBSS-actin.png]

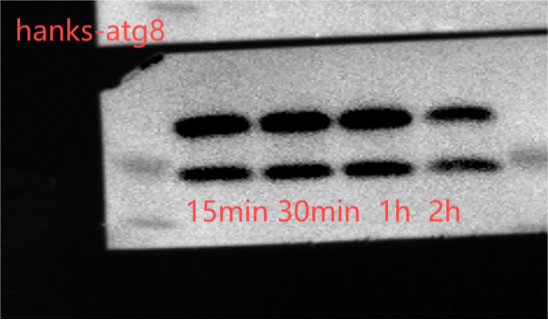

Supplement: Supplementary file 1 [file insects-16-00994-s001.zip › Figure S7/Figure 4A/3/HBSS-atg8.png]

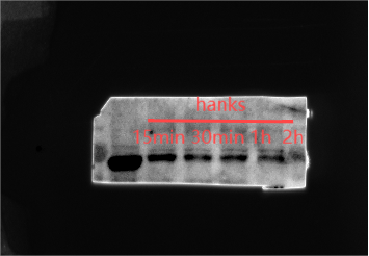

Supplement: Supplementary file 1 [file insects-16-00994-s001.zip › Figure S7/Figure 4A/3/HBSS-SQSTM1.png]

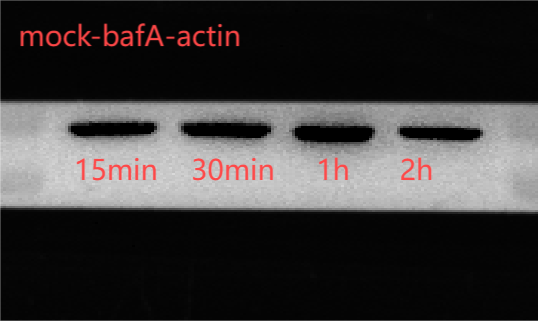

Supplement: Supplementary file 1 [file insects-16-00994-s001.zip › Figure S7/Figure 4A/3/mock+bafa actin.png]

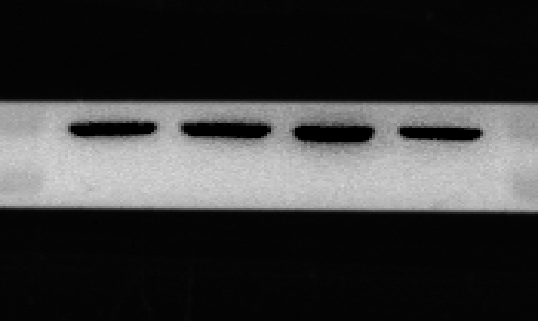

Supplement: Supplementary file 1 [file insects-16-00994-s001.zip › Figure S7/Figure 4A/3/mock+bafa actin.tif]

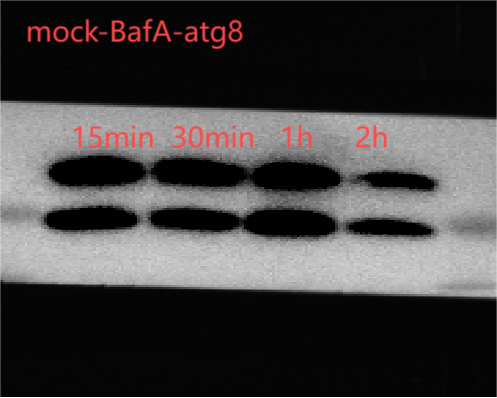

Supplement: Supplementary file 1 [file insects-16-00994-s001.zip › Figure S7/Figure 4A/3/mock+BafA-atg8.png]

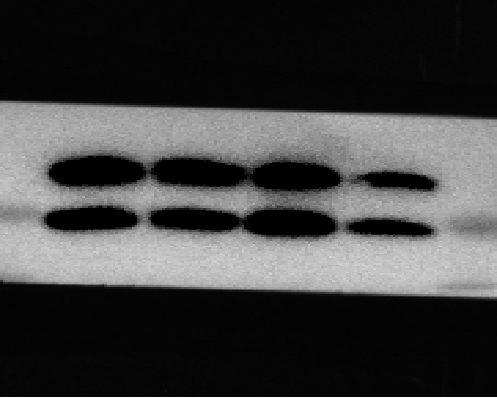

Supplement: Supplementary file 1 [file insects-16-00994-s001.zip › Figure S7/Figure 4A/3/mock+BafA-atg8.tif]

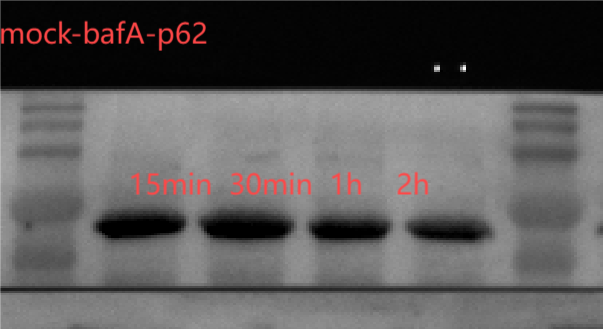

Supplement: Supplementary file 1 [file insects-16-00994-s001.zip › Figure S7/Figure 4A/3/mock+bafA-p62.png]

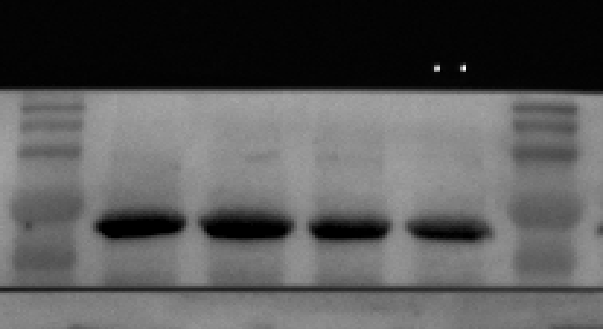

Supplement: Supplementary file 1 [file insects-16-00994-s001.zip › Figure S7/Figure 4A/3/mock+bafA-p62.tif]

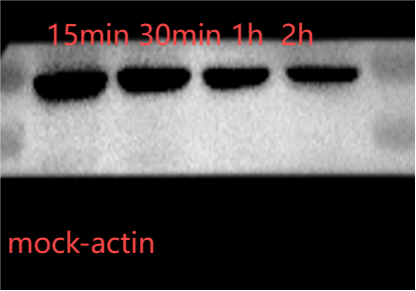

Supplement: Supplementary file 1 [file insects-16-00994-s001.zip › Figure S7/Figure 4A/3/mock-actin.png]

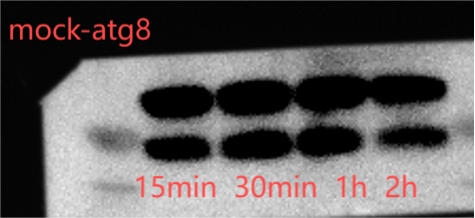

Supplement: Supplementary file 1 [file insects-16-00994-s001.zip › Figure S7/Figure 4A/3/mock-atg8.png]

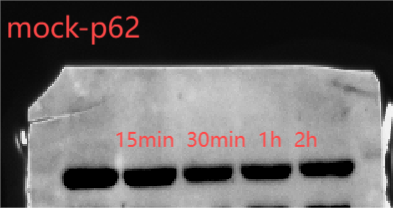

Supplement: Supplementary file 1 [file insects-16-00994-s001.zip › Figure S7/Figure 4A/3/mock-SQSTM1.png]

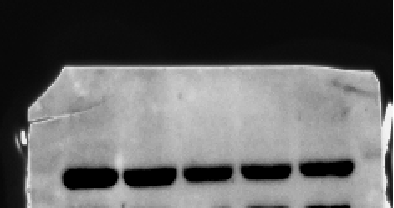

Supplement: Supplementary file 1 [file insects-16-00994-s001.zip › Figure S7/Figure 4A/3/mock-SQSTM1.tif]

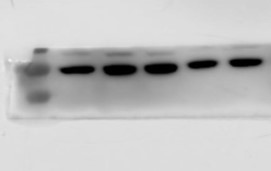

Supplement: Supplementary file 1 [file insects-16-00994-s001.zip › Figure S7/Figure 4D/1/ACTIN-rapa+baf.jpg]

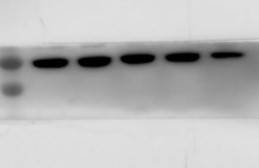

Supplement: Supplementary file 1 [file insects-16-00994-s001.zip › Figure S7/Figure 4D/1/ACTIN-rapa+mg132.jpg]

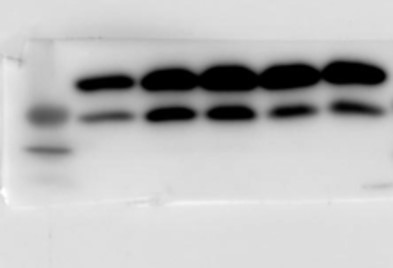

Supplement: Supplementary file 1 [file insects-16-00994-s001.zip › Figure S7/Figure 4D/1/ATG8-rapa+baf.jpg]

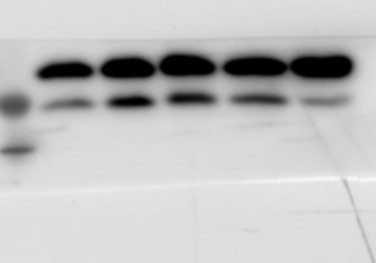

Supplement: Supplementary file 1 [file insects-16-00994-s001.zip › Figure S7/Figure 4D/1/ATG8-rapa+mg132.jpg]

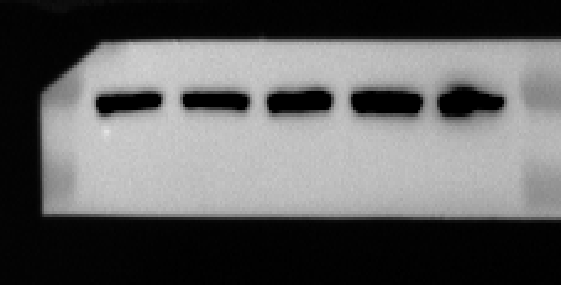

Supplement: Supplementary file 1 [file insects-16-00994-s001.zip › Figure S7/Figure 4D/1/mock-actin.tif]

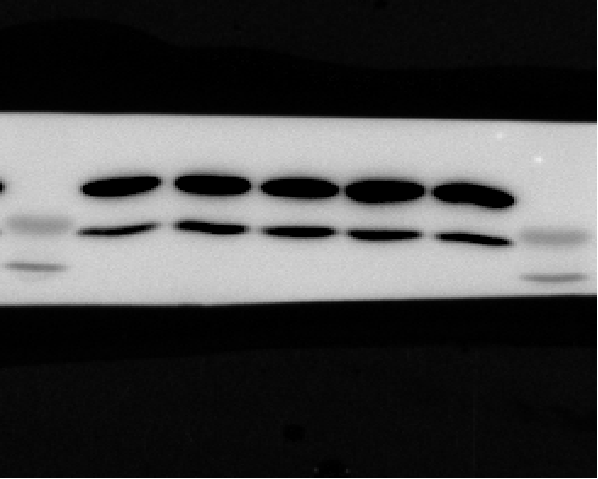

Supplement: Supplementary file 1 [file insects-16-00994-s001.zip › Figure S7/Figure 4D/1/mock-atg8.tif]

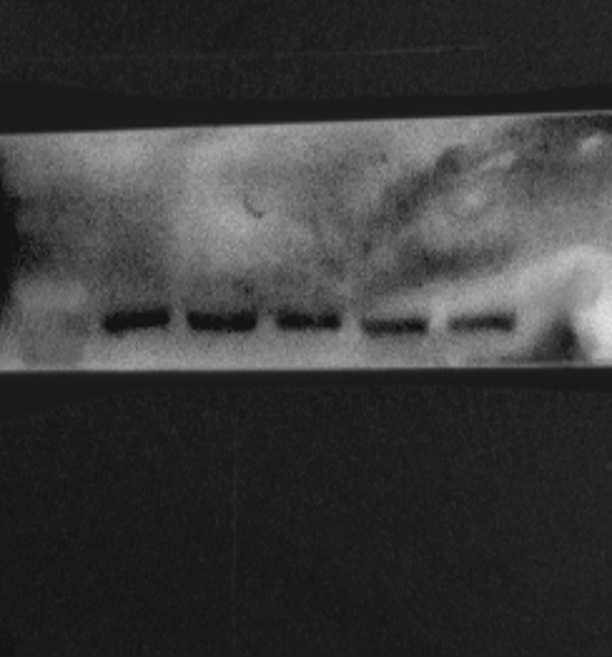

Supplement: Supplementary file 1 [file insects-16-00994-s001.zip › Figure S7/Figure 4D/1/mock-sqstm1.tif]

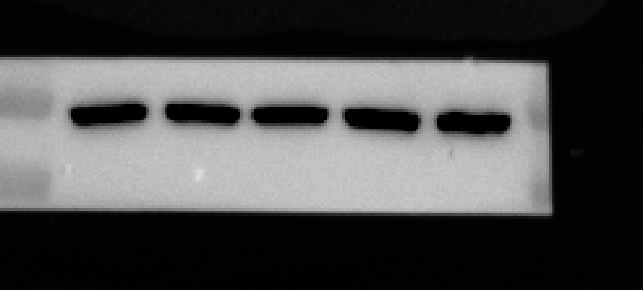

Supplement: Supplementary file 1 [file insects-16-00994-s001.zip › Figure S7/Figure 4D/1/rapa-actin.tif]

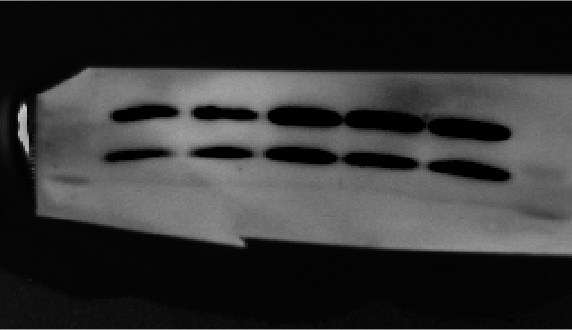

Supplement: Supplementary file 1 [file insects-16-00994-s001.zip › Figure S7/Figure 4D/1/rapa-atg8.tif]

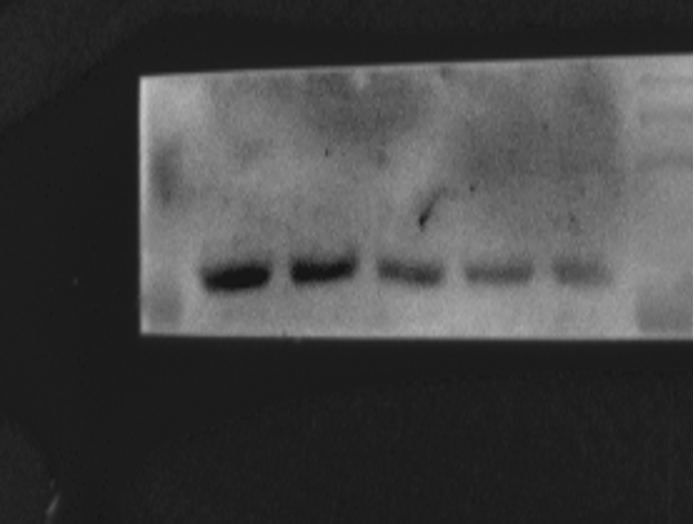

Supplement: Supplementary file 1 [file insects-16-00994-s001.zip › Figure S7/Figure 4D/1/rapa-sqstm1.tif]

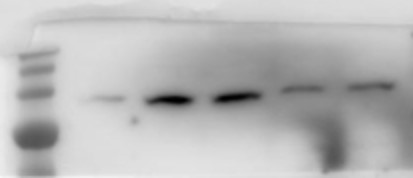

Supplement: Supplementary file 1 [file insects-16-00994-s001.zip › Figure S7/Figure 4D/1/SQSTM1-rapa+baf.jpg]

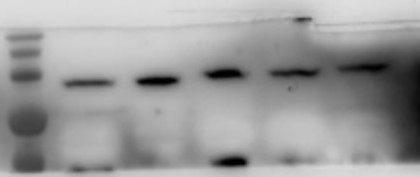

Supplement: Supplementary file 1 [file insects-16-00994-s001.zip › Figure S7/Figure 4D/1/SQSTM1-rapa+mg132.jpg]

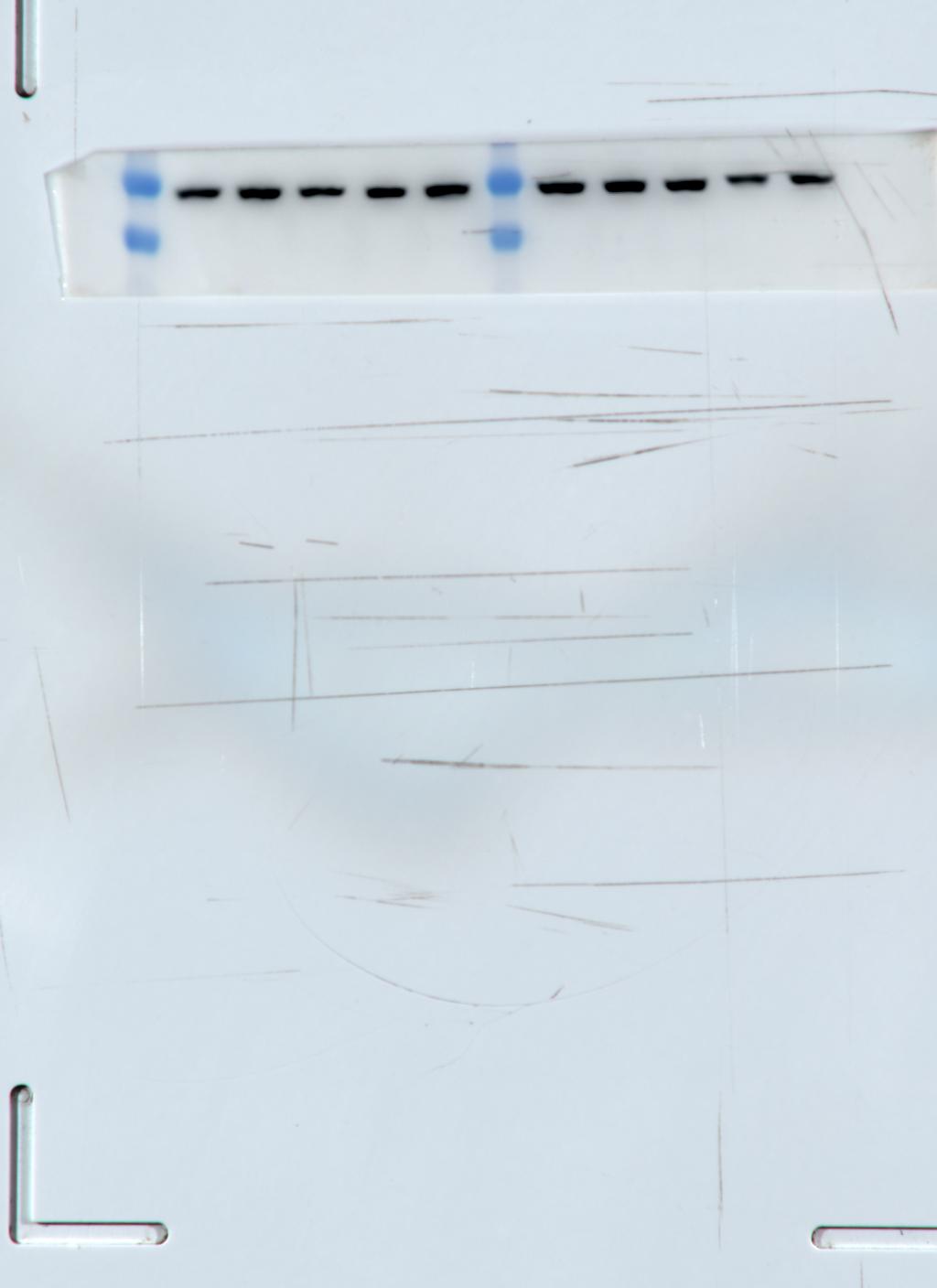

Supplement: Supplementary file 1 [file insects-16-00994-s001.zip › Figure S7/Figure 4D/2/actin-1 2025.03.04_14.29.17_Ch/actin-1 2025.03.04_14.29.17_Ch+Marker.jpg]

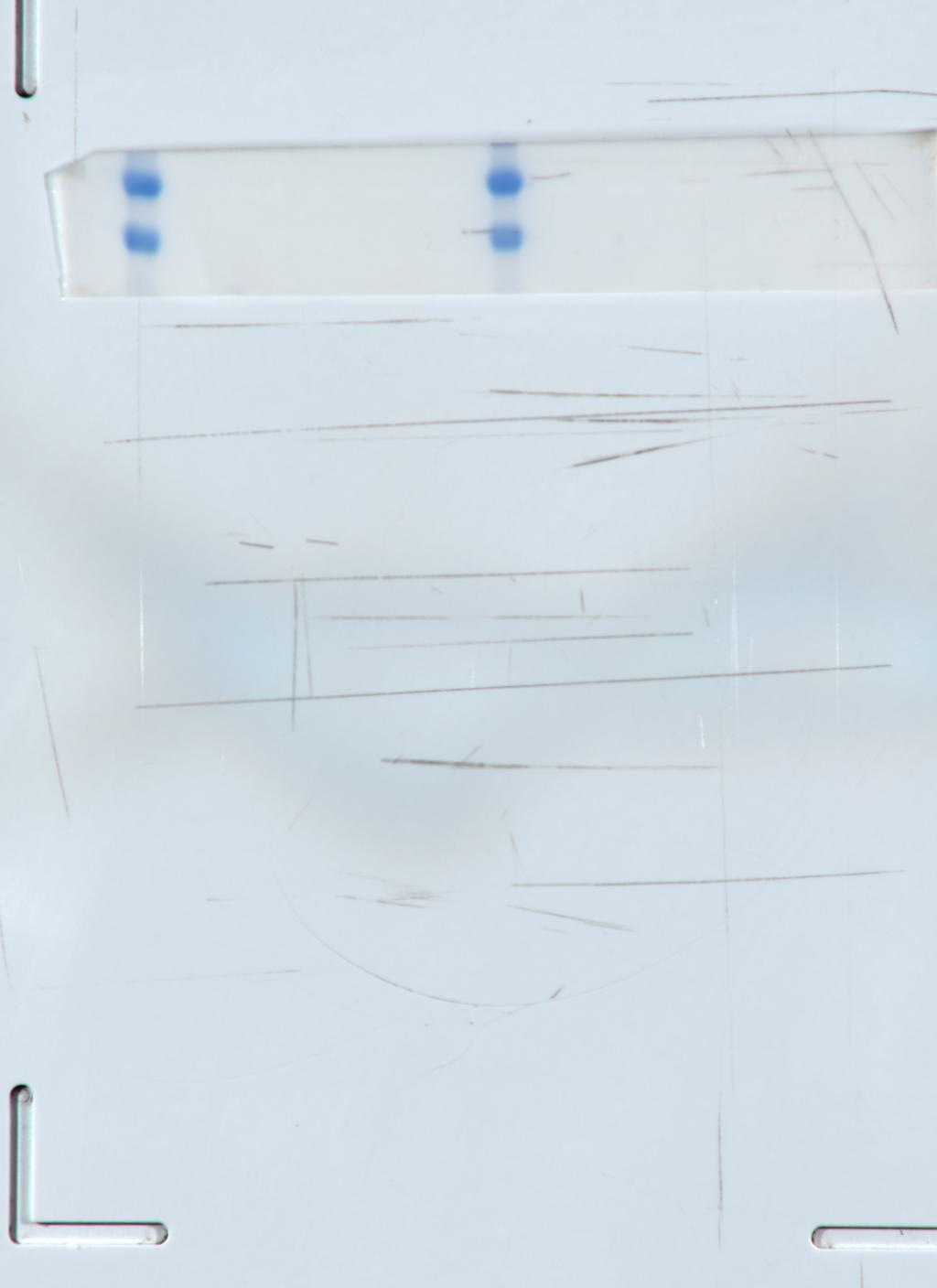

Supplement: Supplementary file 1 [file insects-16-00994-s001.zip › Figure S7/Figure 4D/2/actin-1 2025.03.04_14.29.17_Ch/actin-1 2025.03.04_14.29.17_Ch-Marker.jpg]

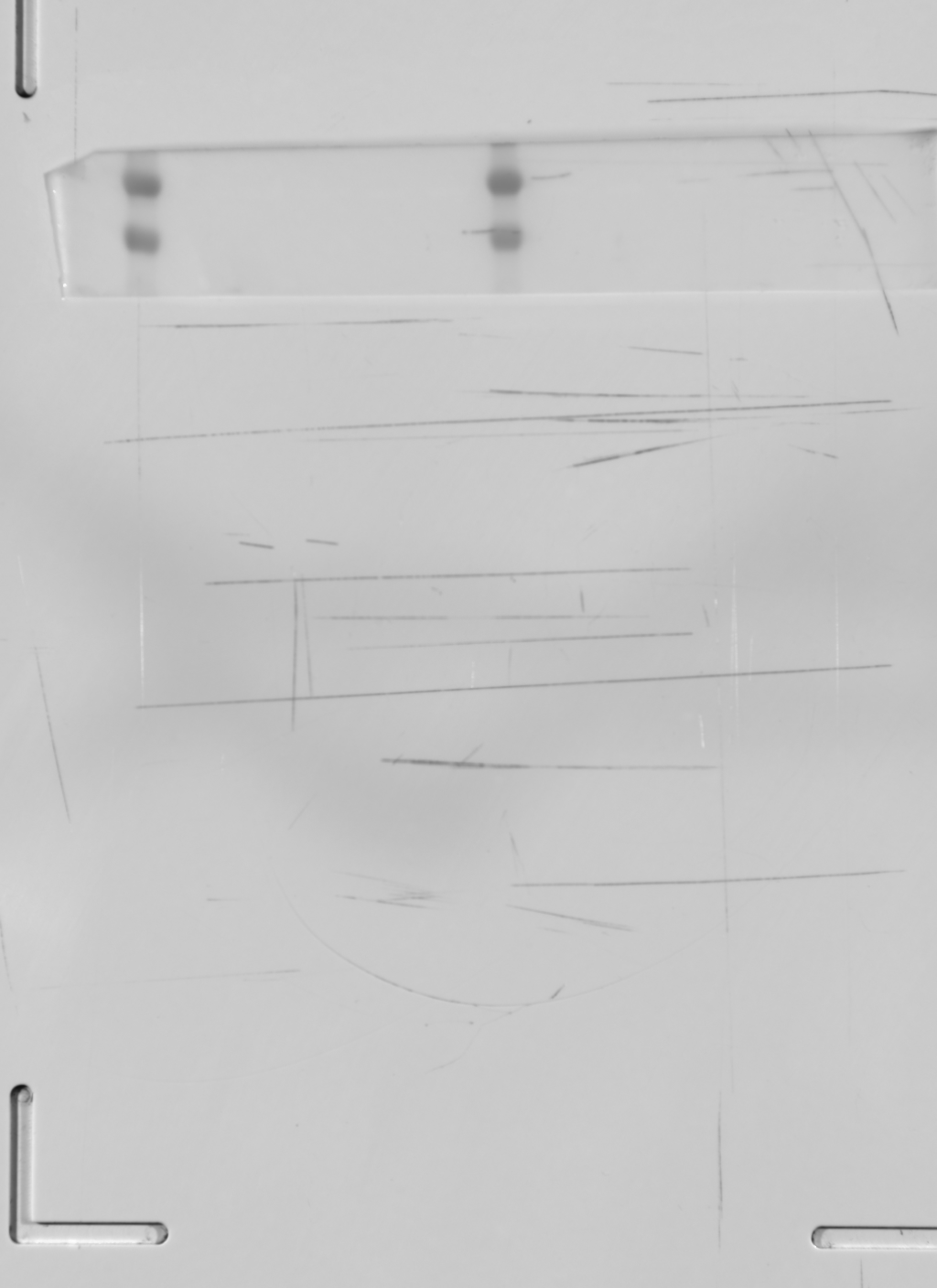

Supplement: Supplementary file 1 [file insects-16-00994-s001.zip › Figure S7/Figure 4D/2/actin-1 2025.03.04_14.29.17_Ch/actin-1 2025.03.04_14.29.17_Ch-Marker.tif]

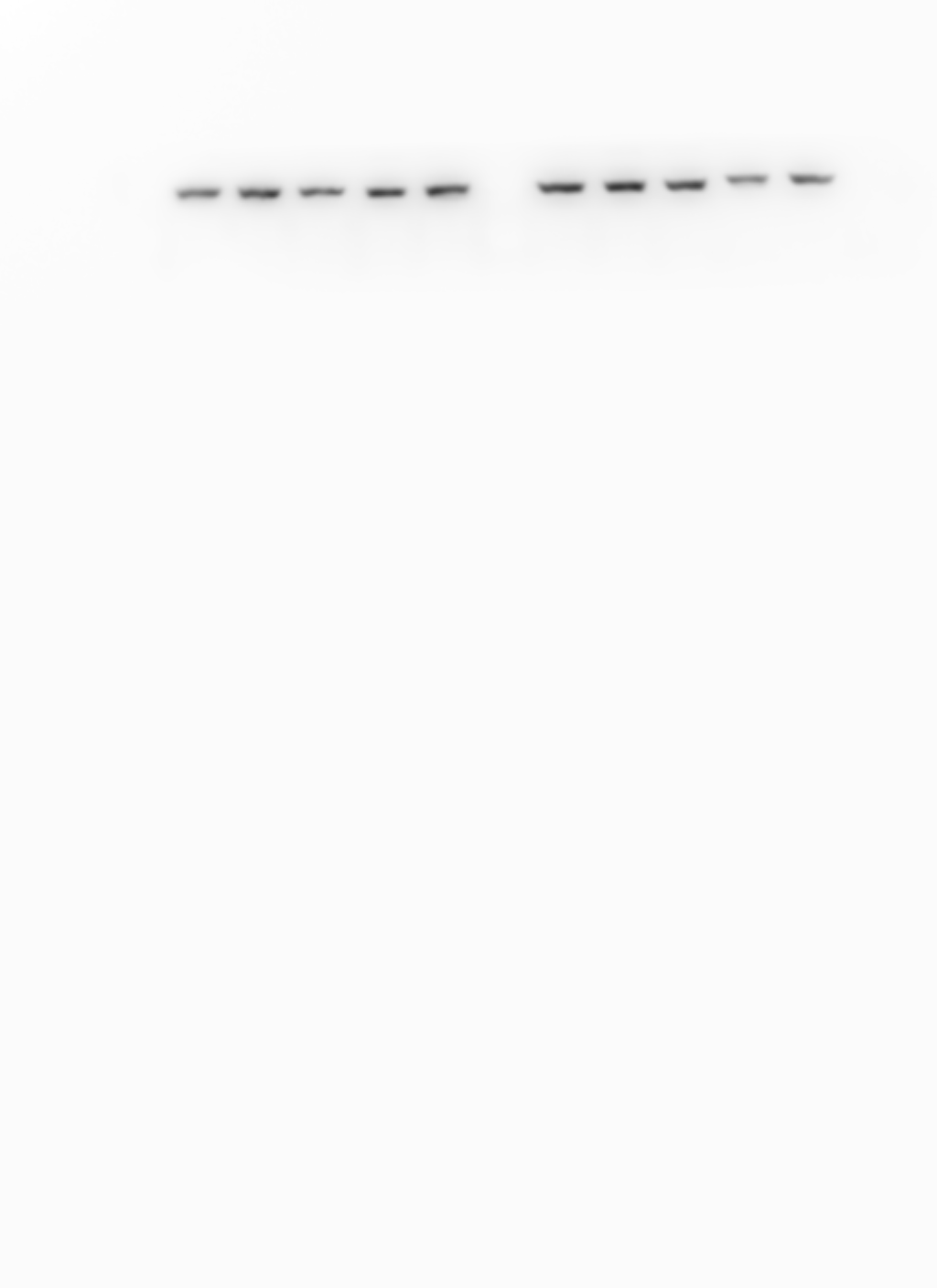

Supplement: Supplementary file 1 [file insects-16-00994-s001.zip › Figure S7/Figure 4D/2/actin-1 2025.03.04_14.29.17_Ch/actin-1 2025.03.04_14.29.17_Ch.tif]

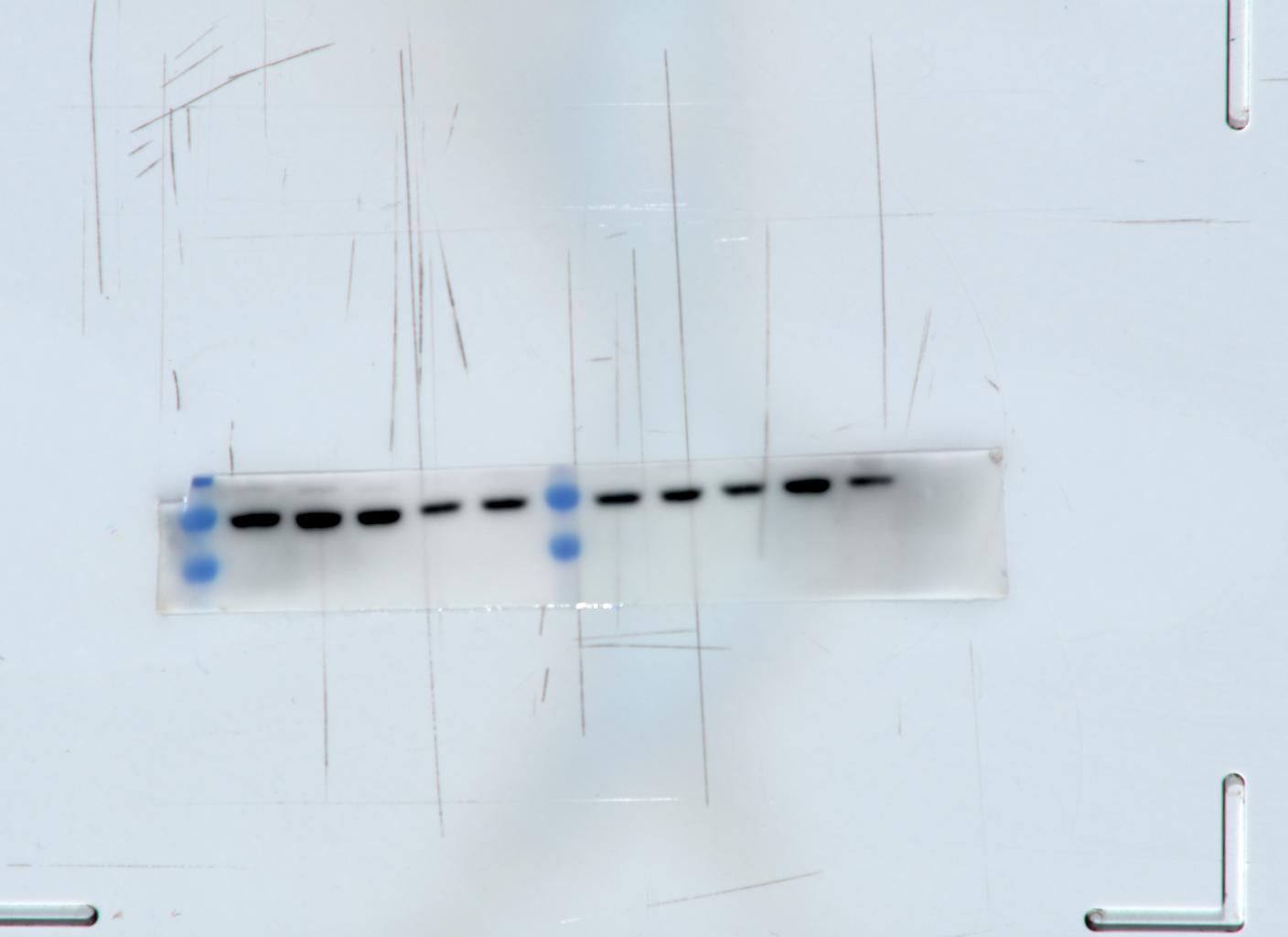

Supplement: Supplementary file 1 [file insects-16-00994-s001.zip › Figure S7/Figure 4D/2/actin-2 2025.03.04_14.15.16_Ch/actin-2 2025.03.04_14.15.16_Ch+Marker.jpg]

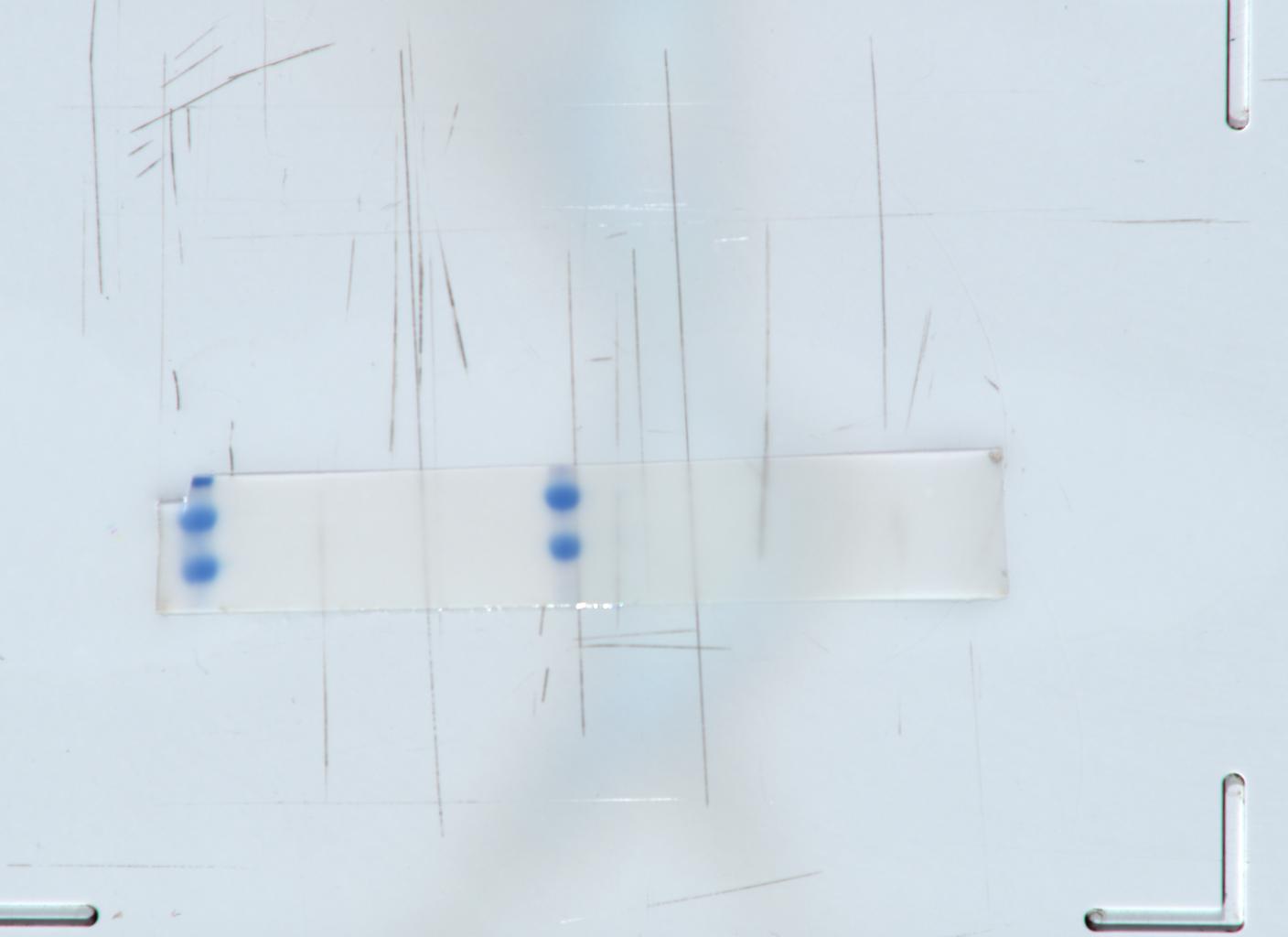

Supplement: Supplementary file 1 [file insects-16-00994-s001.zip › Figure S7/Figure 4D/2/actin-2 2025.03.04_14.15.16_Ch/actin-2 2025.03.04_14.15.16_Ch-Marker.jpg]

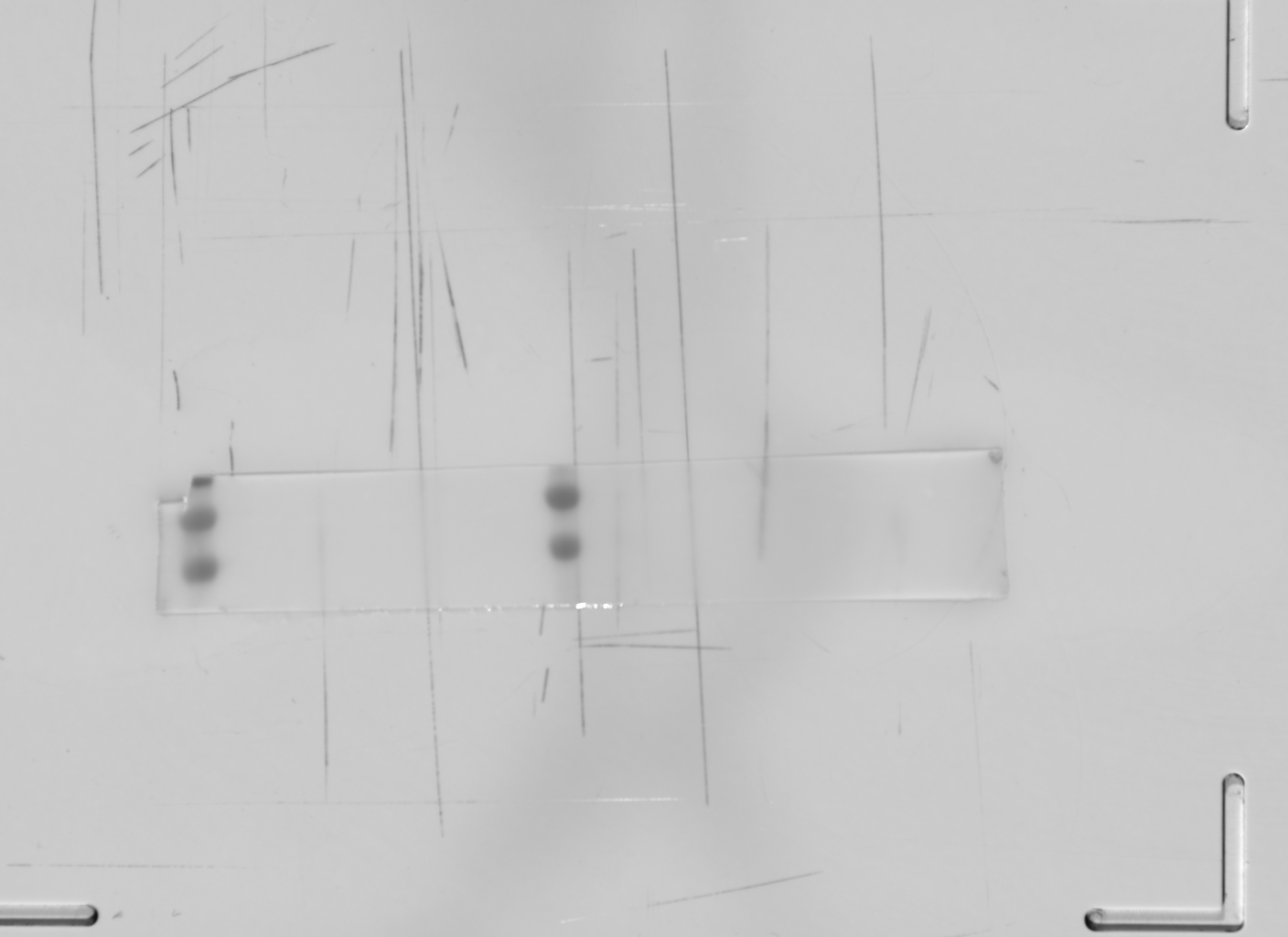

Supplement: Supplementary file 1 [file insects-16-00994-s001.zip › Figure S7/Figure 4D/2/actin-2 2025.03.04_14.15.16_Ch/actin-2 2025.03.04_14.15.16_Ch-Marker.tif]

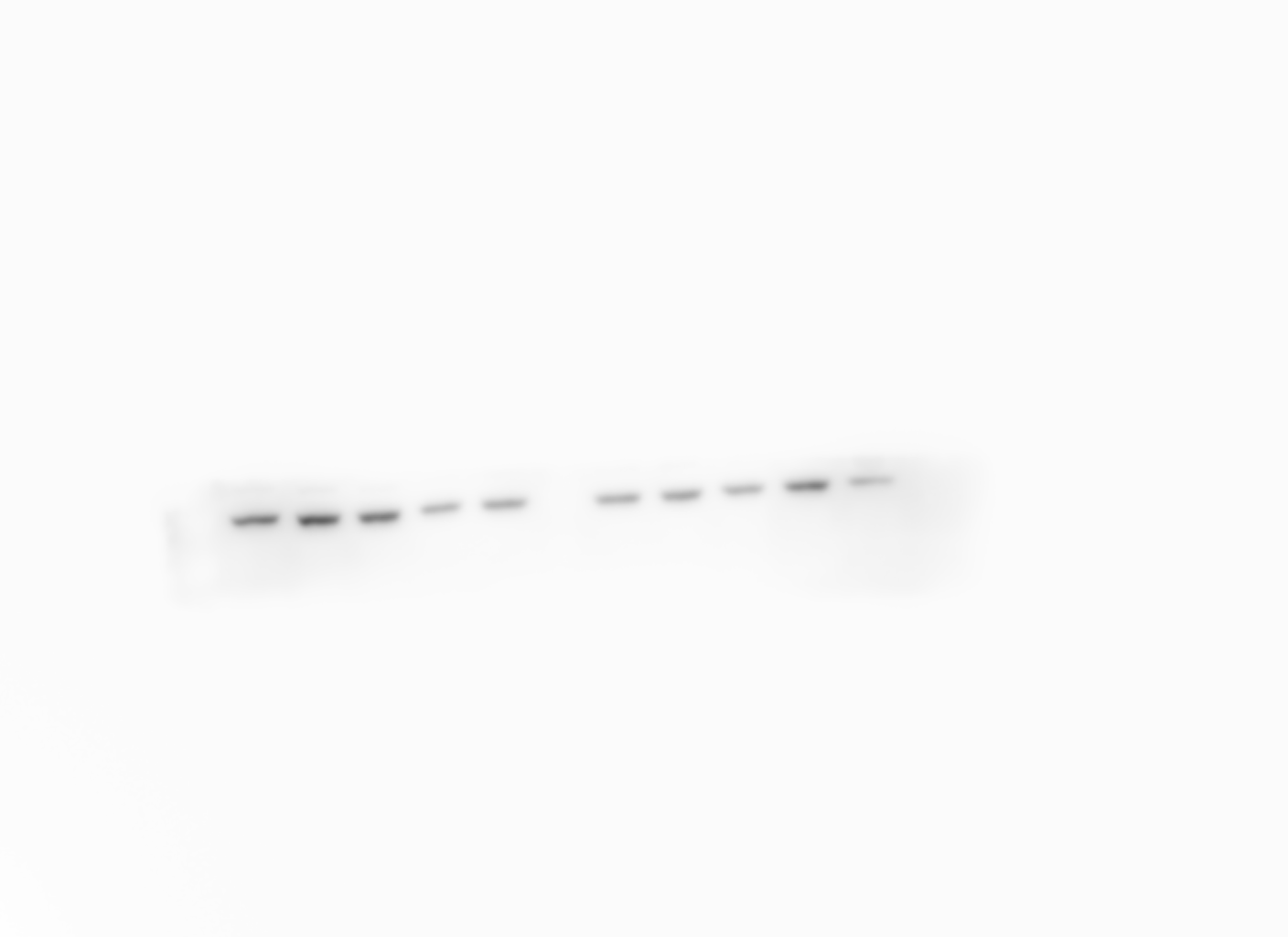

Supplement: Supplementary file 1 [file insects-16-00994-s001.zip › Figure S7/Figure 4D/2/actin-2 2025.03.04_14.15.16_Ch/actin-2 2025.03.04_14.15.16_Ch.tif]

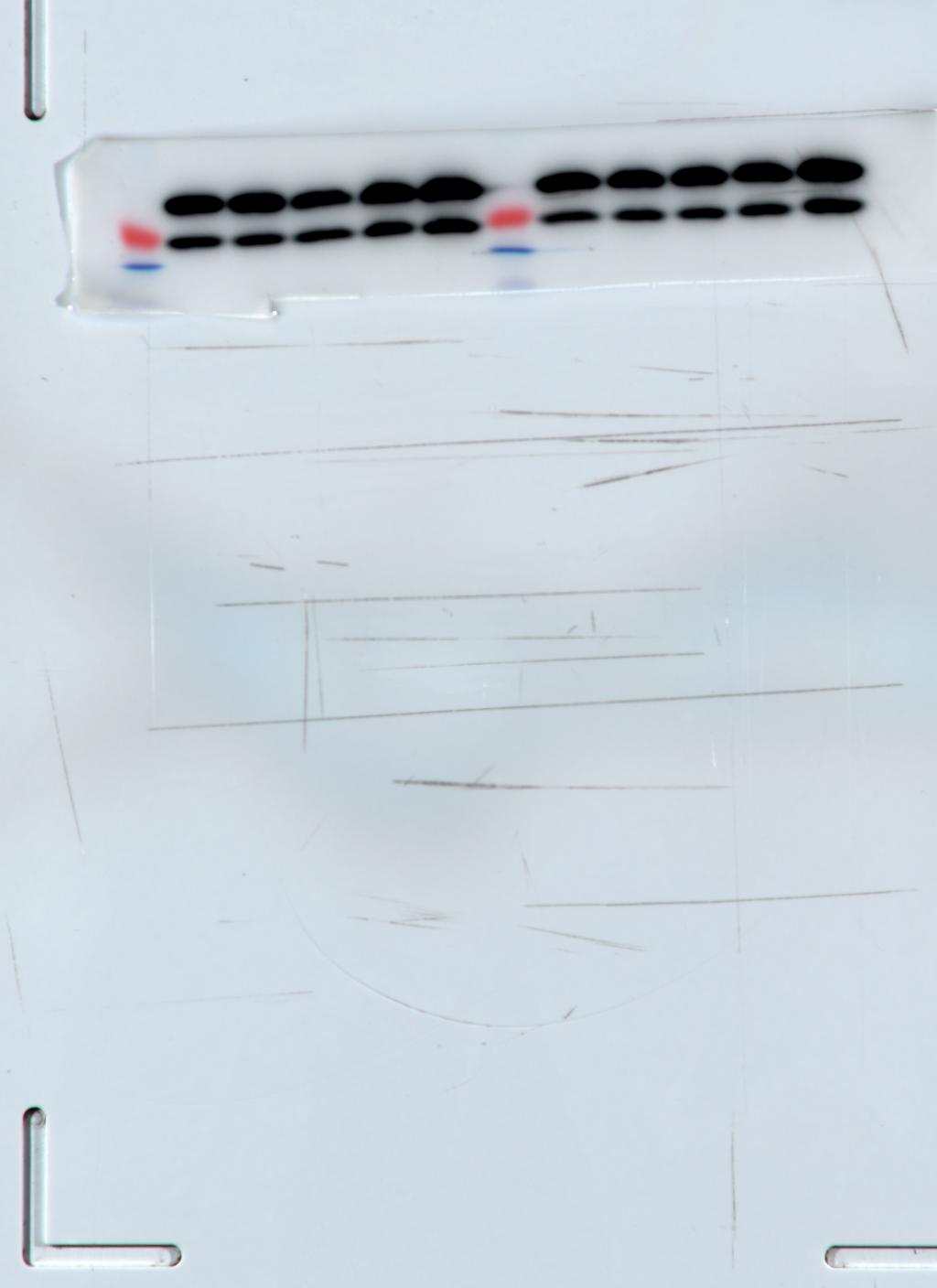

Supplement: Supplementary file 1 [file insects-16-00994-s001.zip › Figure S7/Figure 4D/2/atg8-1 2025.03.04_14.59.27_Ch/atg8-1 2025.03.04_14.59.27_Ch+Marker.jpg]

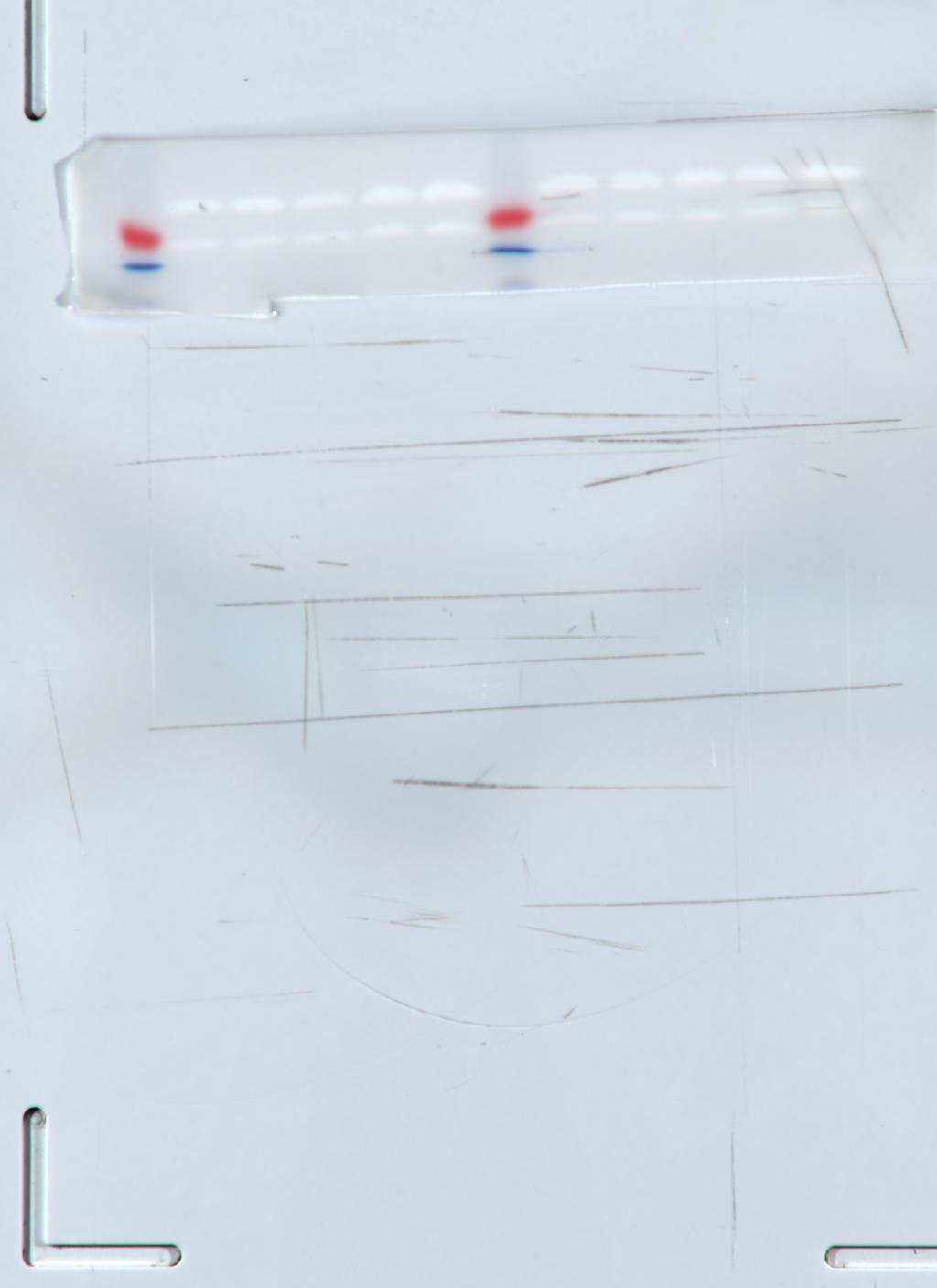

Supplement: Supplementary file 1 [file insects-16-00994-s001.zip › Figure S7/Figure 4D/2/atg8-1 2025.03.04_14.59.27_Ch/atg8-1 2025.03.04_14.59.27_Ch-Marker.jpg]

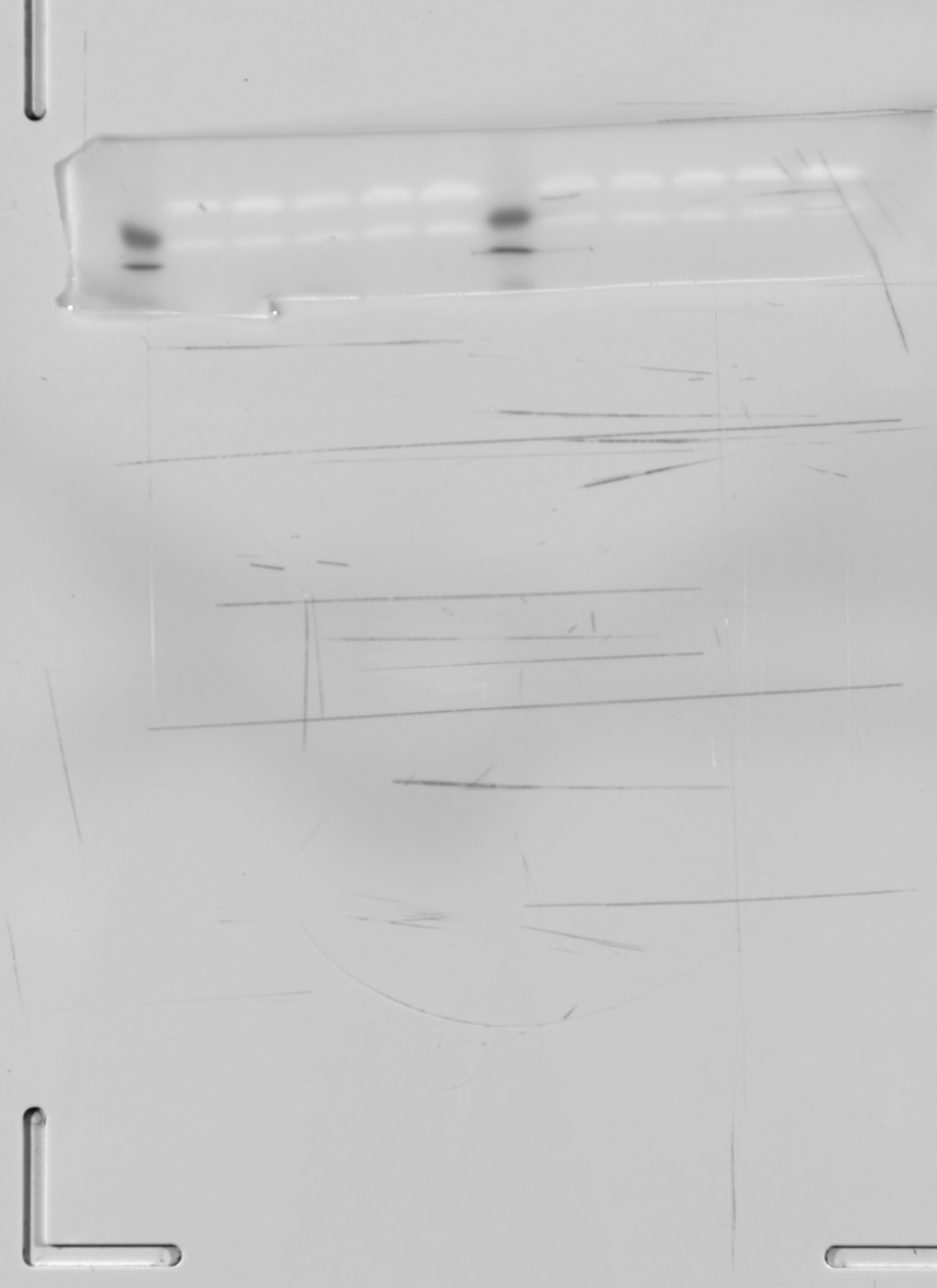

Supplement: Supplementary file 1 [file insects-16-00994-s001.zip › Figure S7/Figure 4D/2/atg8-1 2025.03.04_14.59.27_Ch/atg8-1 2025.03.04_14.59.27_Ch-Marker.tif]

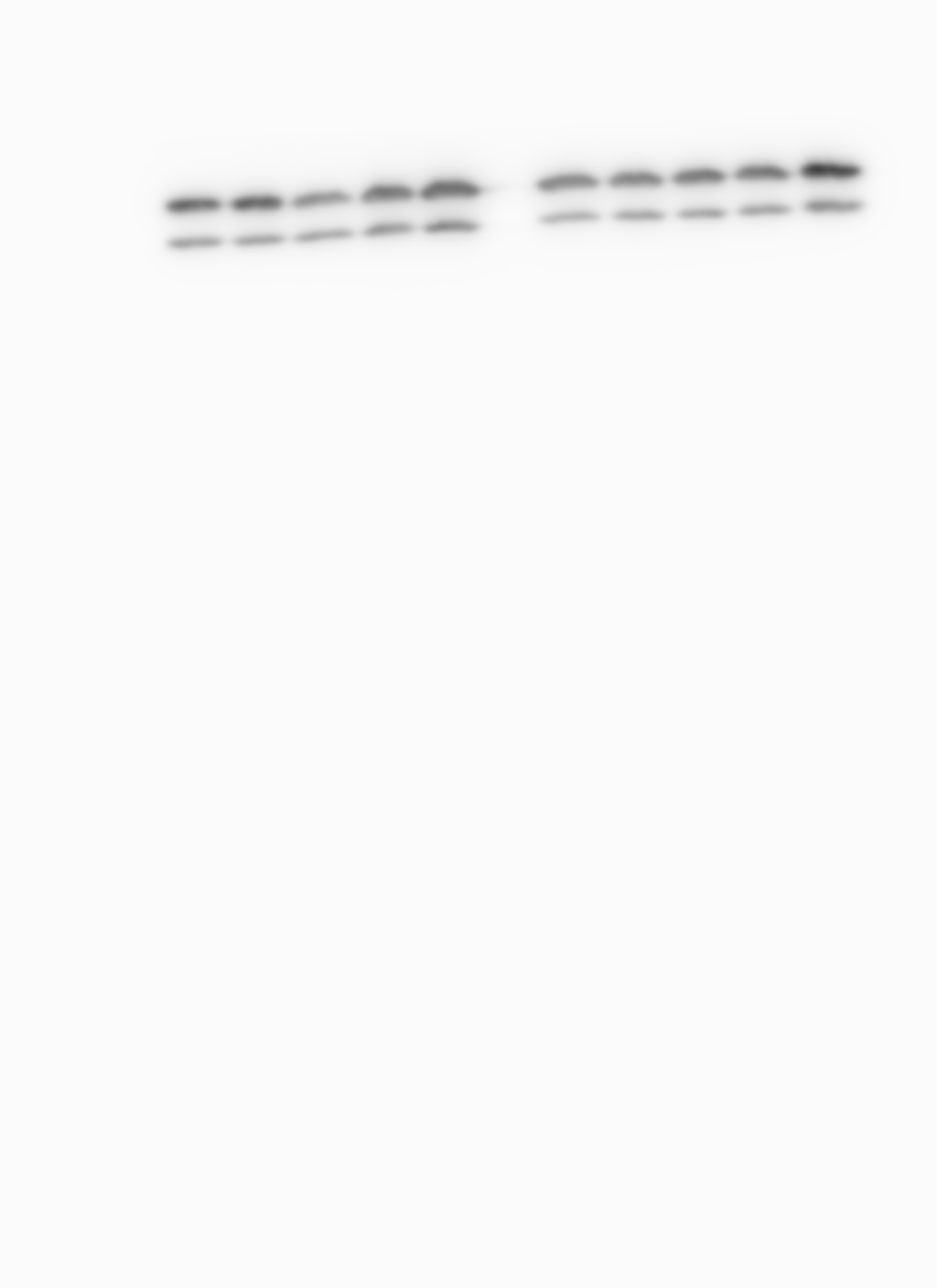

Supplement: Supplementary file 1 [file insects-16-00994-s001.zip › Figure S7/Figure 4D/2/atg8-1 2025.03.04_14.59.27_Ch/atg8-1 2025.03.04_14.59.27_Ch.tif]

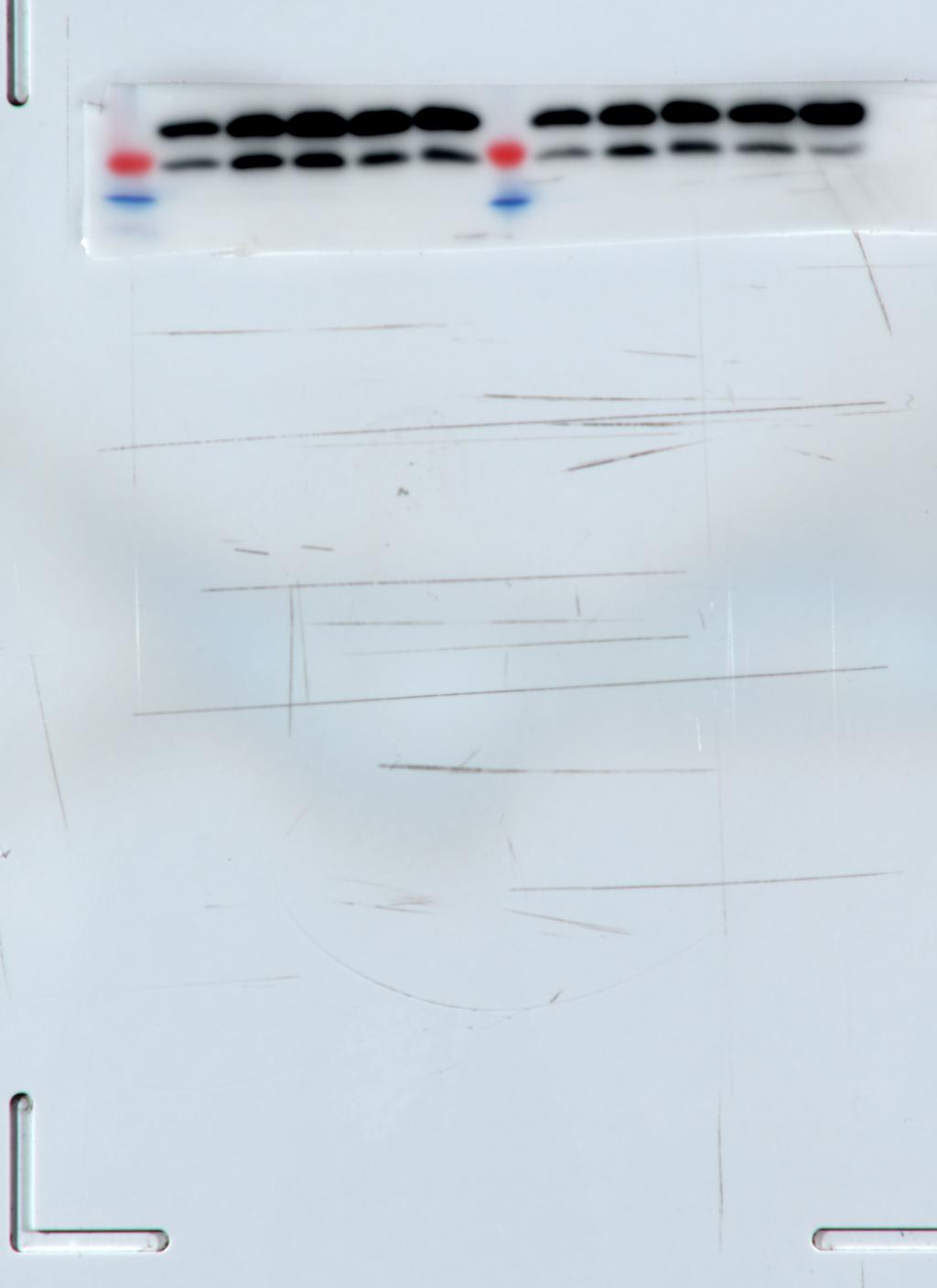

Supplement: Supplementary file 1 [file insects-16-00994-s001.zip › Figure S7/Figure 4D/2/atg8-2 2025.03.04_14.56.28_Ch/atg8-2 2025.03.04_14.56.28_Ch+Marker.jpg]

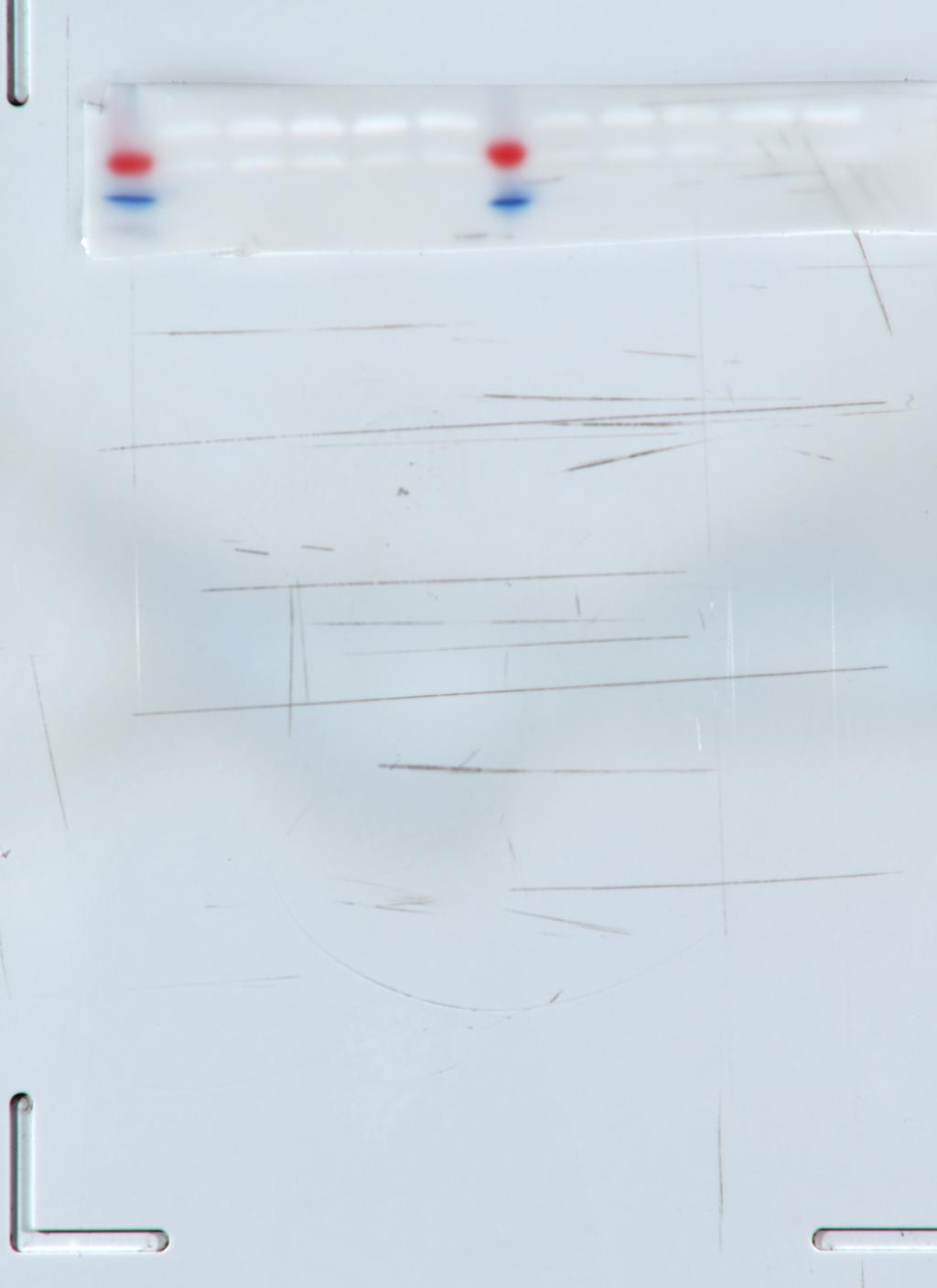

Supplement: Supplementary file 1 [file insects-16-00994-s001.zip › Figure S7/Figure 4D/2/atg8-2 2025.03.04_14.56.28_Ch/atg8-2 2025.03.04_14.56.28_Ch-Marker.jpg]

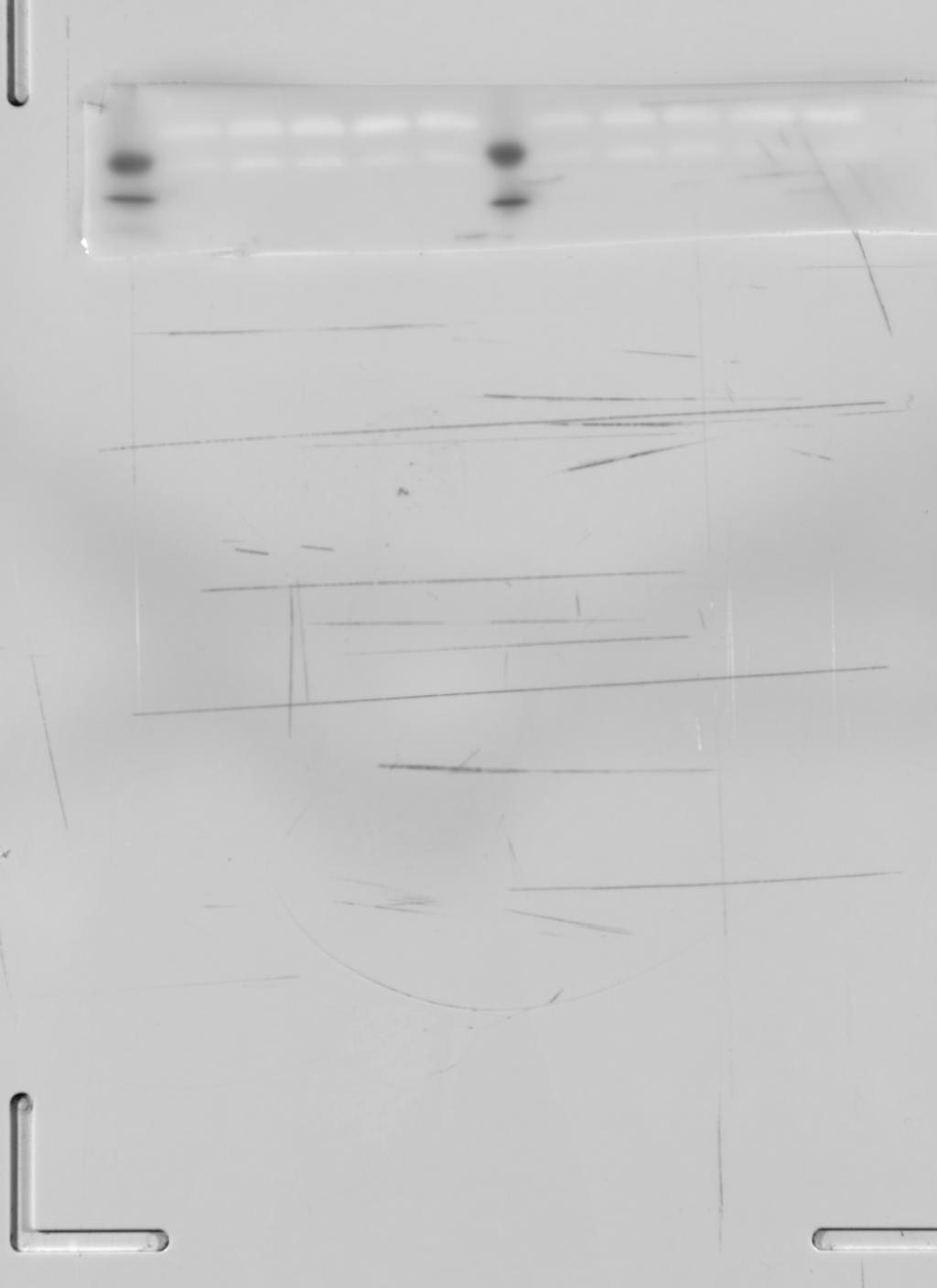

Supplement: Supplementary file 1 [file insects-16-00994-s001.zip › Figure S7/Figure 4D/2/atg8-2 2025.03.04_14.56.28_Ch/atg8-2 2025.03.04_14.56.28_Ch-Marker.tif]

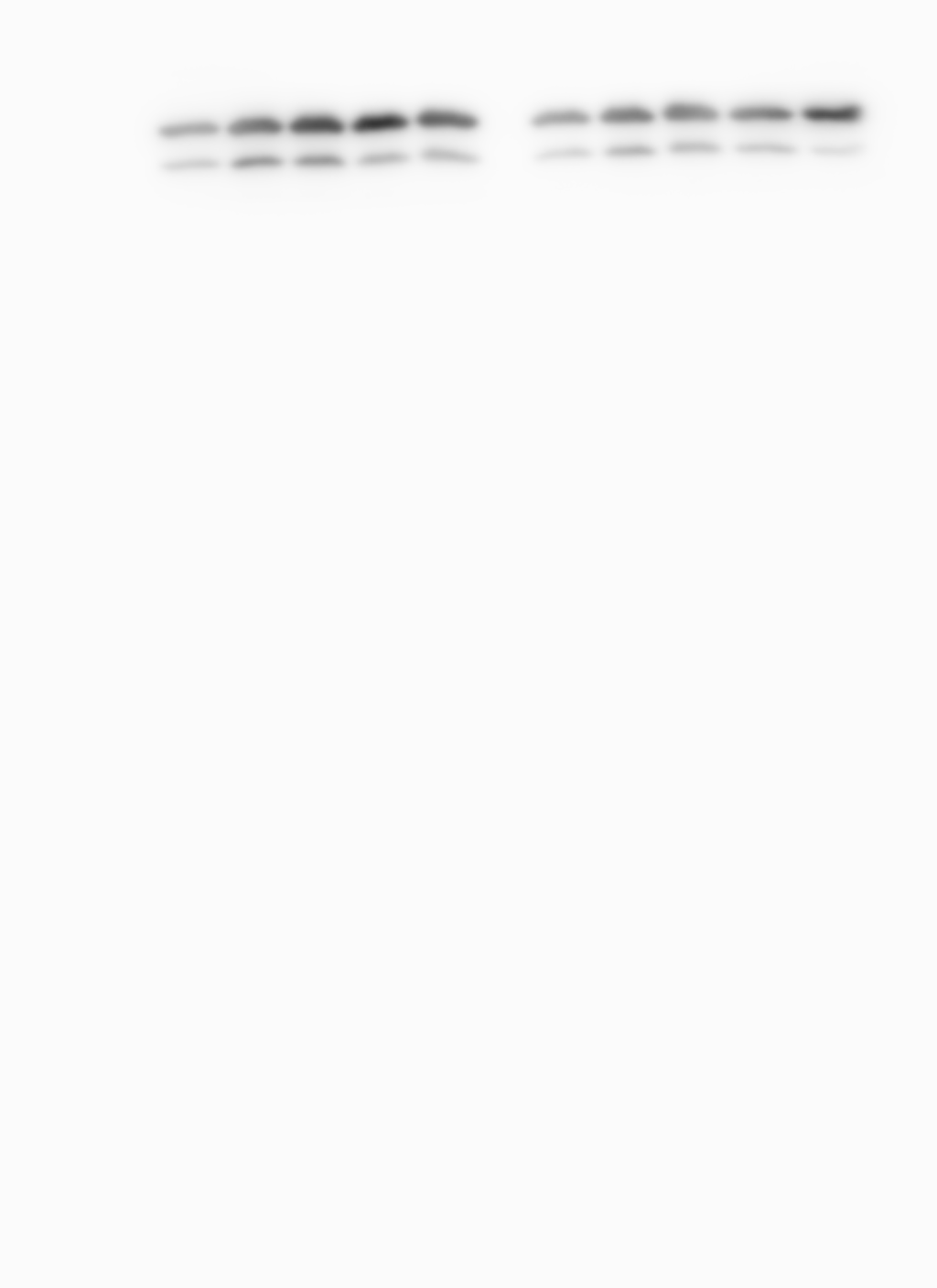

Supplement: Supplementary file 1 [file insects-16-00994-s001.zip › Figure S7/Figure 4D/2/atg8-2 2025.03.04_14.56.28_Ch/atg8-2 2025.03.04_14.56.28_Ch.tif]

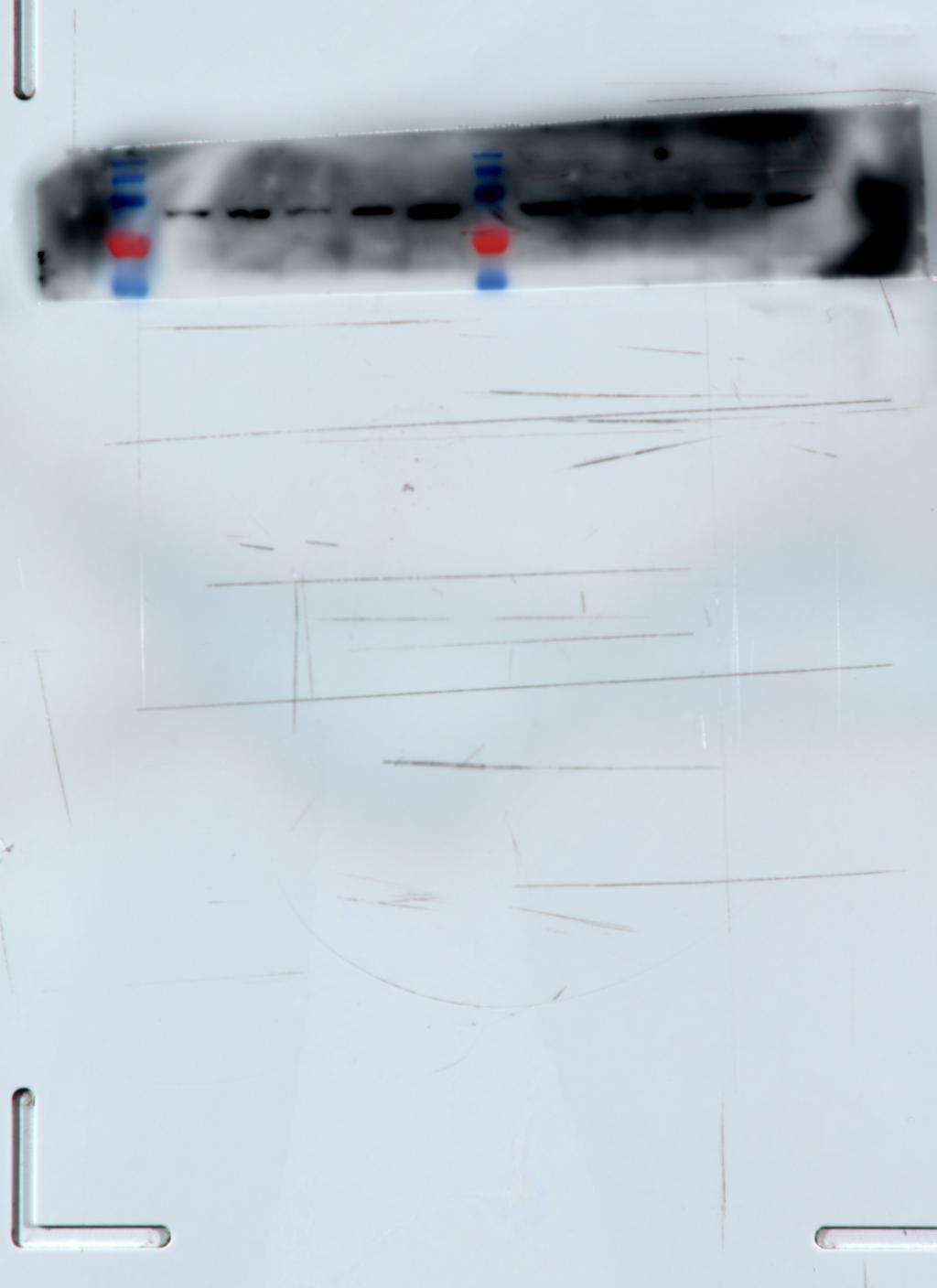

Supplement: Supplementary file 1 [file insects-16-00994-s001.zip › Figure S7/Figure 4D/2/SQSTM1-1 2025.03.04_14.41.38_Ch/p62-1 2025.03.04_14.41.38_Ch+Marker.jpg]

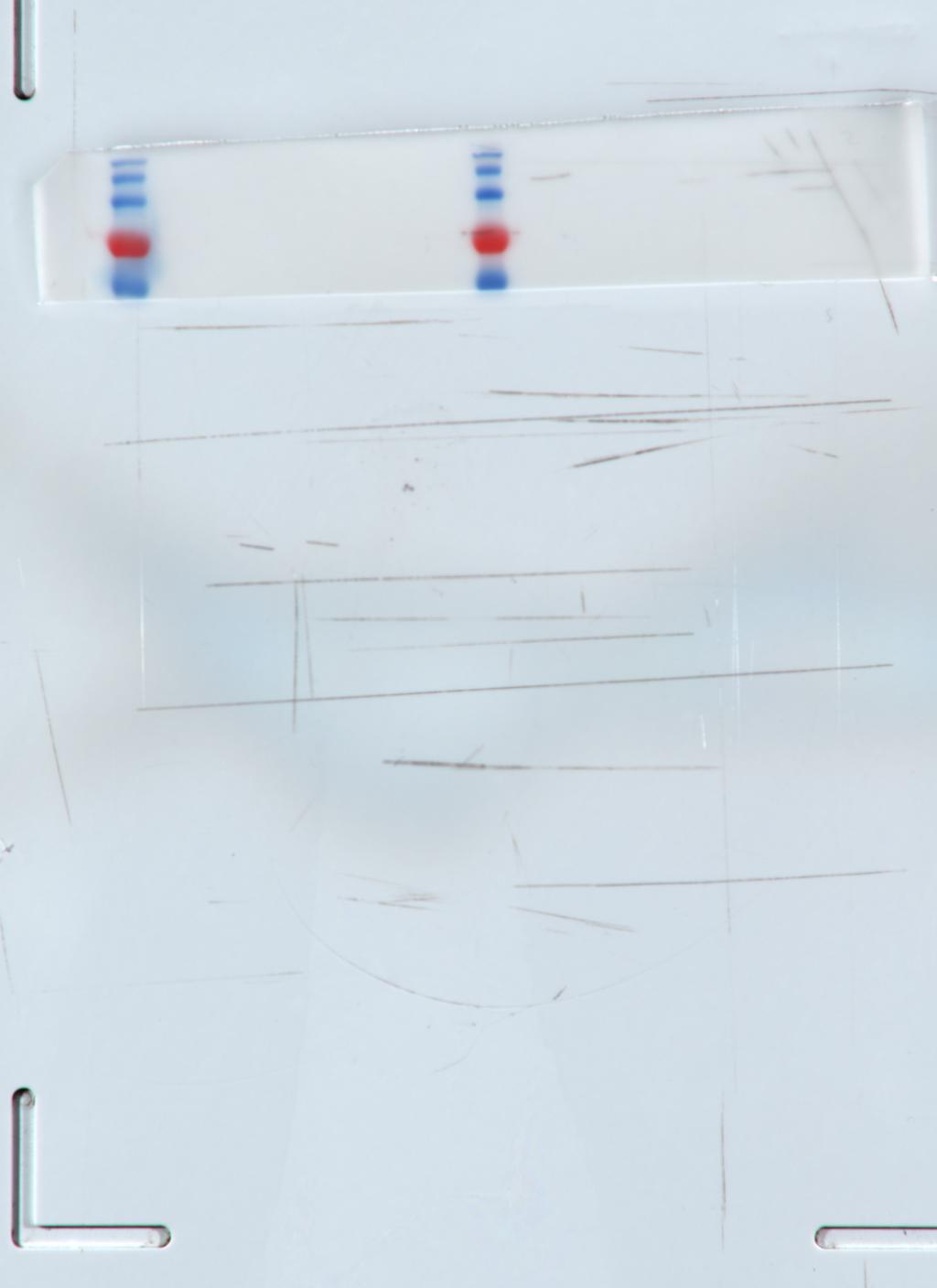

Supplement: Supplementary file 1 [file insects-16-00994-s001.zip › Figure S7/Figure 4D/2/SQSTM1-1 2025.03.04_14.41.38_Ch/p62-1 2025.03.04_14.41.38_Ch-Marker.jpg]

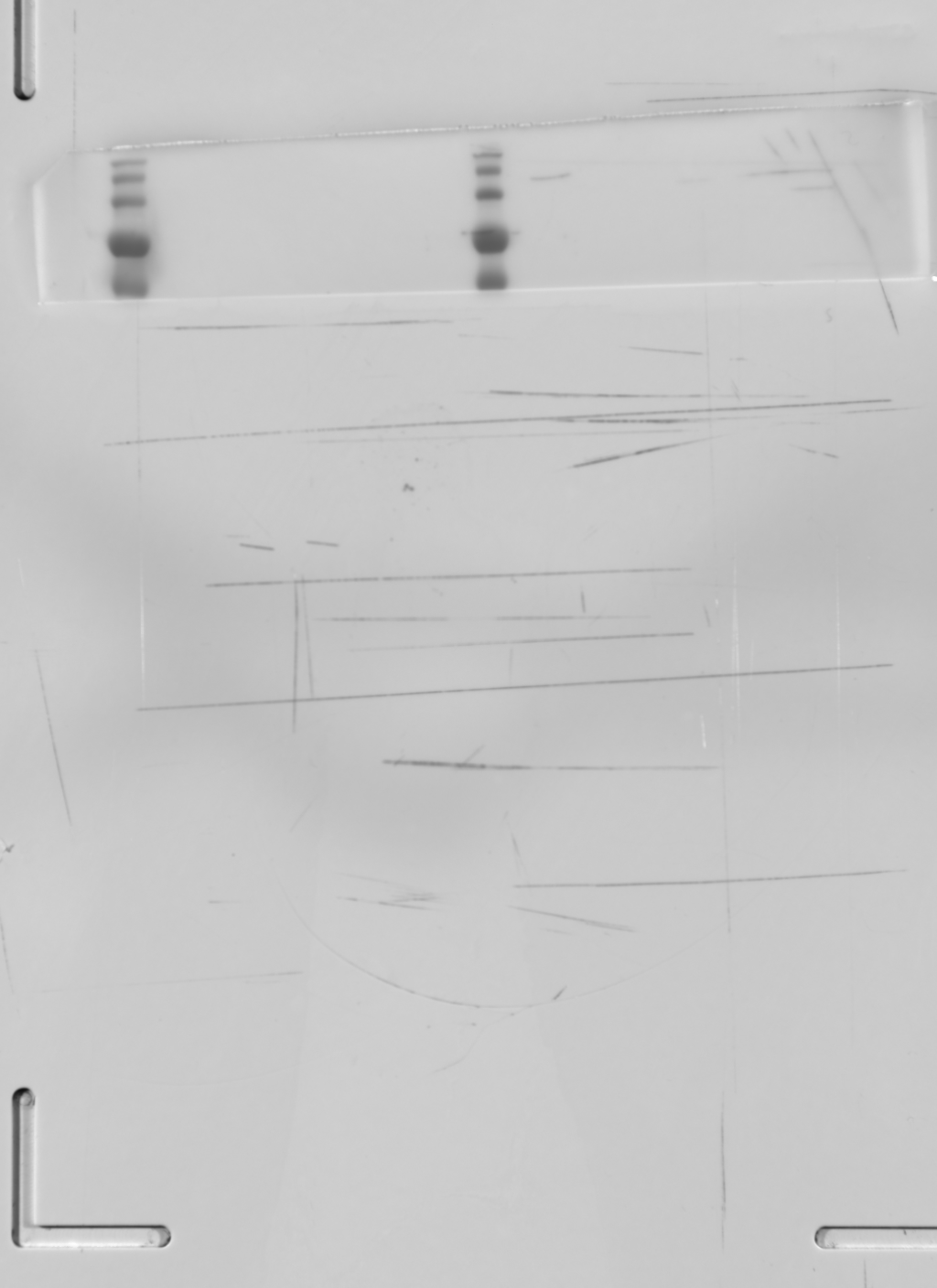

Supplement: Supplementary file 1 [file insects-16-00994-s001.zip › Figure S7/Figure 4D/2/SQSTM1-1 2025.03.04_14.41.38_Ch/p62-1 2025.03.04_14.41.38_Ch-Marker.tif]

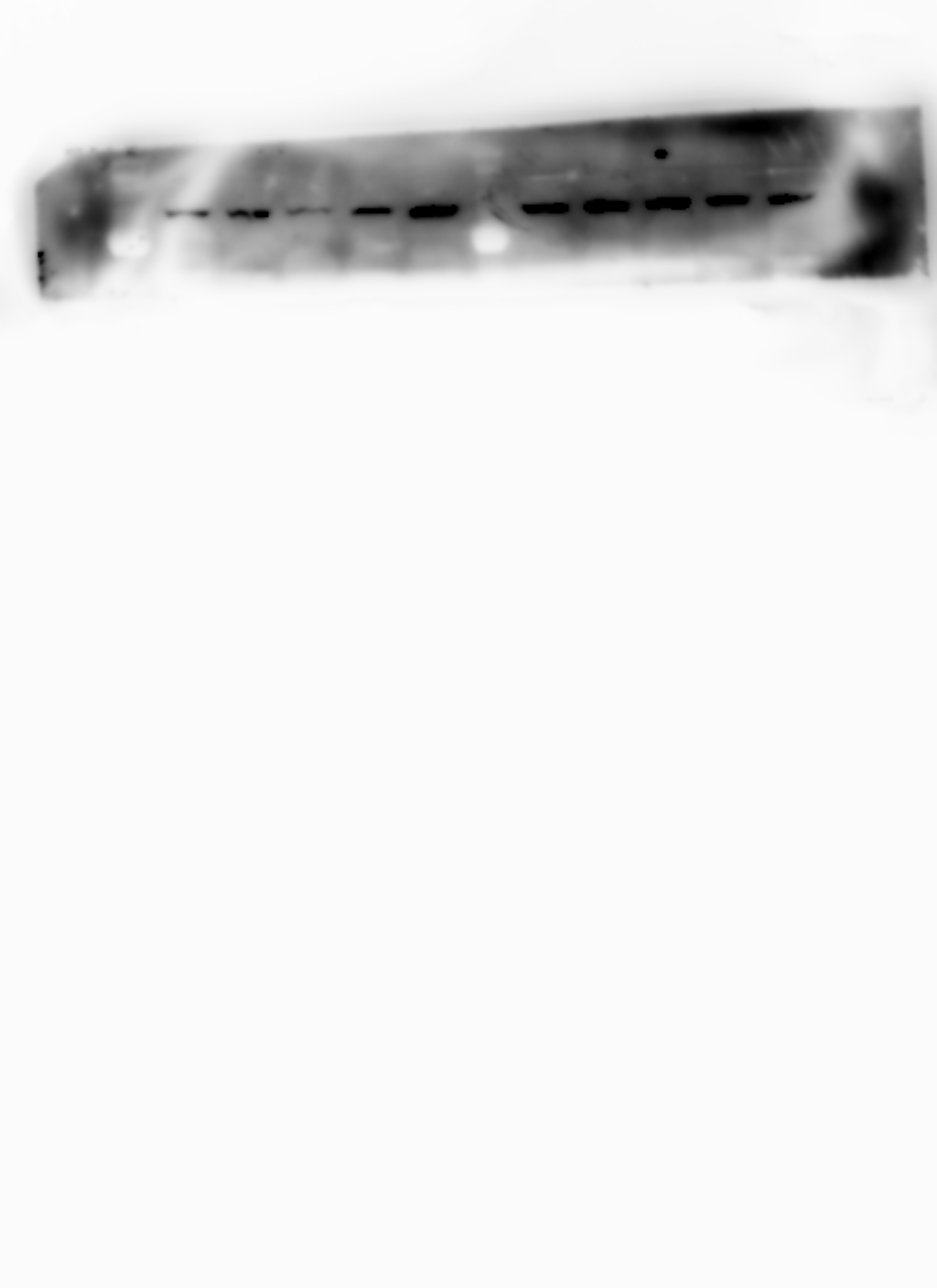

Supplement: Supplementary file 1 [file insects-16-00994-s001.zip › Figure S7/Figure 4D/2/SQSTM1-1 2025.03.04_14.41.38_Ch/p62-1 2025.03.04_14.41.38_Ch.tif]

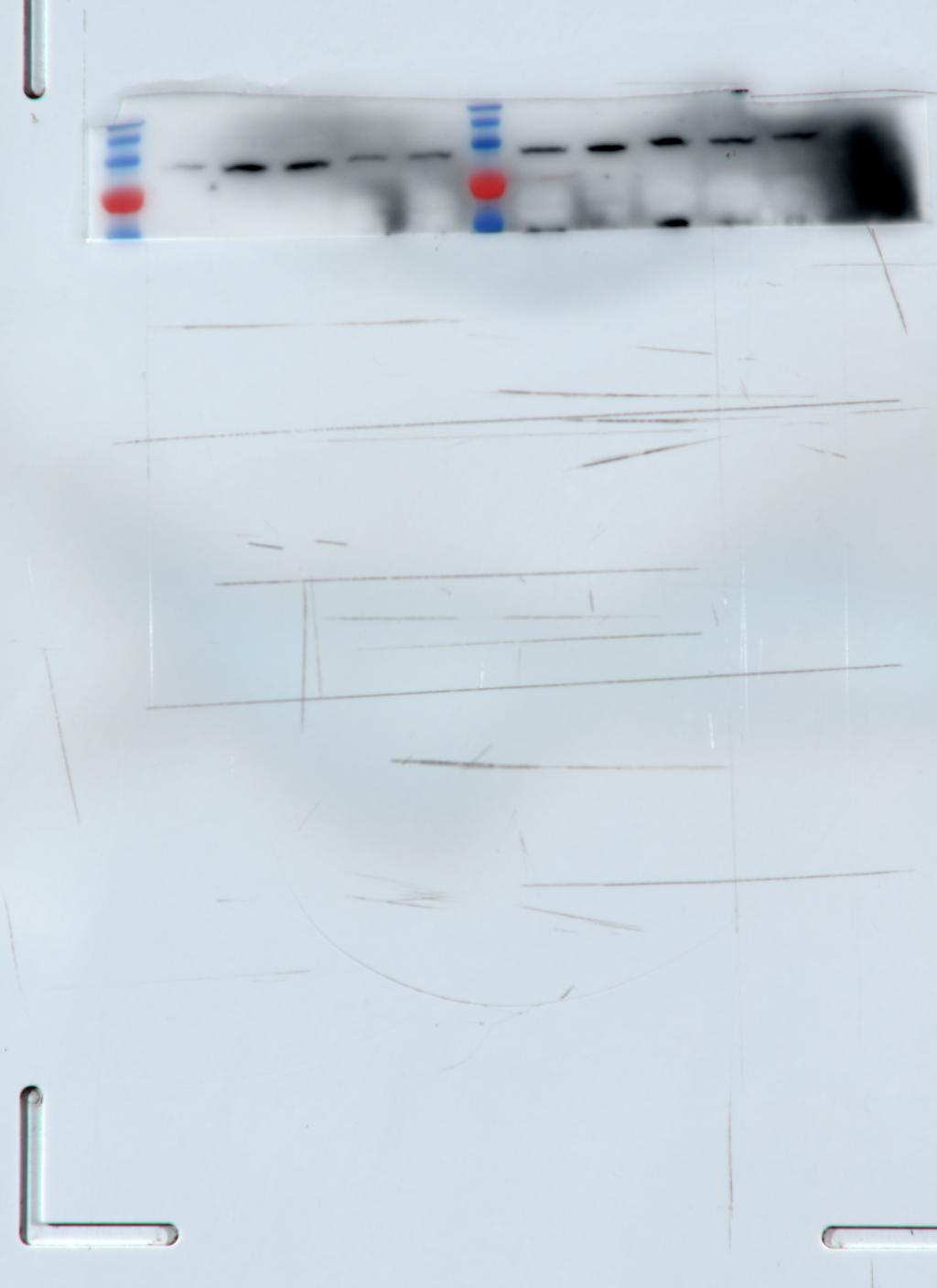

Supplement: Supplementary file 1 [file insects-16-00994-s001.zip › Figure S7/Figure 4D/2/SQSTM1-2 2025.03.04_14.31.26_Ch/p62-2 2025.03.04_14.31.26_Ch+Marker.jpg]

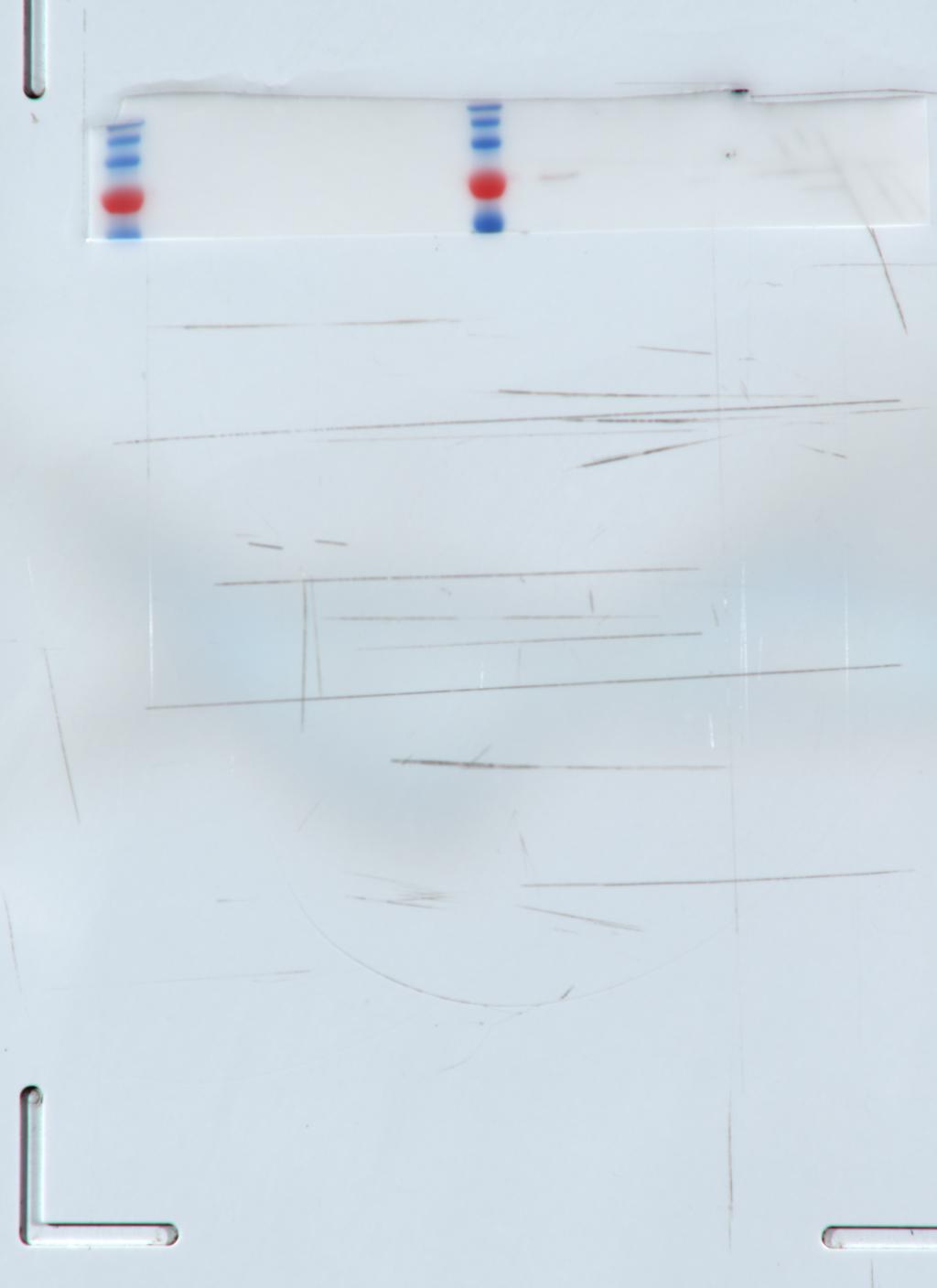

Supplement: Supplementary file 1 [file insects-16-00994-s001.zip › Figure S7/Figure 4D/2/SQSTM1-2 2025.03.04_14.31.26_Ch/p62-2 2025.03.04_14.31.26_Ch-Marker.jpg]

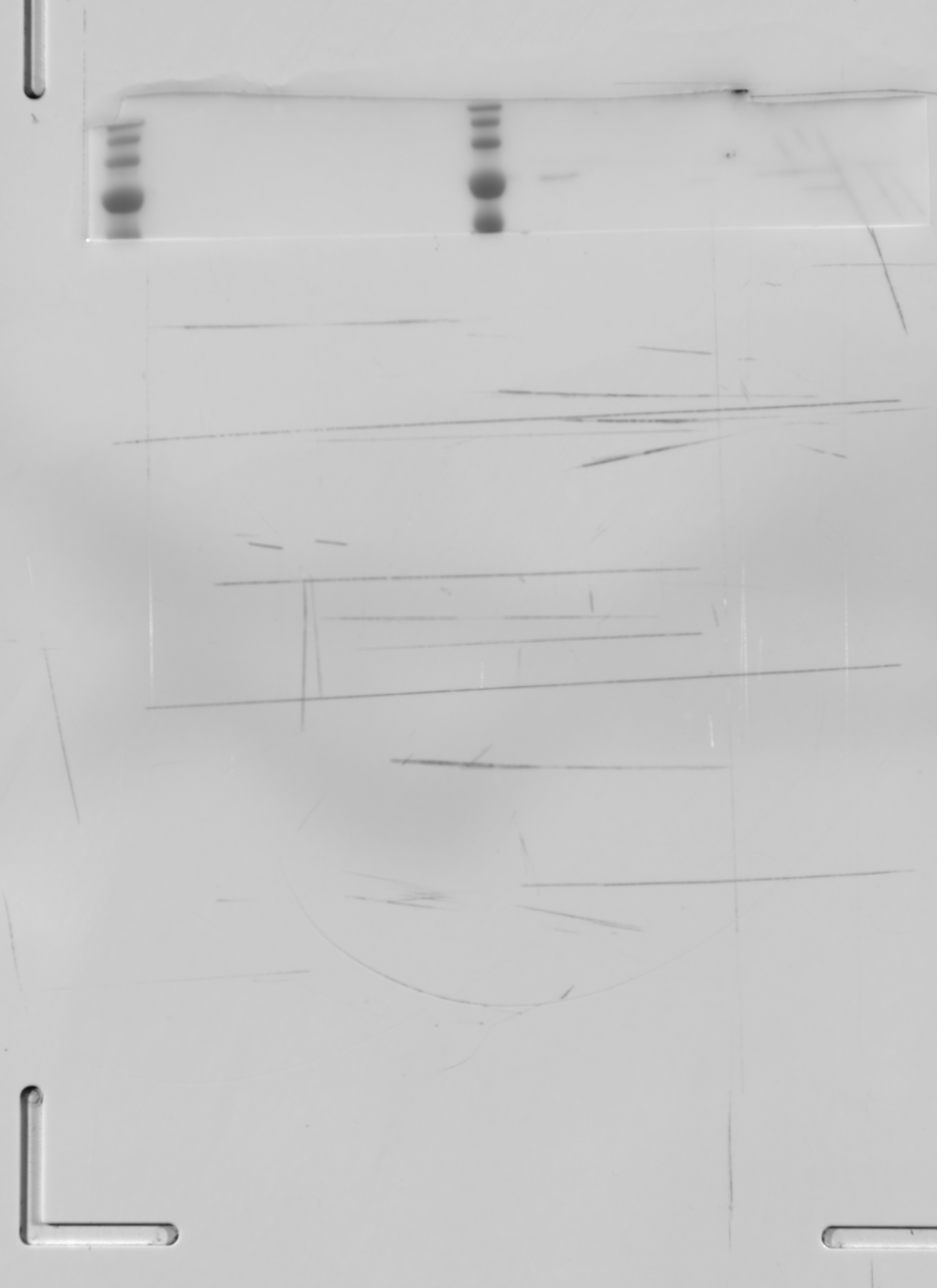

Supplement: Supplementary file 1 [file insects-16-00994-s001.zip › Figure S7/Figure 4D/2/SQSTM1-2 2025.03.04_14.31.26_Ch/p62-2 2025.03.04_14.31.26_Ch-Marker.tif]

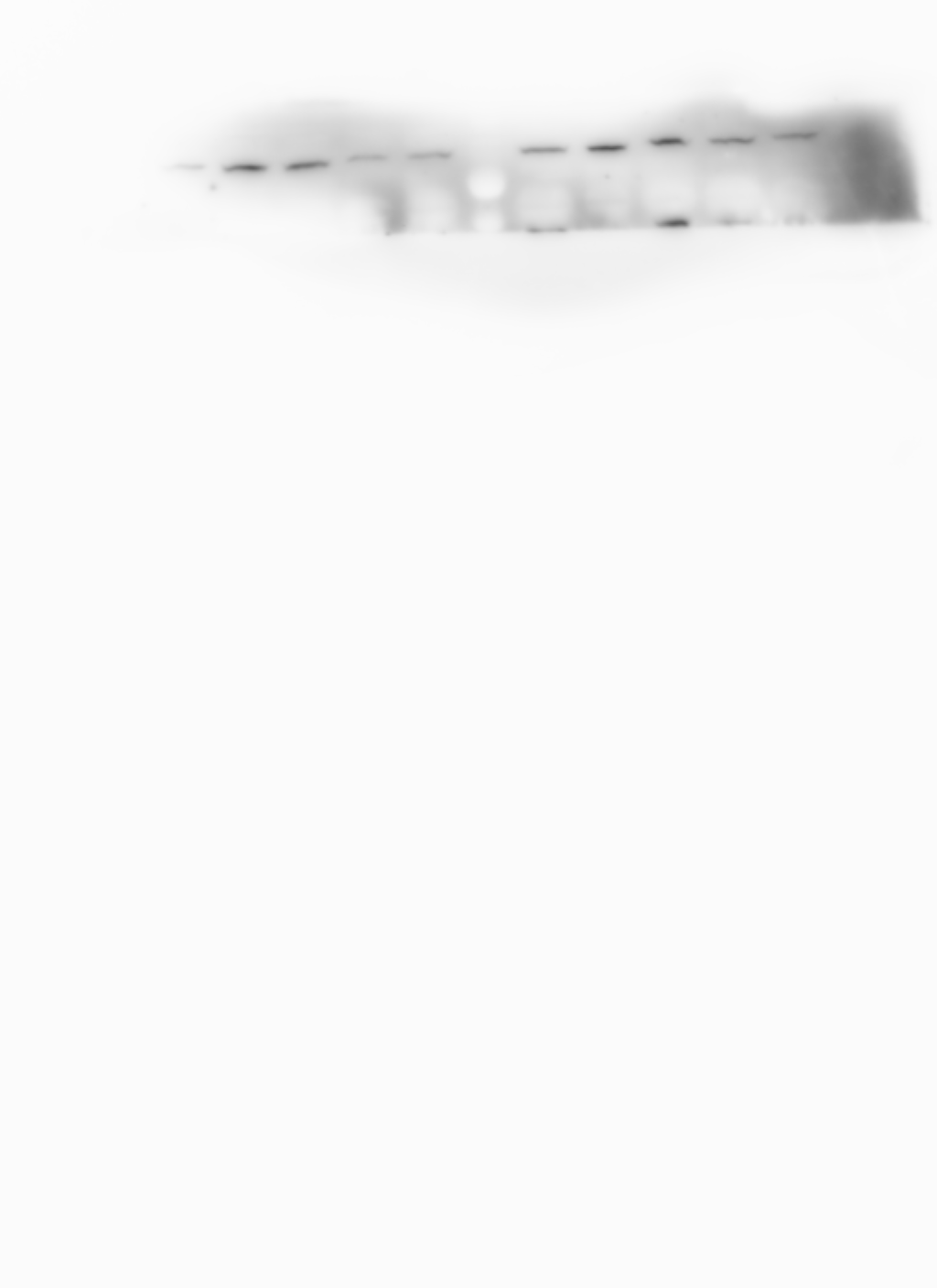

Supplement: Supplementary file 1 [file insects-16-00994-s001.zip › Figure S7/Figure 4D/2/SQSTM1-2 2025.03.04_14.31.26_Ch/p62-2 2025.03.04_14.31.26_Ch.tif]

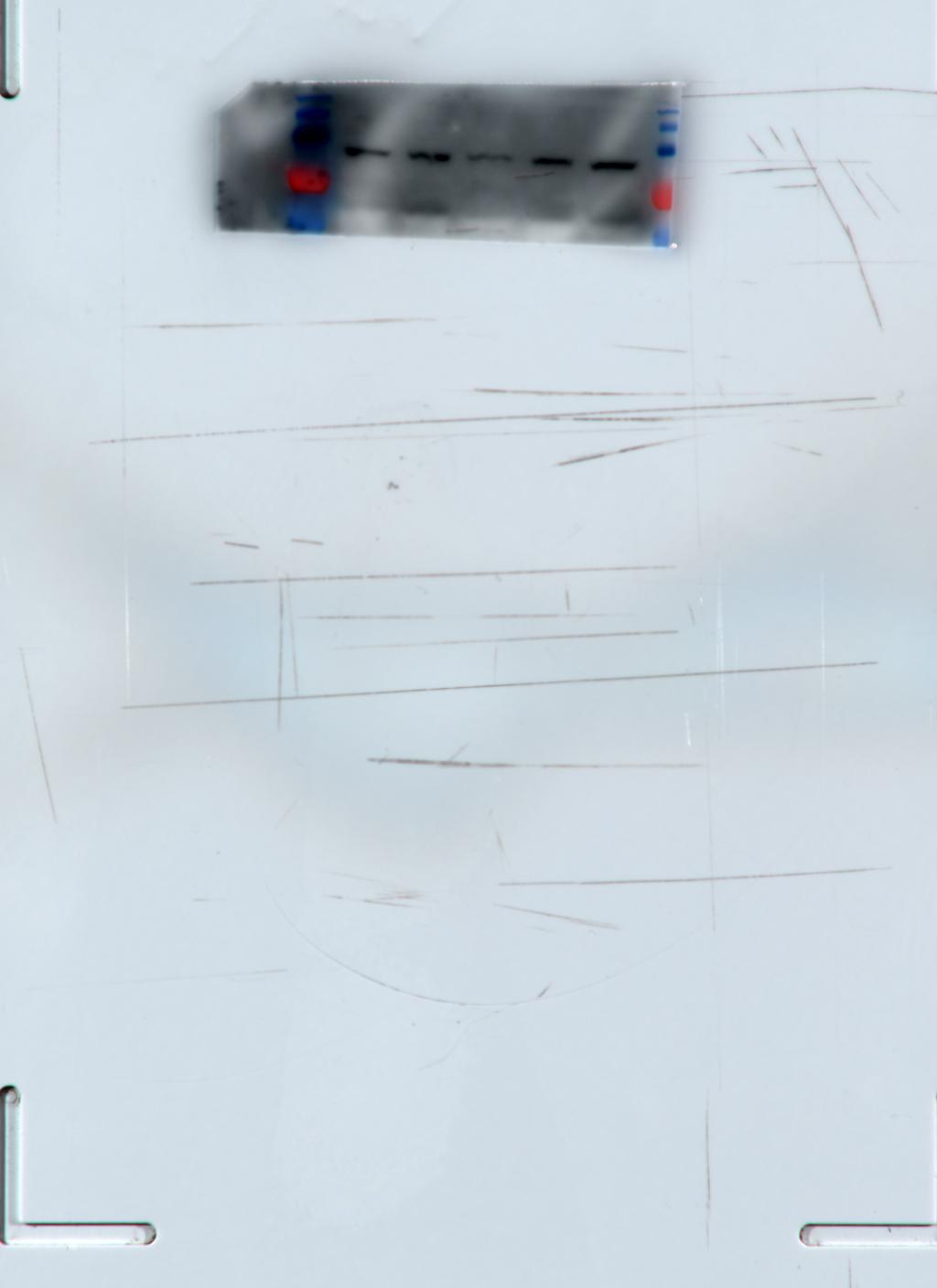

Supplement: Supplementary file 1 [file insects-16-00994-s001.zip › Figure S7/Figure 4D/2/SQSTM1.1 2025.03.04_14.52.12_Ch/p62-1.1 2025.03.04_14.52.12_Ch+Marker.jpg]

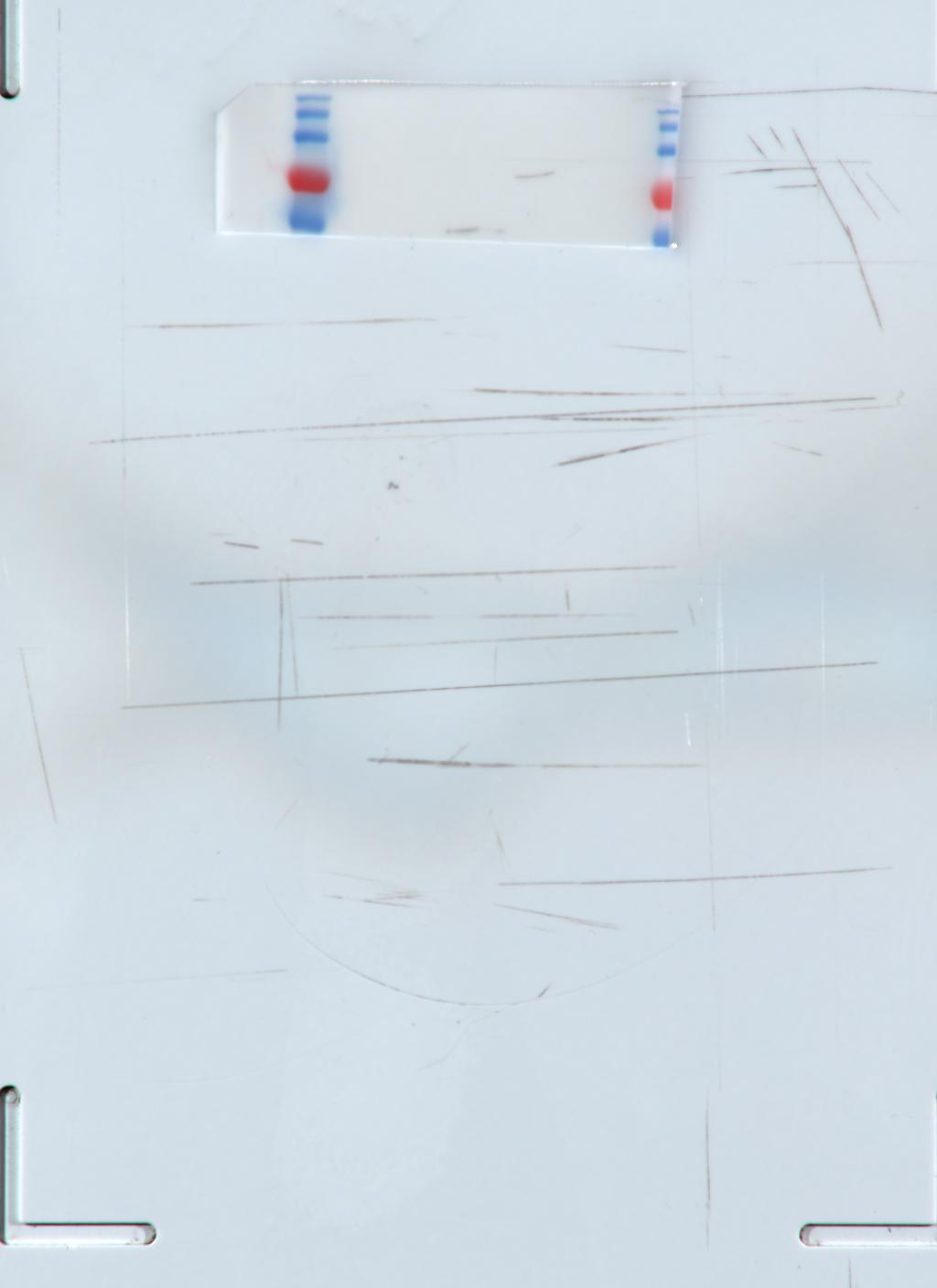

Supplement: Supplementary file 1 [file insects-16-00994-s001.zip › Figure S7/Figure 4D/2/SQSTM1.1 2025.03.04_14.52.12_Ch/p62-1.1 2025.03.04_14.52.12_Ch-Marker.jpg]

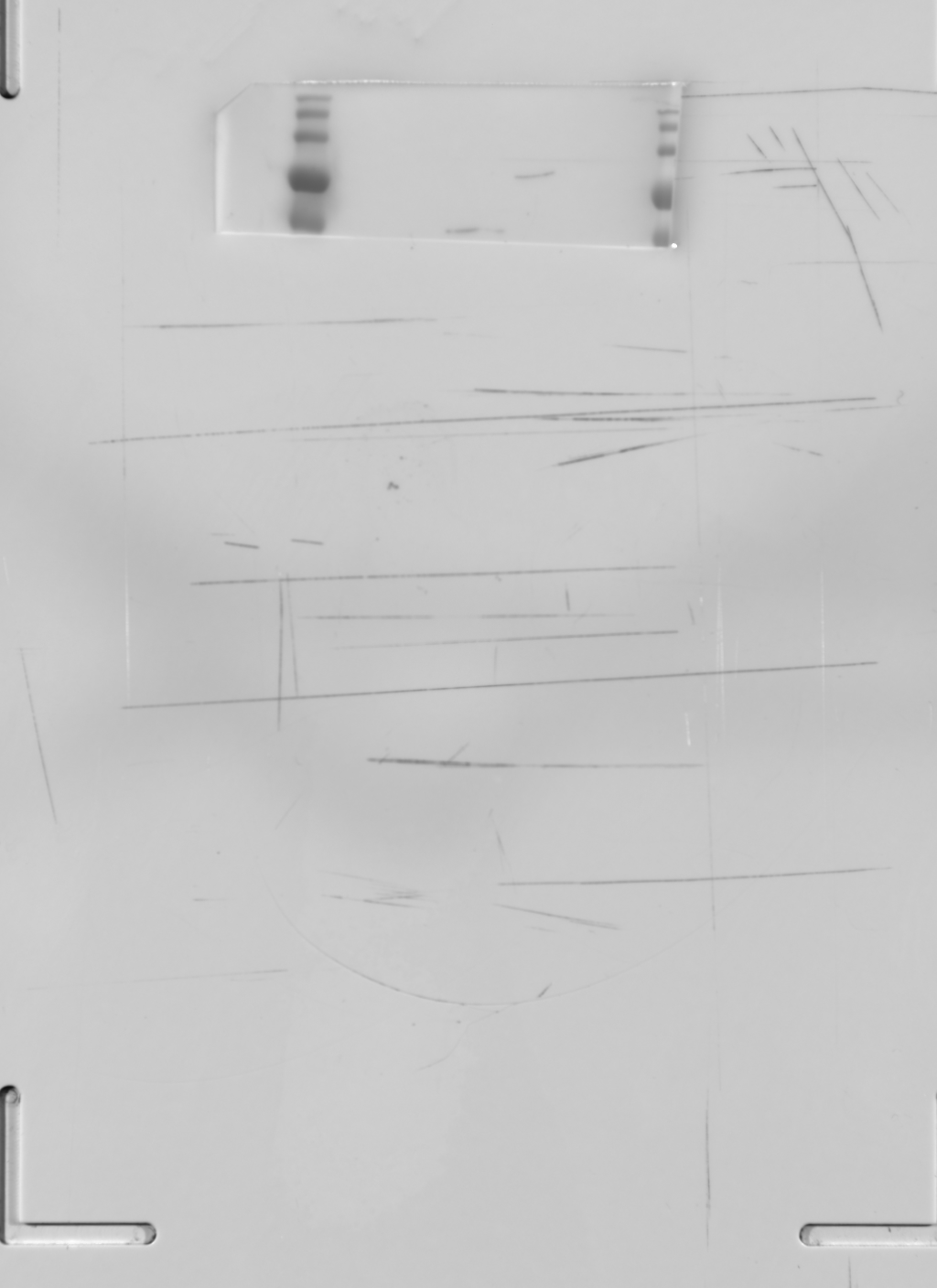

Supplement: Supplementary file 1 [file insects-16-00994-s001.zip › Figure S7/Figure 4D/2/SQSTM1.1 2025.03.04_14.52.12_Ch/p62-1.1 2025.03.04_14.52.12_Ch-Marker.tif]

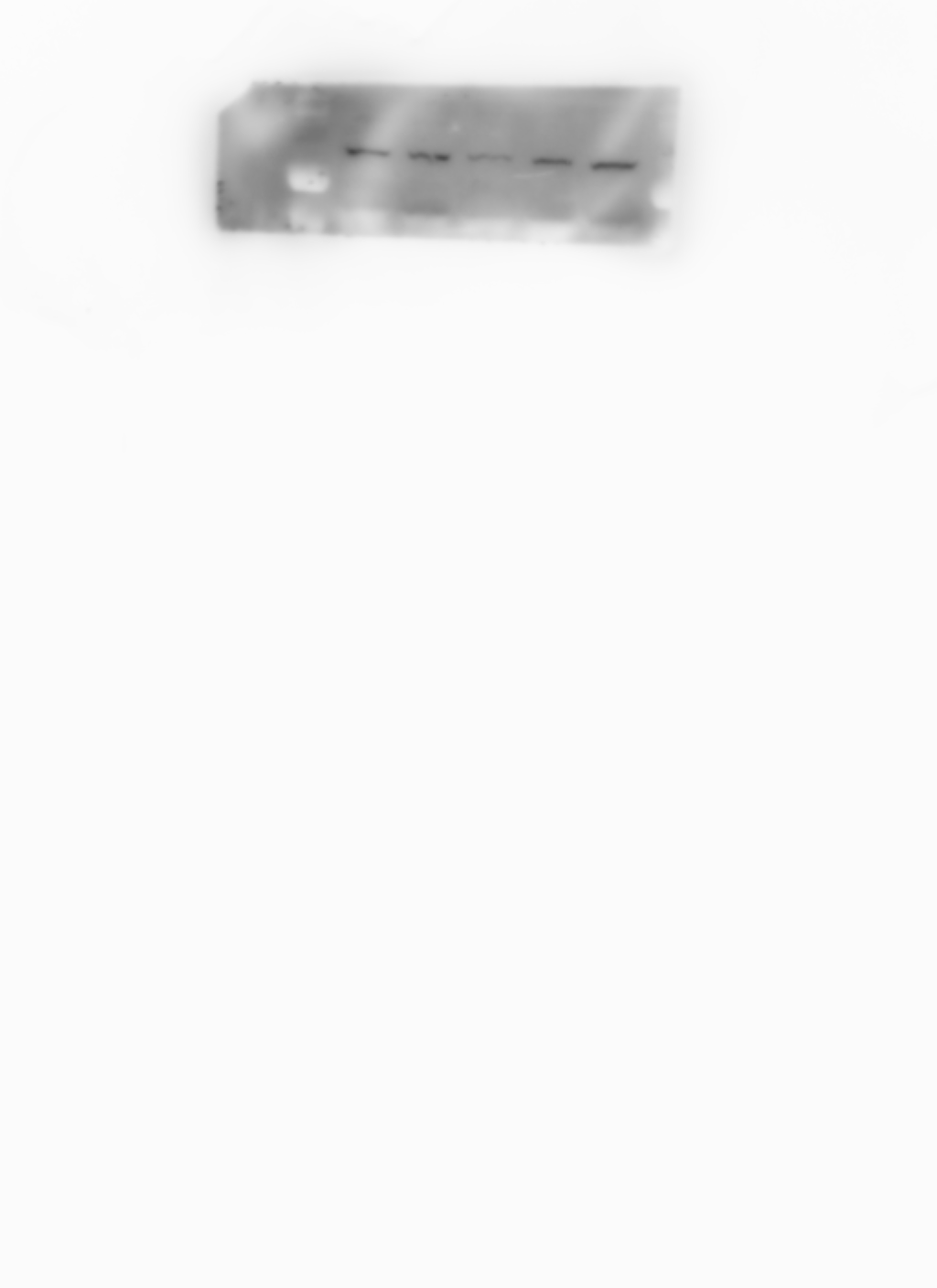

Supplement: Supplementary file 1 [file insects-16-00994-s001.zip › Figure S7/Figure 4D/2/SQSTM1.1 2025.03.04_14.52.12_Ch/p62-1.1 2025.03.04_14.52.12_Ch.tif]

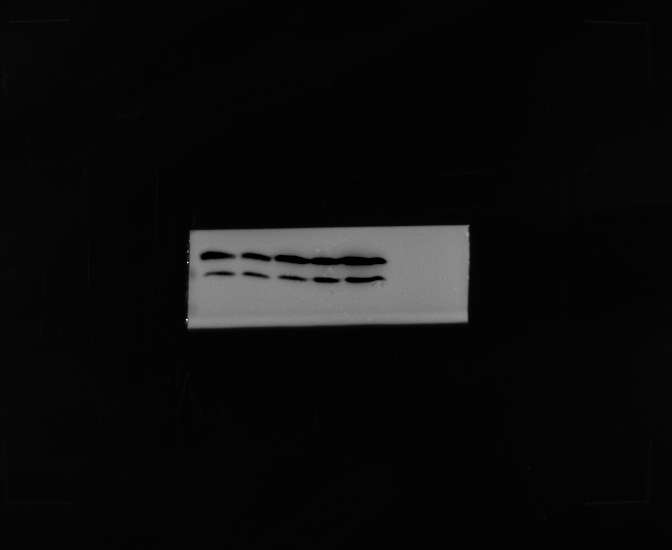

Supplement: Supplementary file 1 [file insects-16-00994-s001.zip › Figure S7/Figure 4D/3/atg8-2.tif]

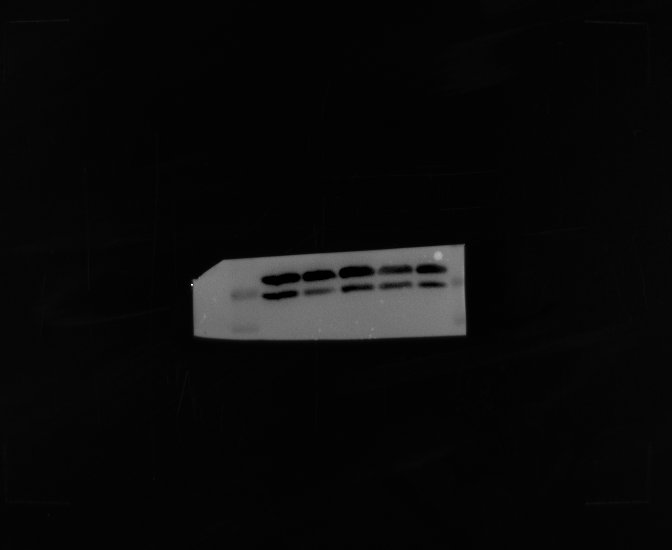

Supplement: Supplementary file 1 [file insects-16-00994-s001.zip › Figure S7/Figure 4D/3/atg8-4.tif]

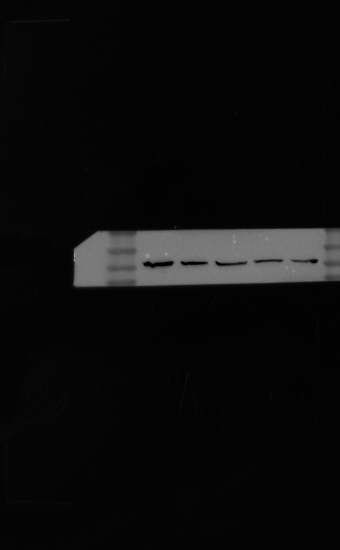

Supplement: Supplementary file 1 [file insects-16-00994-s001.zip › Figure S7/Figure 4D/3/MOCK-actin-1.tif]

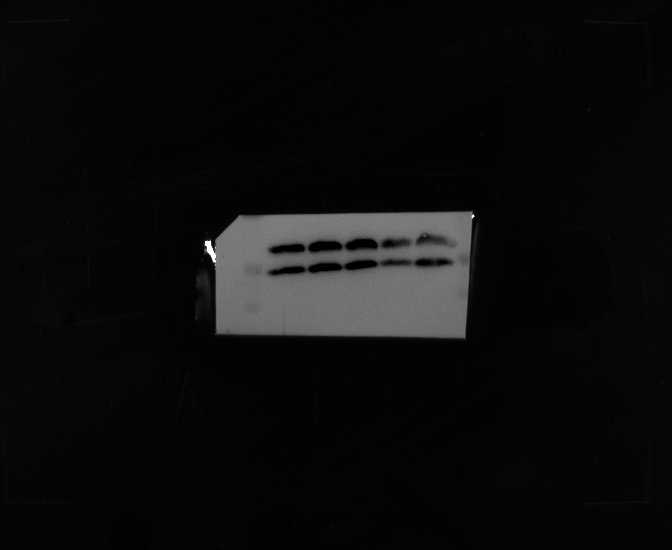

Supplement: Supplementary file 1 [file insects-16-00994-s001.zip › Figure S7/Figure 4D/3/mock-atg8-1.tif]

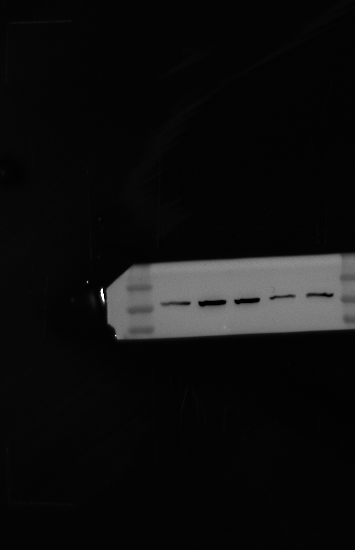

Supplement: Supplementary file 1 [file insects-16-00994-s001.zip › Figure S7/Figure 4D/3/rapa+bafa-actin-2.tif]

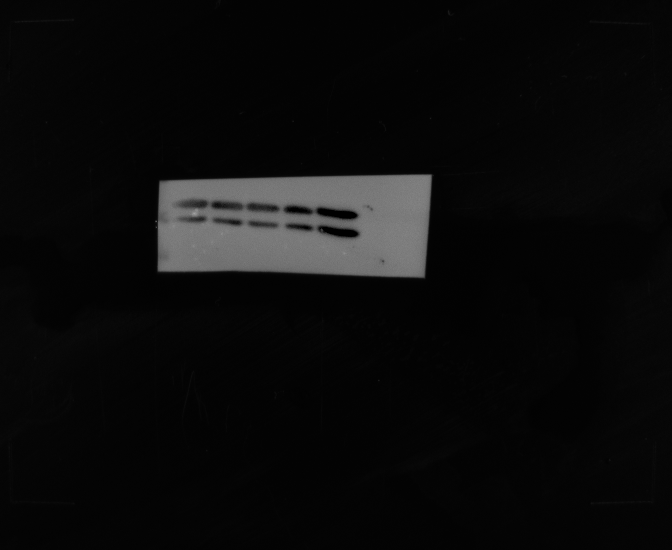

Supplement: Supplementary file 1 [file insects-16-00994-s001.zip › Figure S7/Figure 4D/3/RAPA+bafa-atg8-3.tif]

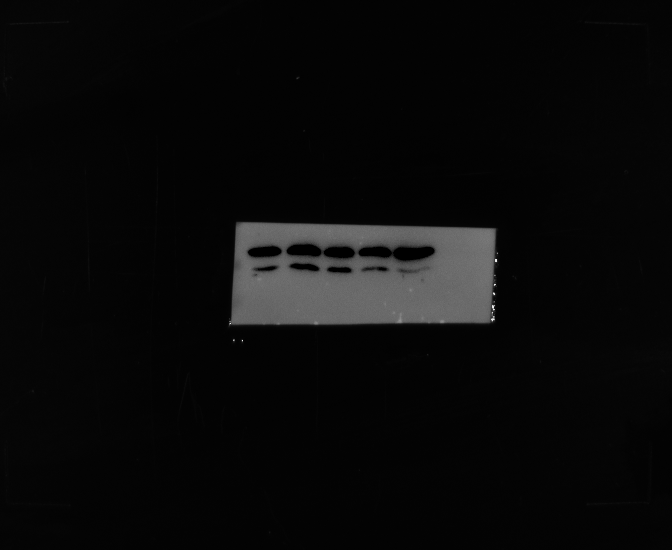

Supplement: Supplementary file 1 [file insects-16-00994-s001.zip › Figure S7/Figure 4D/3/rapa+MG132-atg8-4(1).tif]

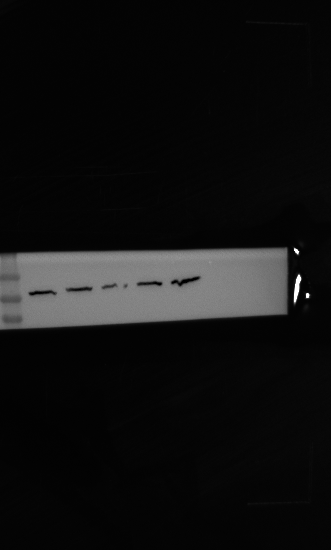

Supplement: Supplementary file 1 [file insects-16-00994-s001.zip › Figure S7/Figure 4D/3/rapa+MG132actin-2.tif]

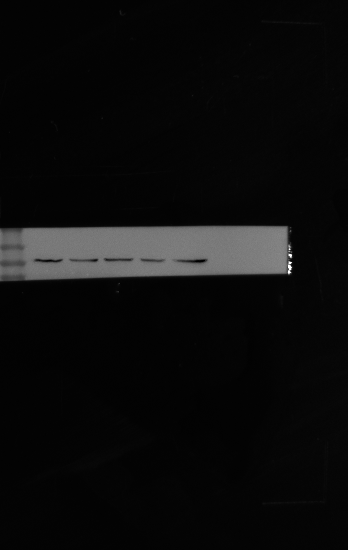

Supplement: Supplementary file 1 [file insects-16-00994-s001.zip › Figure S7/Figure 4D/3/RAPA-actin-1.tif]

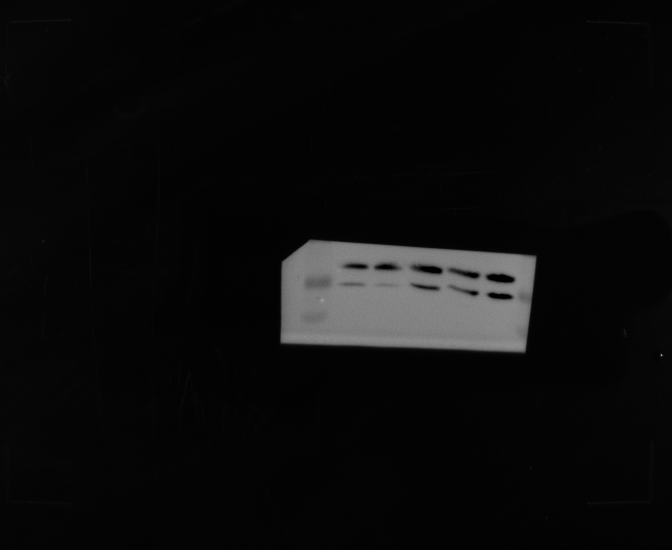

Supplement: Supplementary file 1 [file insects-16-00994-s001.zip › Figure S7/Figure 4D/3/RAPA-atg8-2.tif]

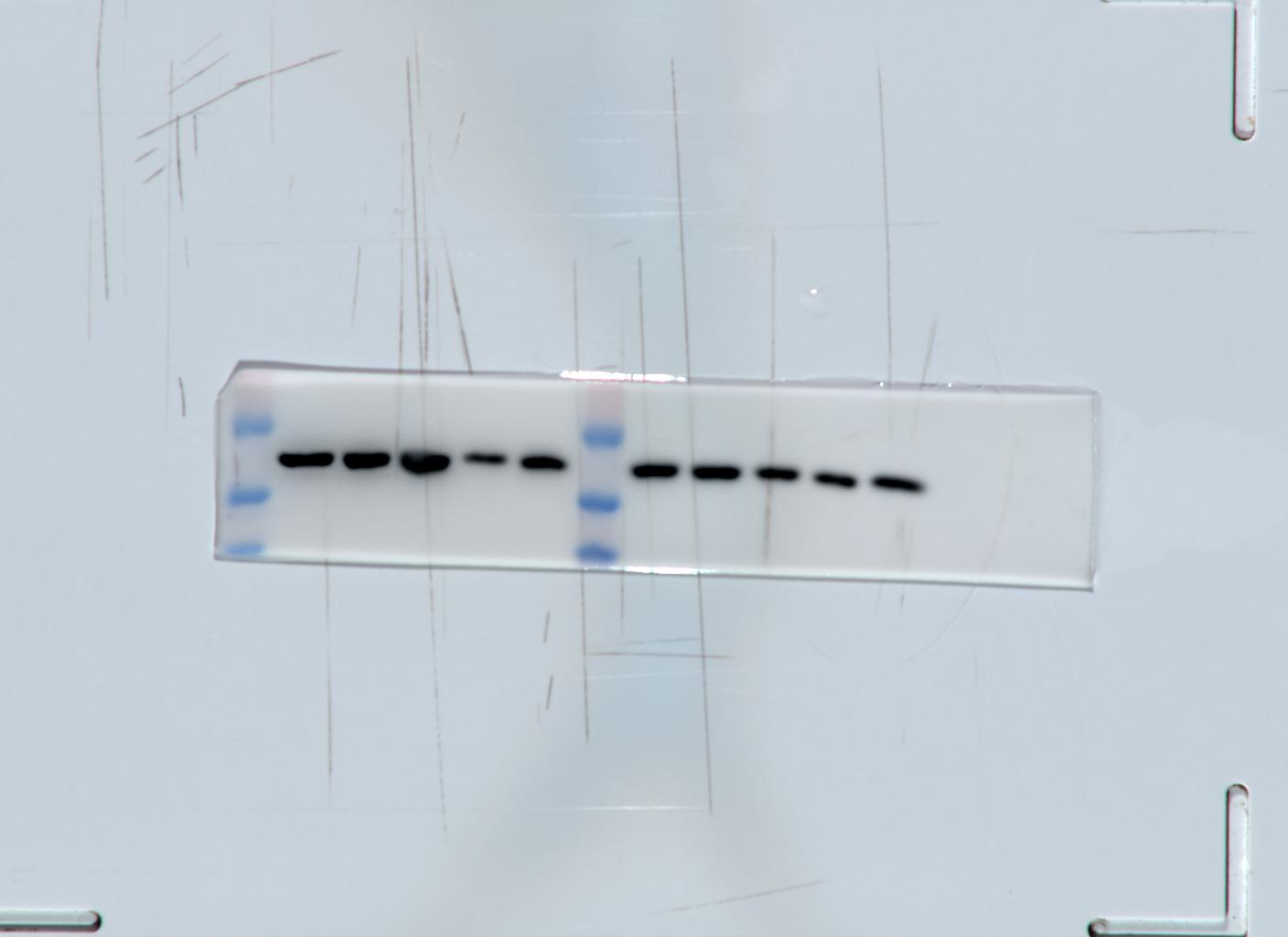

Supplement: Supplementary file 1 [file insects-16-00994-s001.zip › Figure S7/Figure 4D/3/SQSTM1/actin-1-2 2024.12.16_18.09.05_Ch/actin-1 2024.12.16_18.09.05_Ch+Marker.jpg]

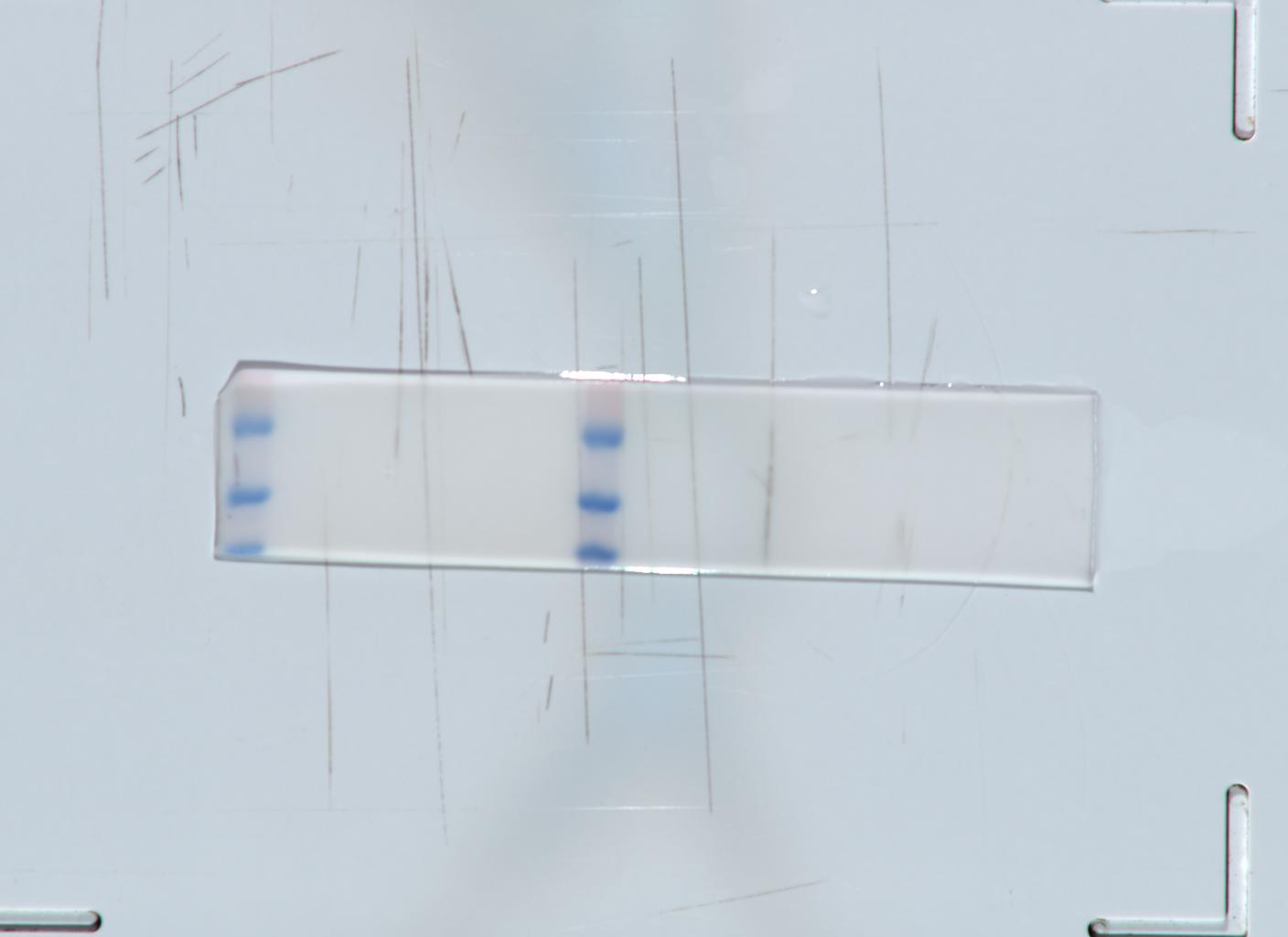

Supplement: Supplementary file 1 [file insects-16-00994-s001.zip › Figure S7/Figure 4D/3/SQSTM1/actin-1-2 2024.12.16_18.09.05_Ch/actin-1 2024.12.16_18.09.05_Ch-Marker.jpg]

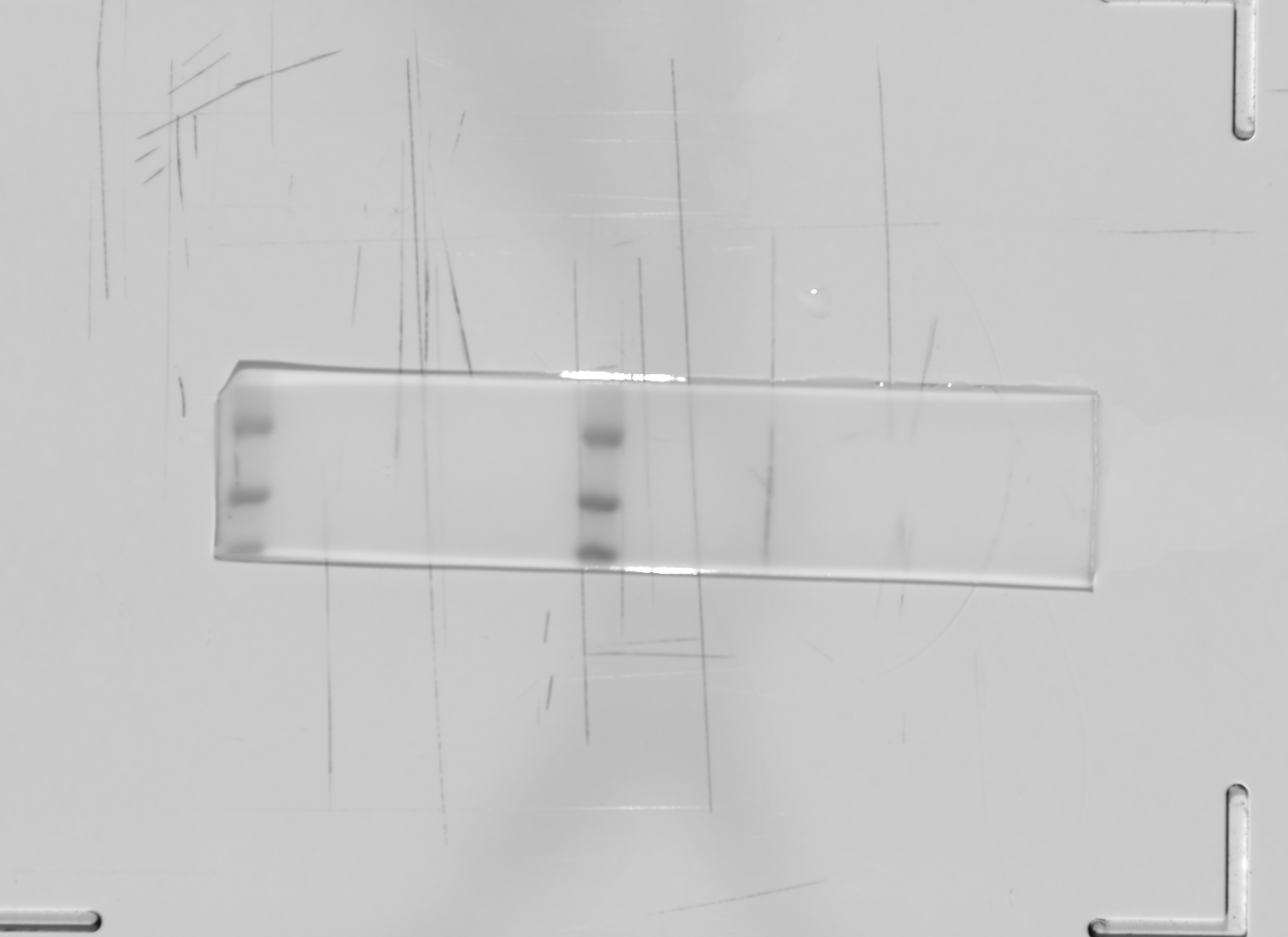

Supplement: Supplementary file 1 [file insects-16-00994-s001.zip › Figure S7/Figure 4D/3/SQSTM1/actin-1-2 2024.12.16_18.09.05_Ch/actin-1 2024.12.16_18.09.05_Ch-Marker.tif]

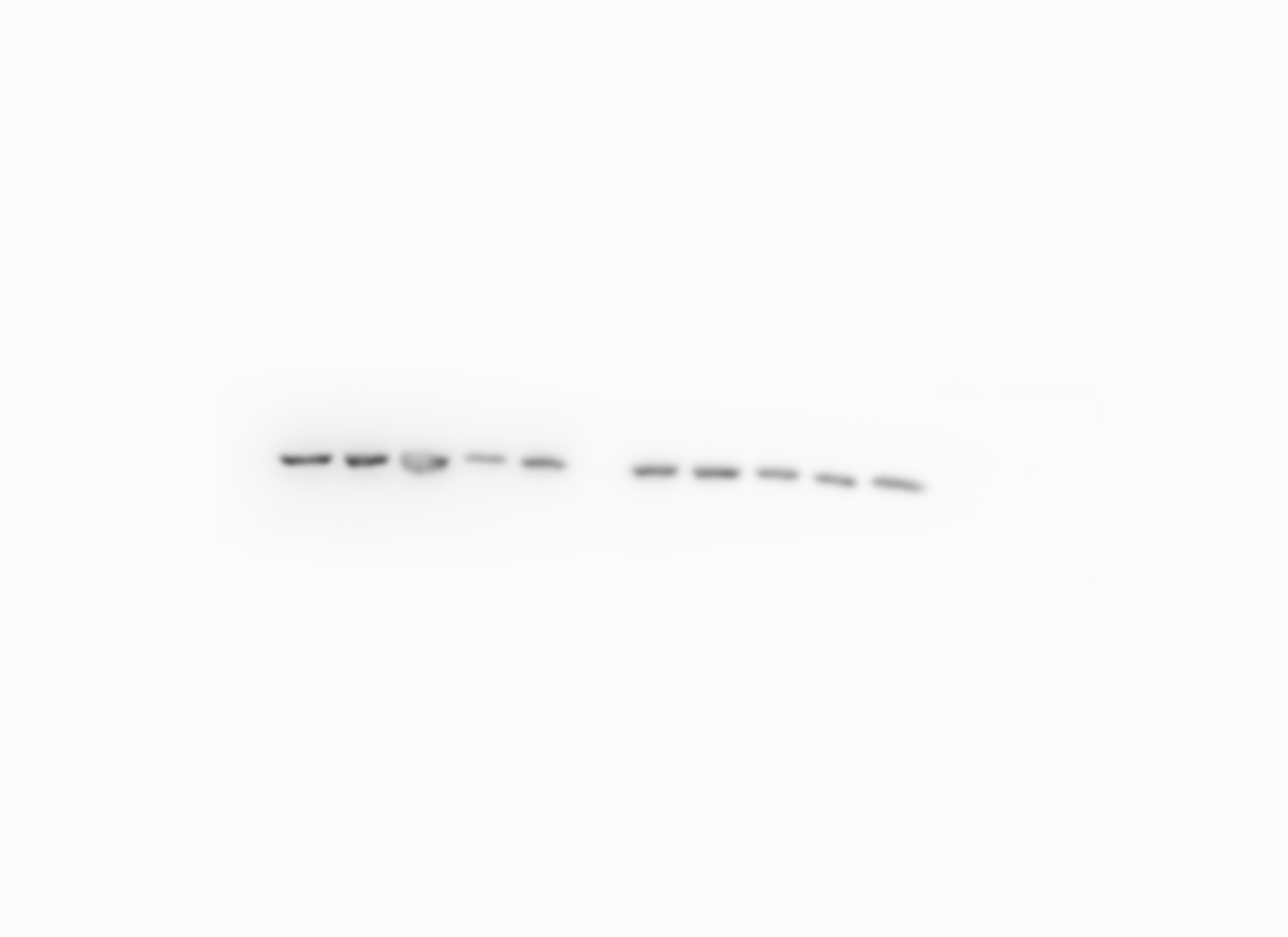

Supplement: Supplementary file 1 [file insects-16-00994-s001.zip › Figure S7/Figure 4D/3/SQSTM1/actin-1-2 2024.12.16_18.09.05_Ch/actin-1 2024.12.16_18.09.05_Ch.tif]

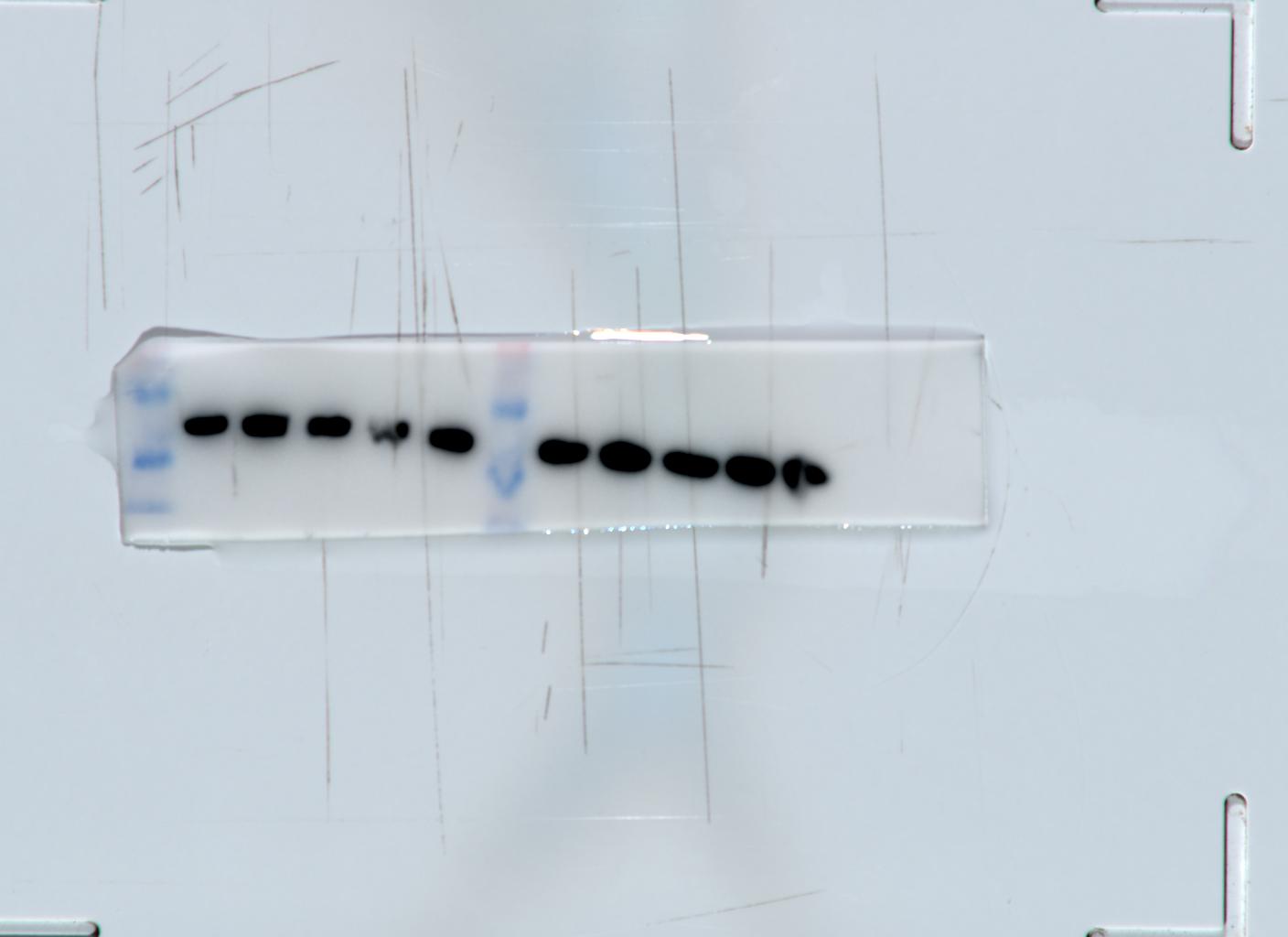

Supplement: Supplementary file 1 [file insects-16-00994-s001.zip › Figure S7/Figure 4D/3/SQSTM1/actin-3-4 2024.12.16_18.11.32_Ch/actin-2 2024.12.16_18.11.32_Ch+Marker.jpg]

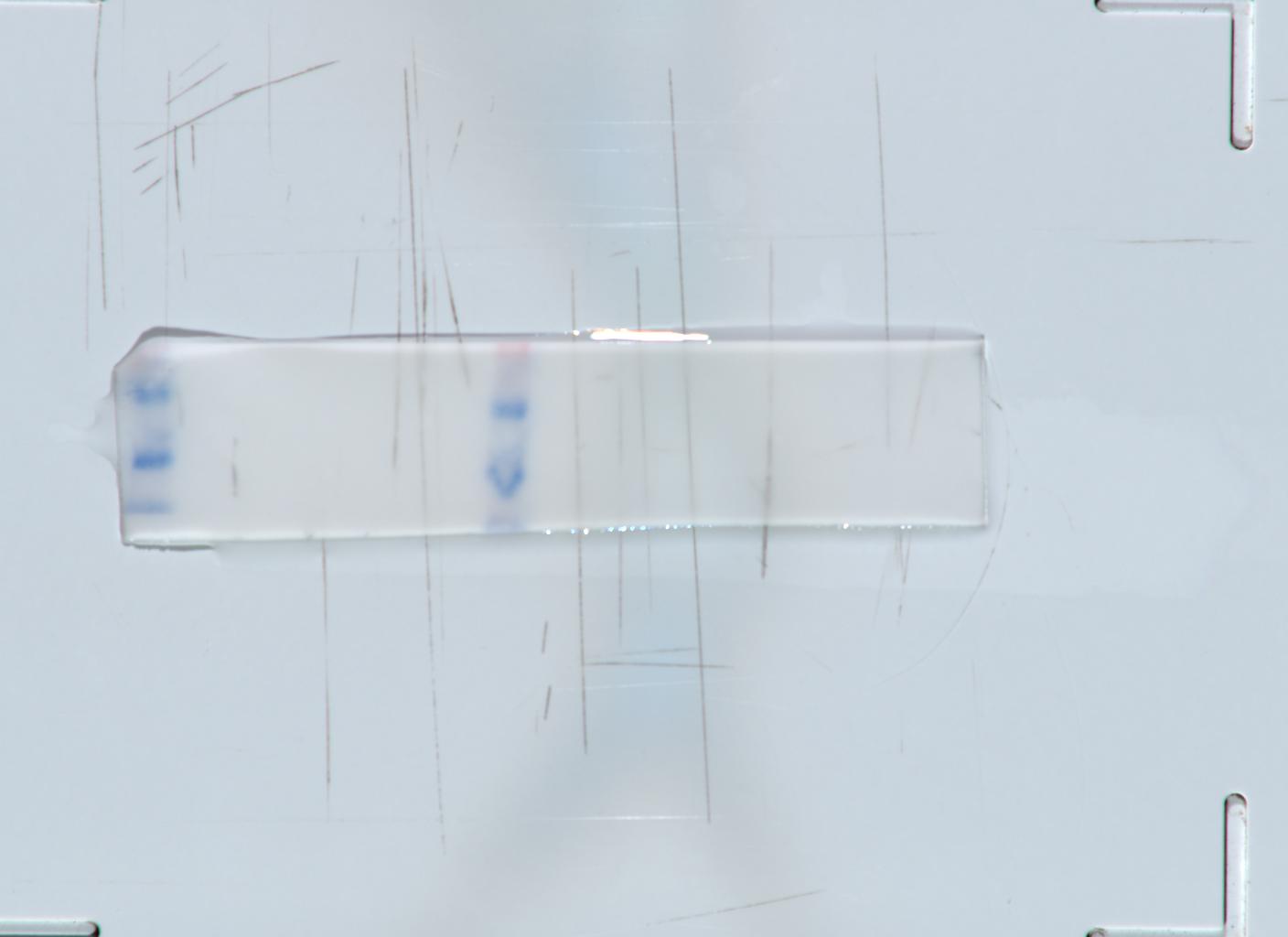

Supplement: Supplementary file 1 [file insects-16-00994-s001.zip › Figure S7/Figure 4D/3/SQSTM1/actin-3-4 2024.12.16_18.11.32_Ch/actin-2 2024.12.16_18.11.32_Ch-Marker.jpg]

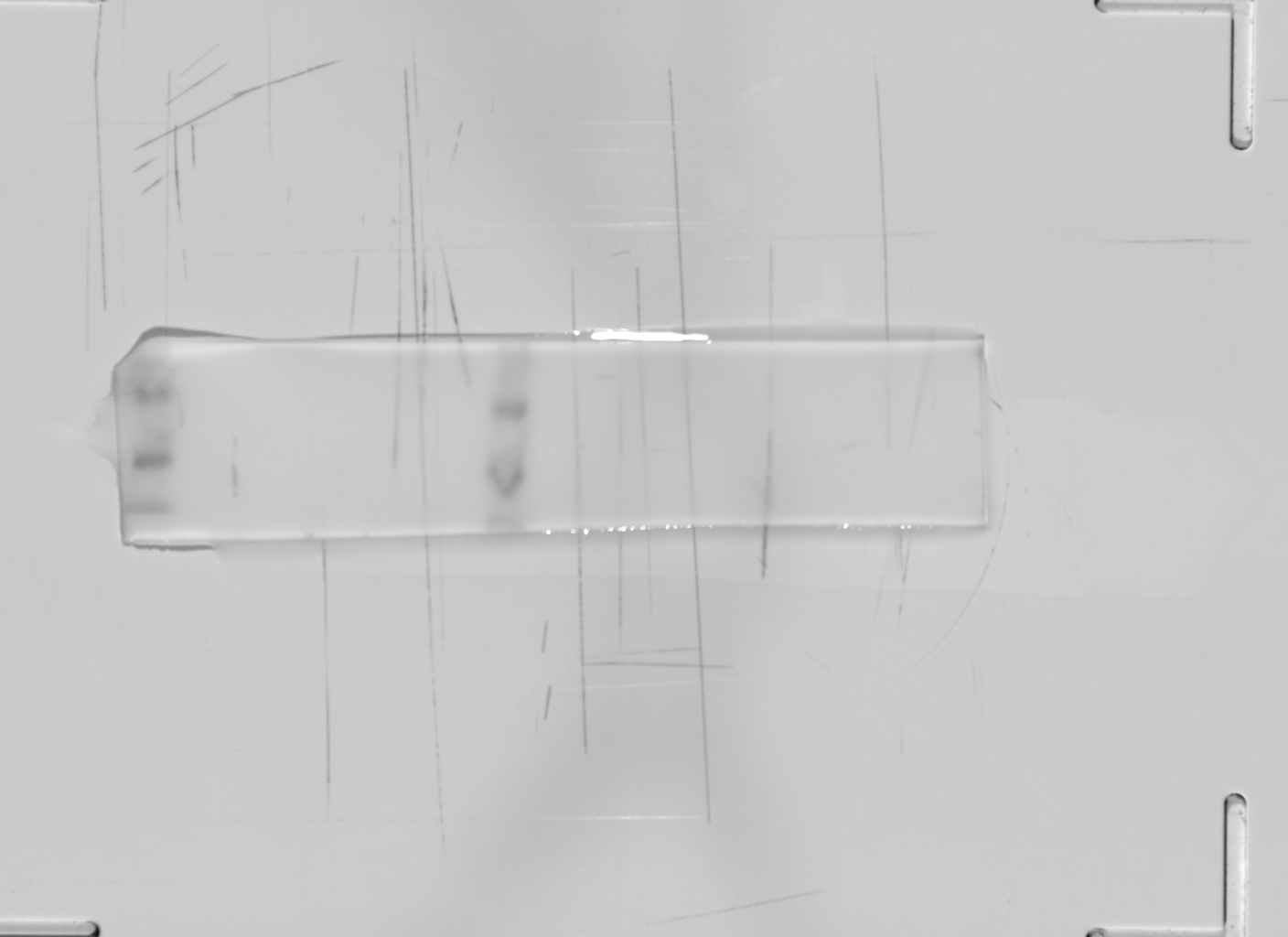

Supplement: Supplementary file 1 [file insects-16-00994-s001.zip › Figure S7/Figure 4D/3/SQSTM1/actin-3-4 2024.12.16_18.11.32_Ch/actin-2 2024.12.16_18.11.32_Ch-Marker.tif]

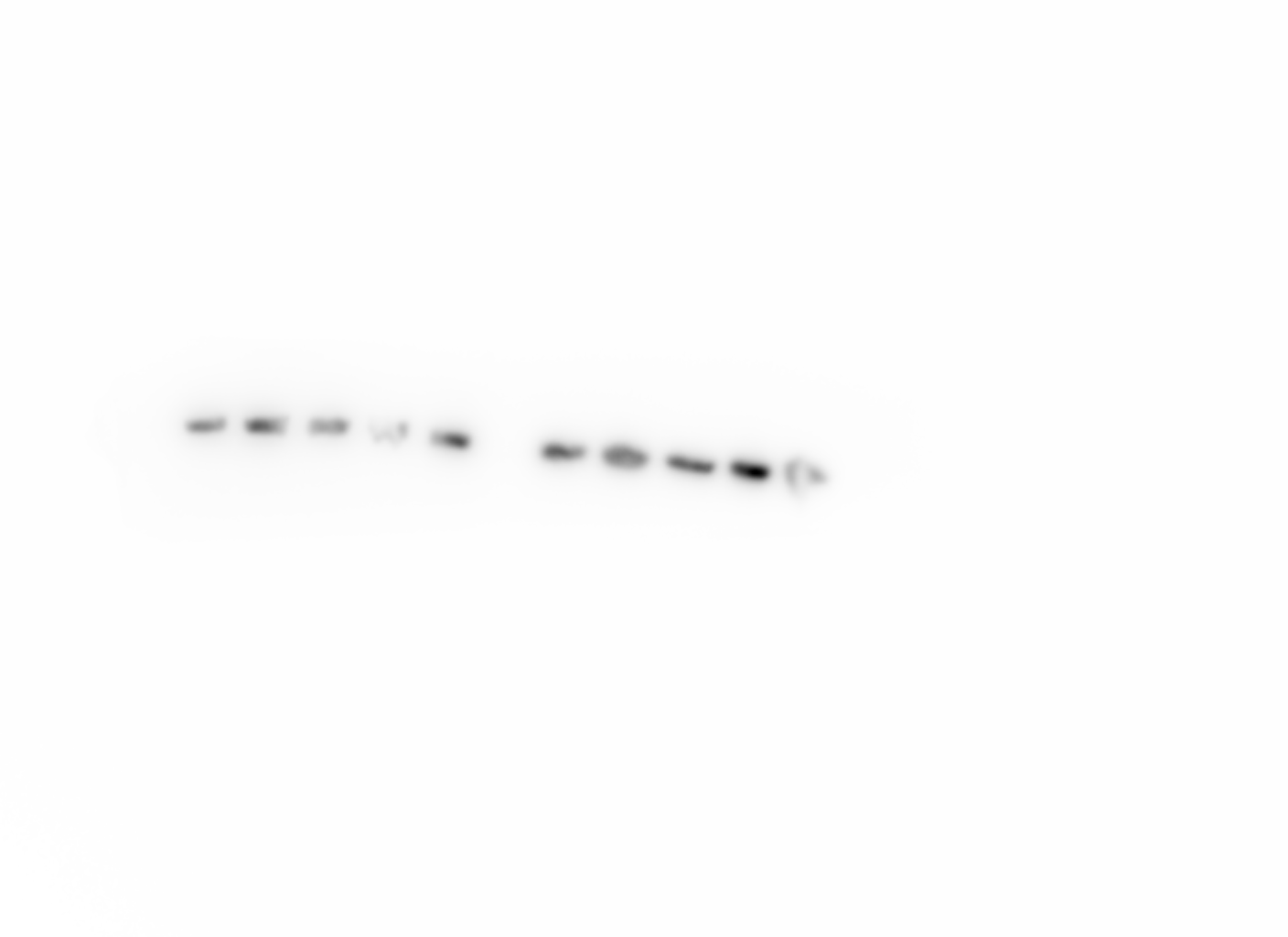

Supplement: Supplementary file 1 [file insects-16-00994-s001.zip › Figure S7/Figure 4D/3/SQSTM1/actin-3-4 2024.12.16_18.11.32_Ch/actin-2 2024.12.16_18.11.32_Ch.tif]

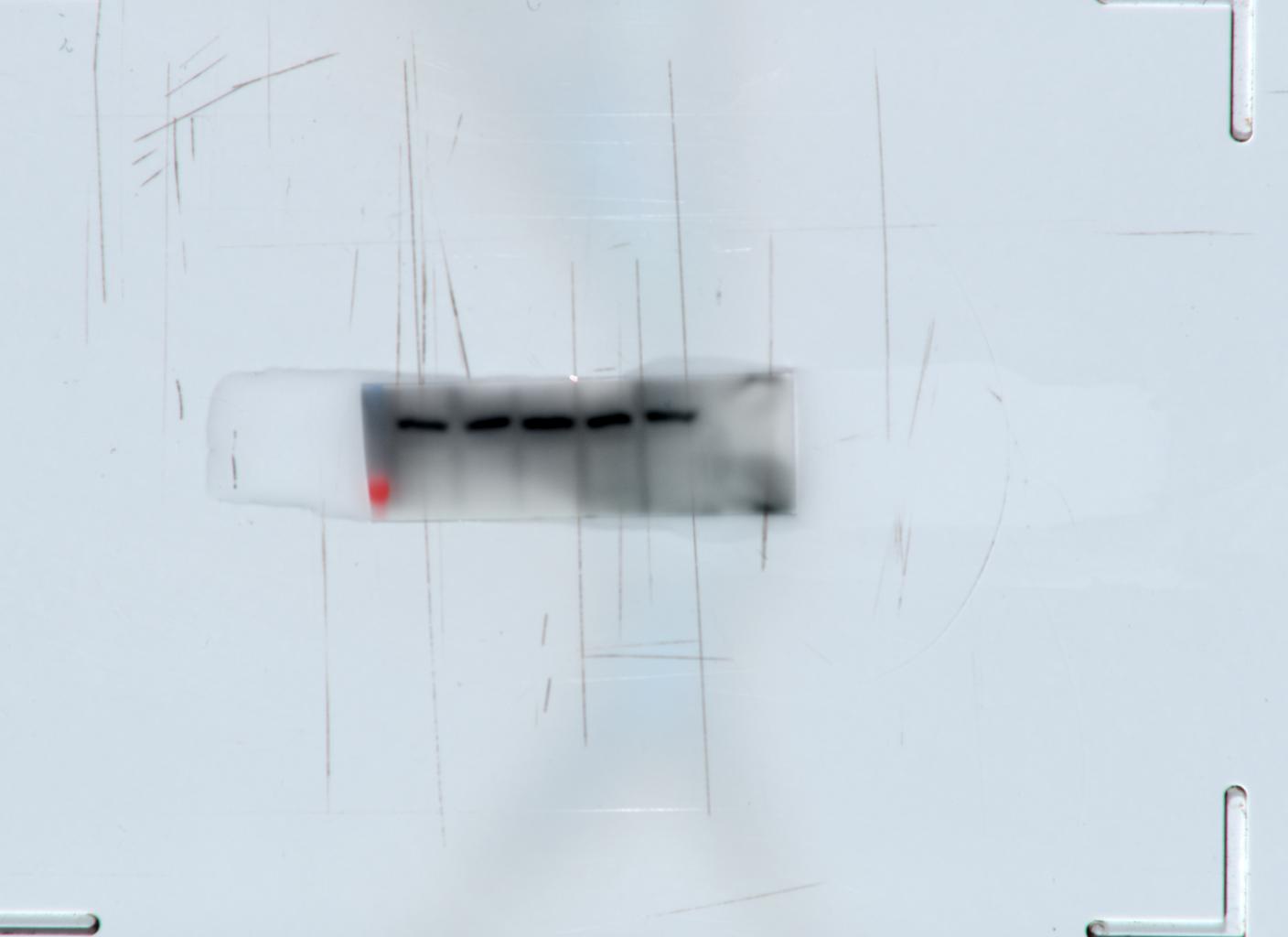

Supplement: Supplementary file 1 [file insects-16-00994-s001.zip › Figure S7/Figure 4D/3/SQSTM1/SQSTM1-1/p62-1 2024.12.16_18.18.16_Ch+Marker.jpg]

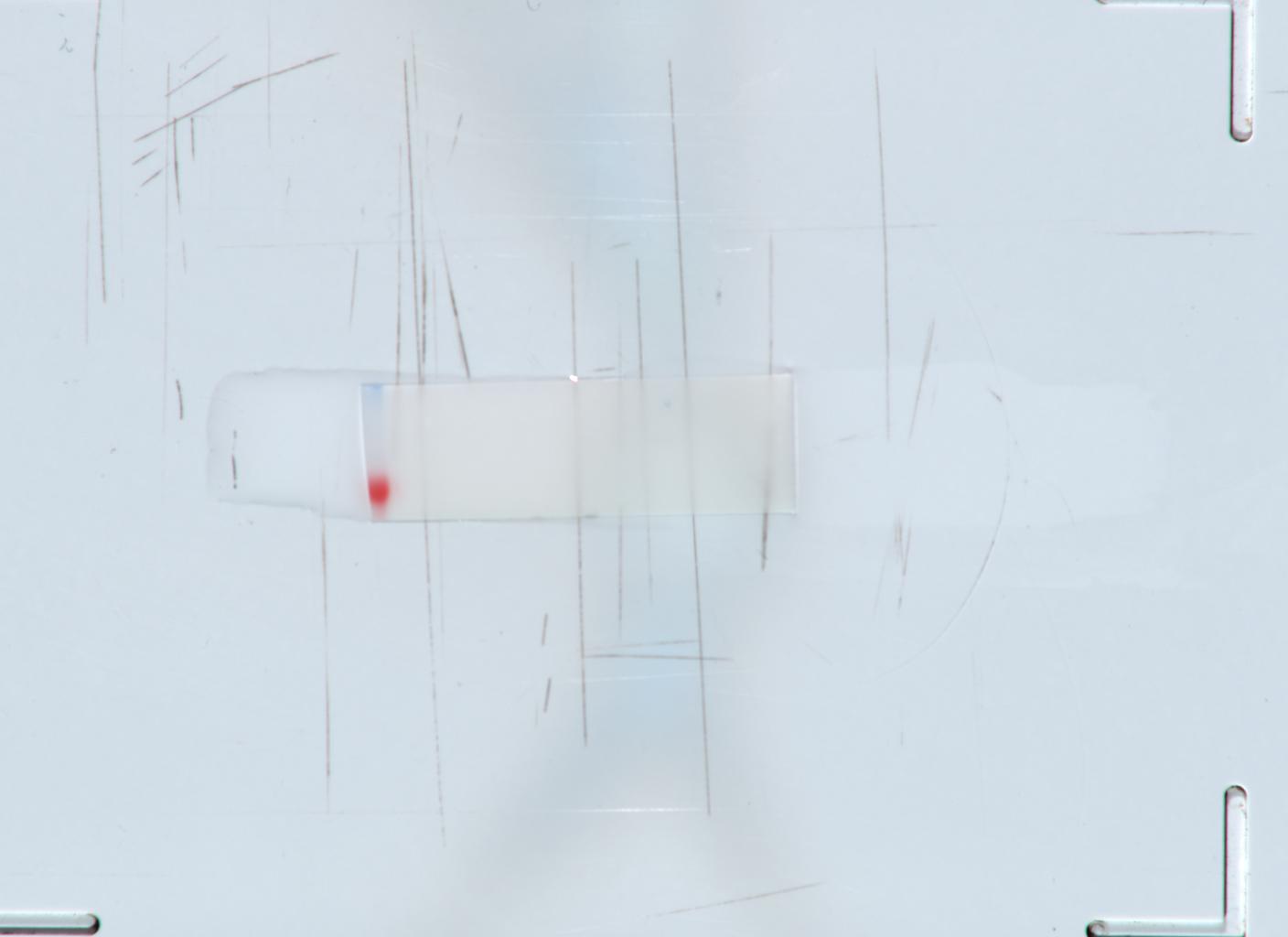

Supplement: Supplementary file 1 [file insects-16-00994-s001.zip › Figure S7/Figure 4D/3/SQSTM1/SQSTM1-1/p62-1 2024.12.16_18.18.16_Ch-Marker.jpg]

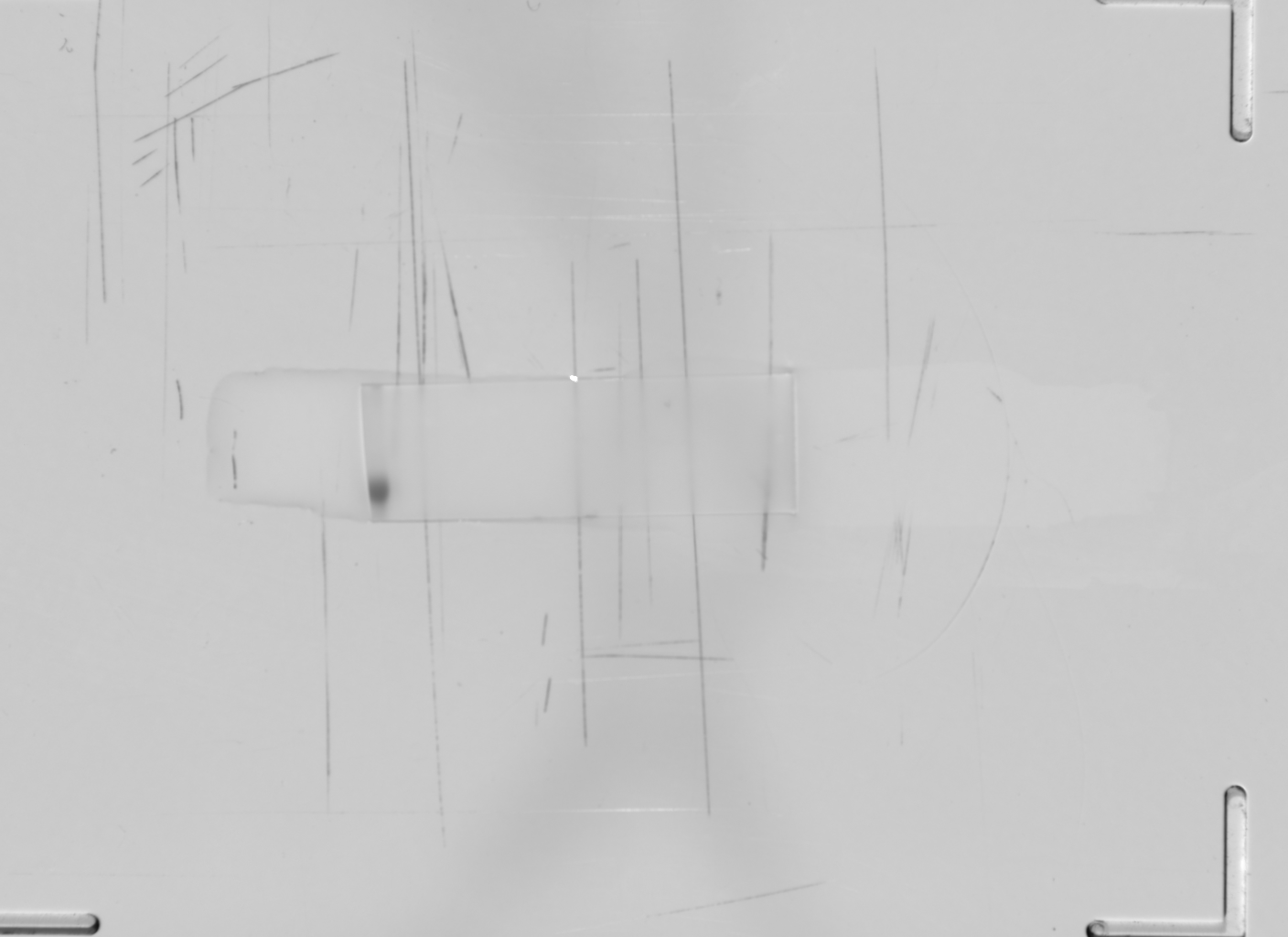

Supplement: Supplementary file 1 [file insects-16-00994-s001.zip › Figure S7/Figure 4D/3/SQSTM1/SQSTM1-1/p62-1 2024.12.16_18.18.16_Ch-Marker.tif]

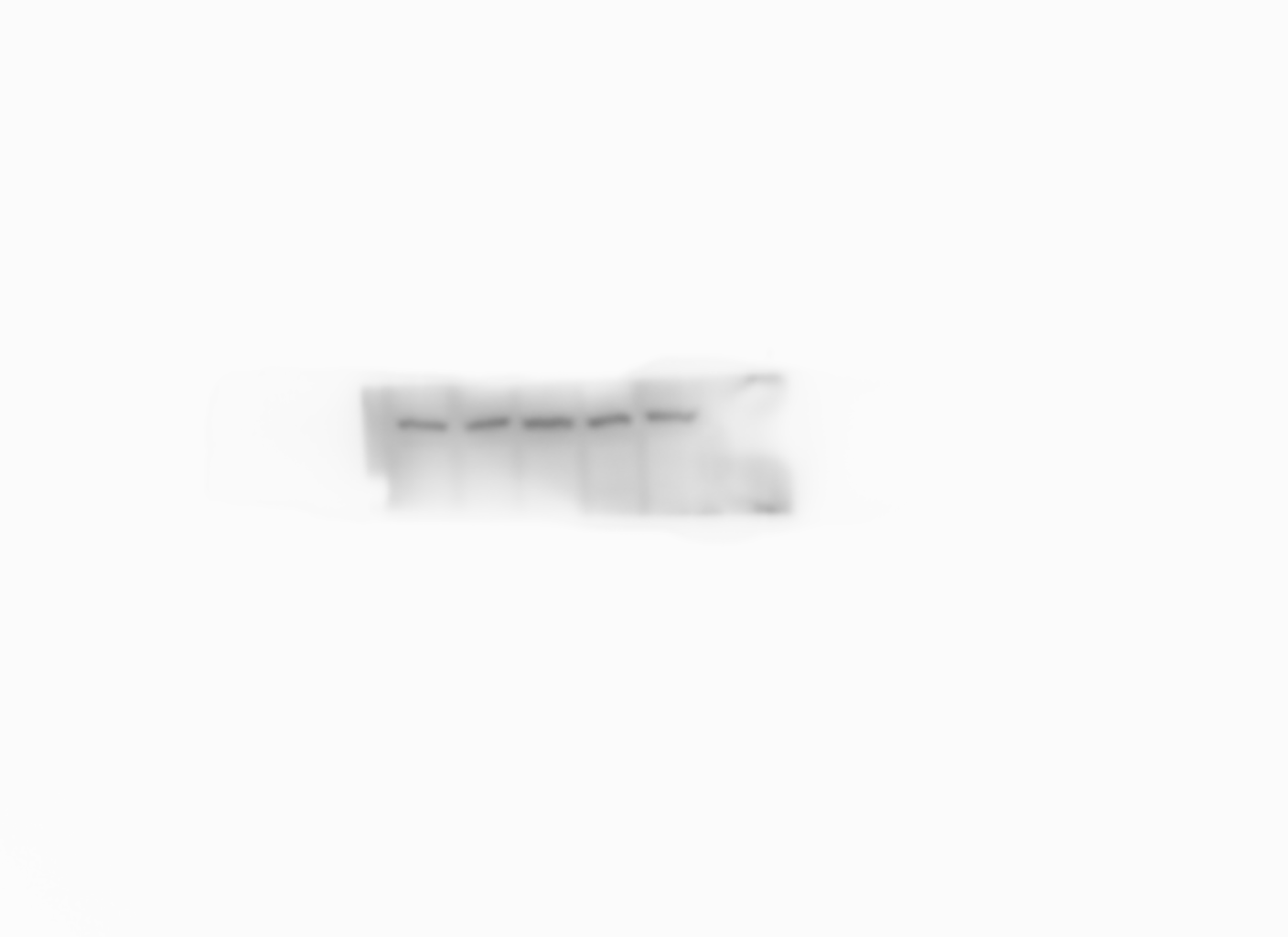

Supplement: Supplementary file 1 [file insects-16-00994-s001.zip › Figure S7/Figure 4D/3/SQSTM1/SQSTM1-1/p62-1 2024.12.16_18.18.16_Ch.tif]

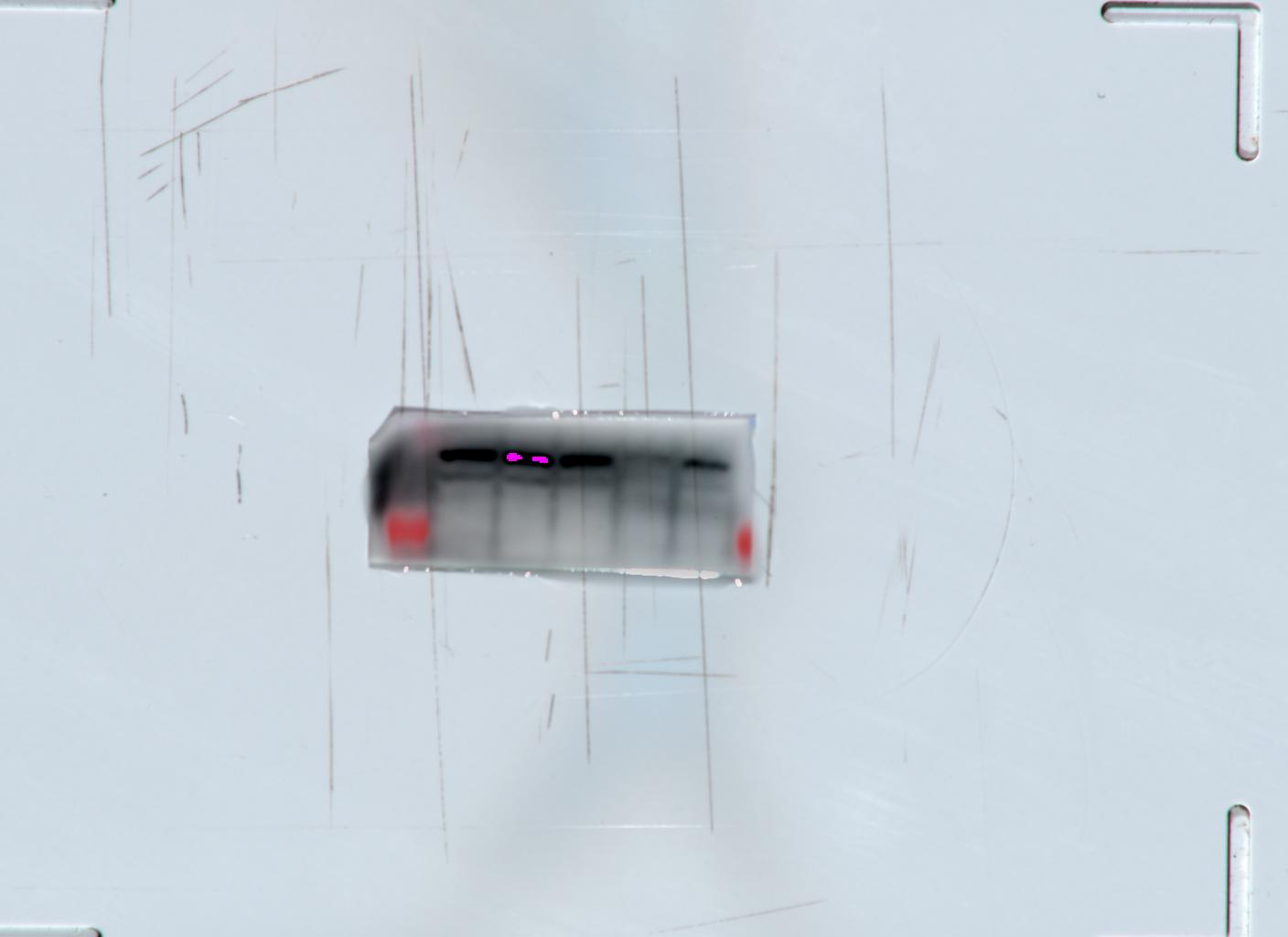

Supplement: Supplementary file 1 [file insects-16-00994-s001.zip › Figure S7/Figure 4D/3/SQSTM1/SQSTM1-2/p62--2 2024.12.16_18.47.23_Ch+Marker.jpg]

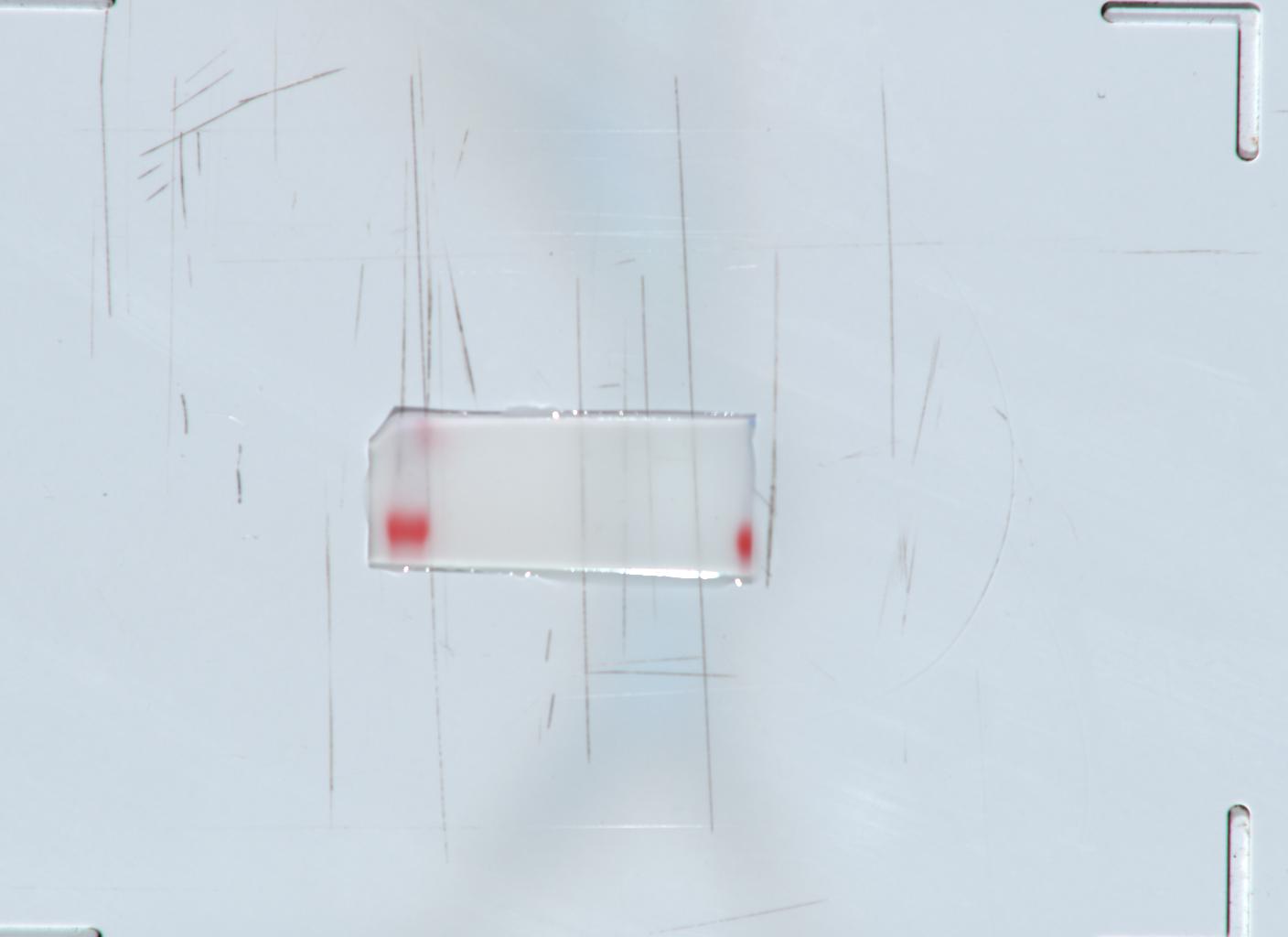

Supplement: Supplementary file 1 [file insects-16-00994-s001.zip › Figure S7/Figure 4D/3/SQSTM1/SQSTM1-2/p62--2 2024.12.16_18.47.23_Ch-Marker.jpg]

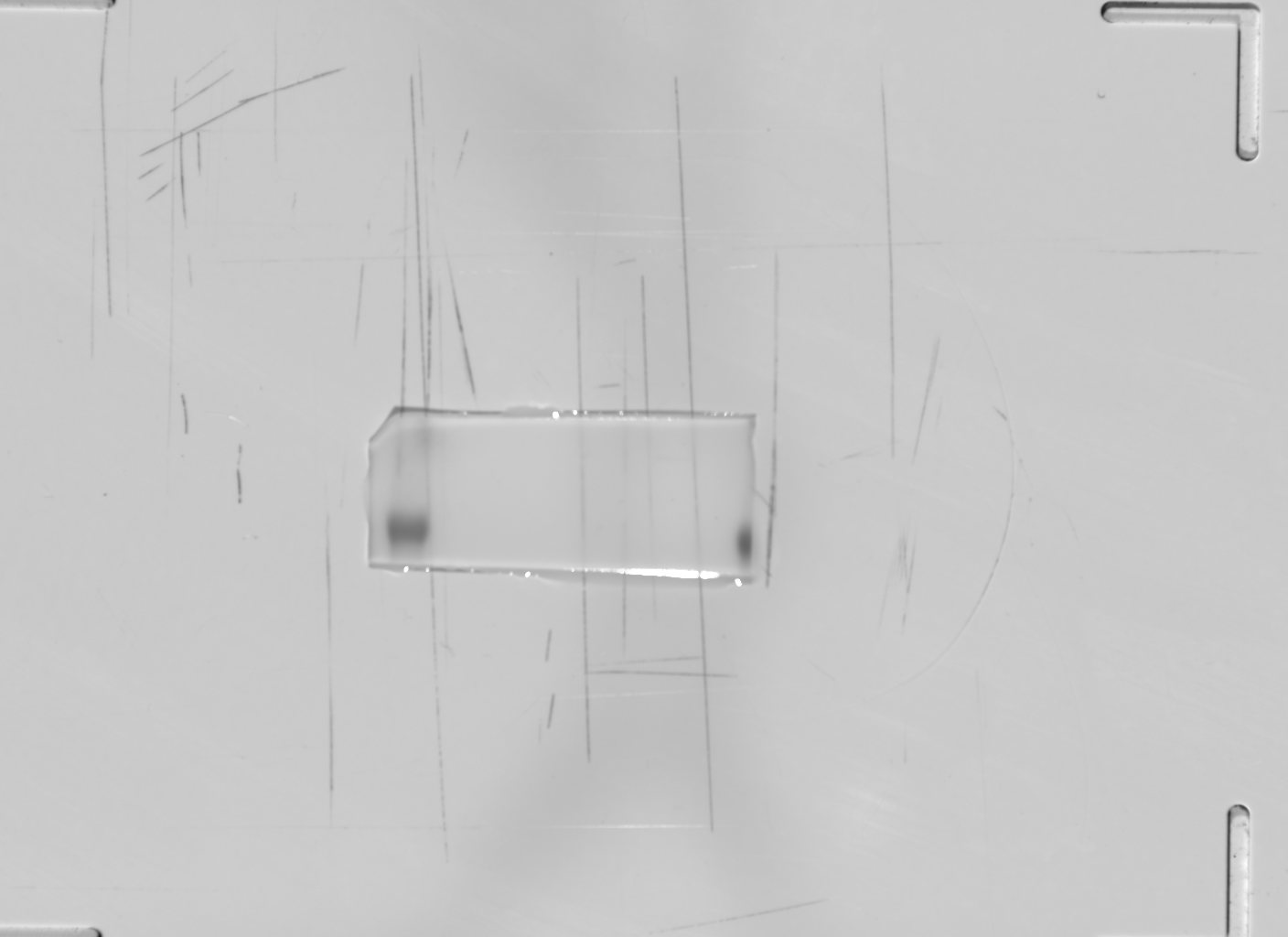

Supplement: Supplementary file 1 [file insects-16-00994-s001.zip › Figure S7/Figure 4D/3/SQSTM1/SQSTM1-2/p62--2 2024.12.16_18.47.23_Ch-Marker.tif]

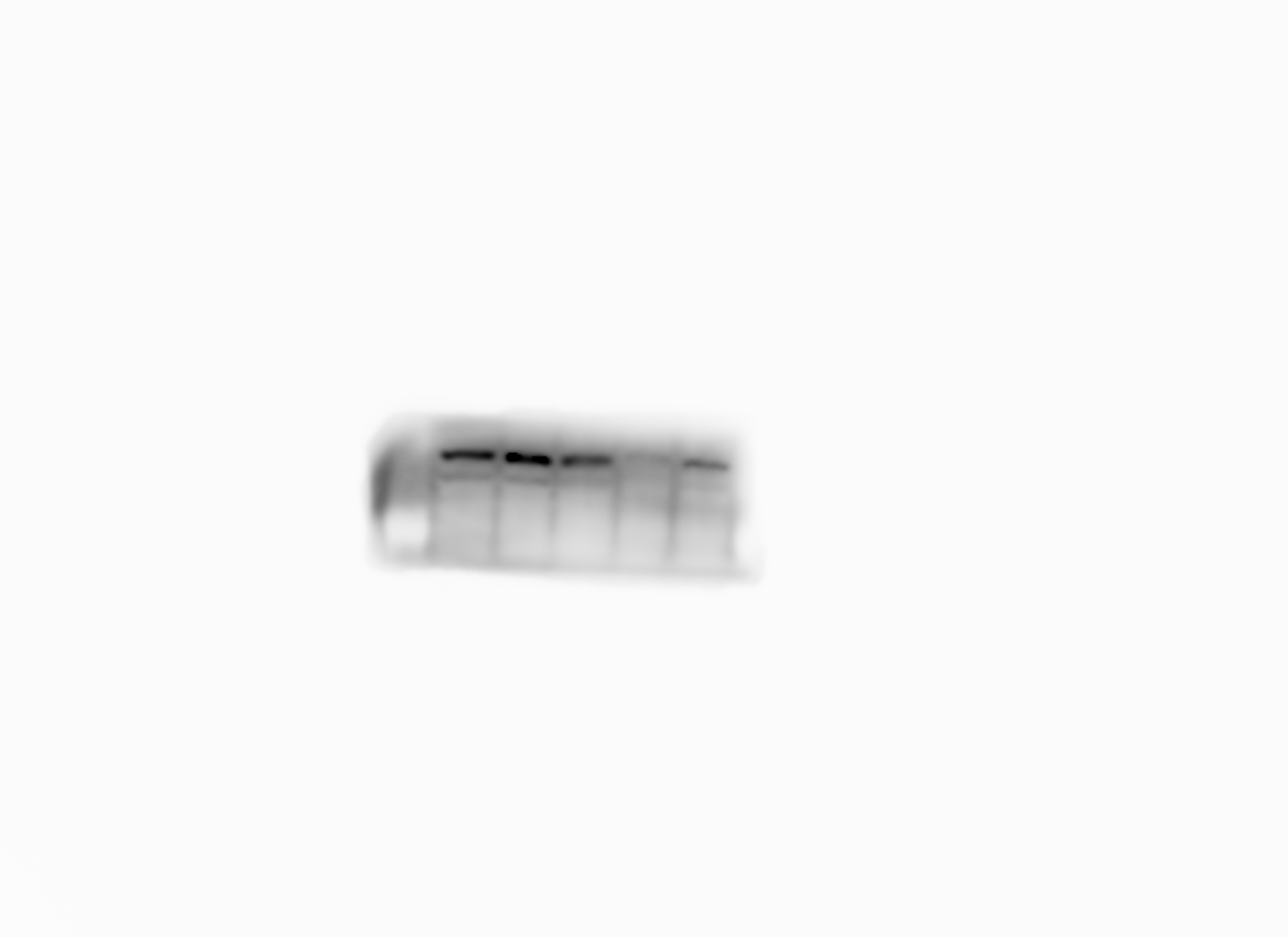

Supplement: Supplementary file 1 [file insects-16-00994-s001.zip › Figure S7/Figure 4D/3/SQSTM1/SQSTM1-2/p62--2 2024.12.16_18.47.23_Ch.tif]

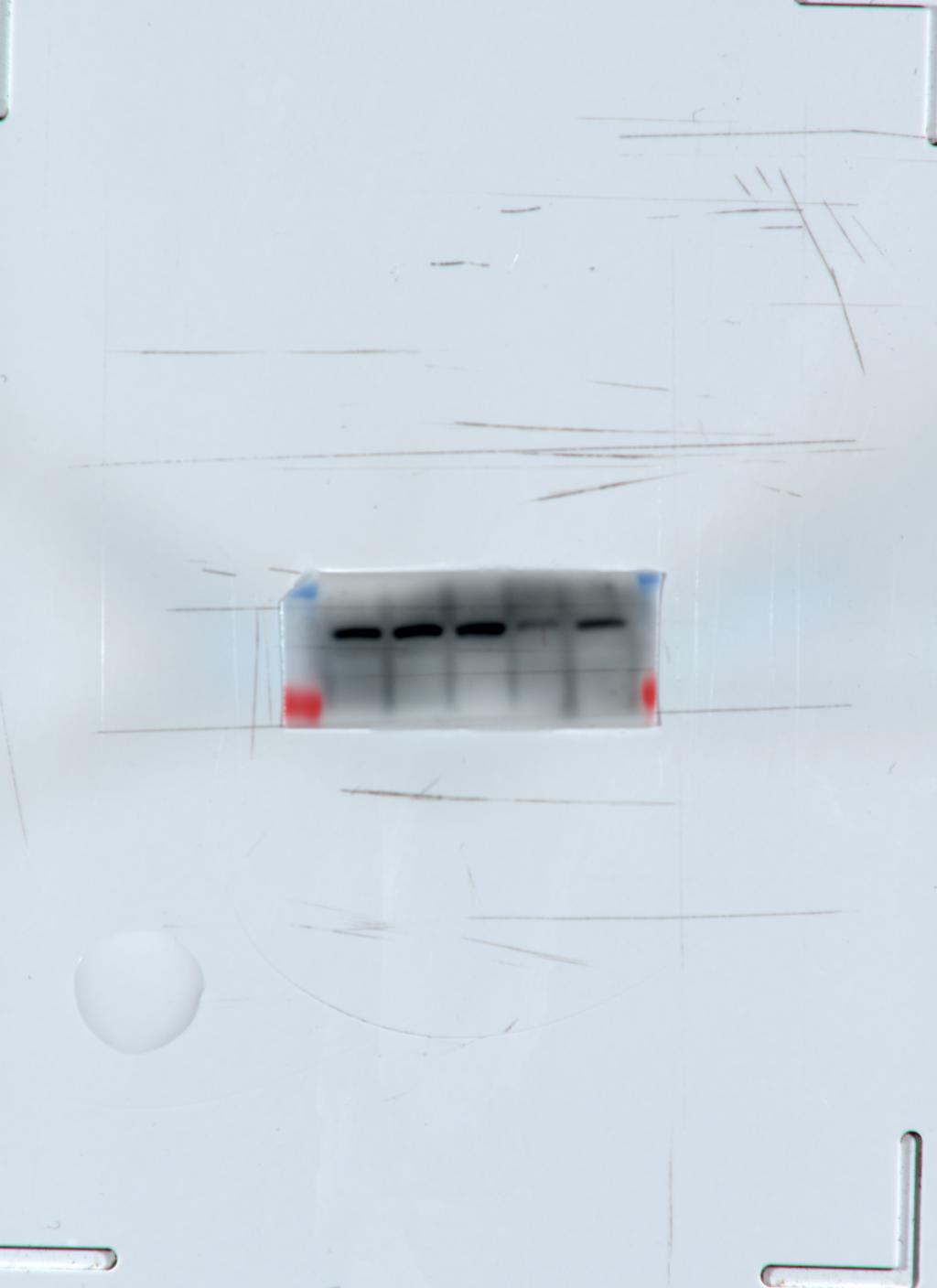

Supplement: Supplementary file 1 [file insects-16-00994-s001.zip › Figure S7/Figure 4D/3/SQSTM1/SQSTM1-3/p62-3 2024.12.16_18.24.14_Ch+Marker.jpg]

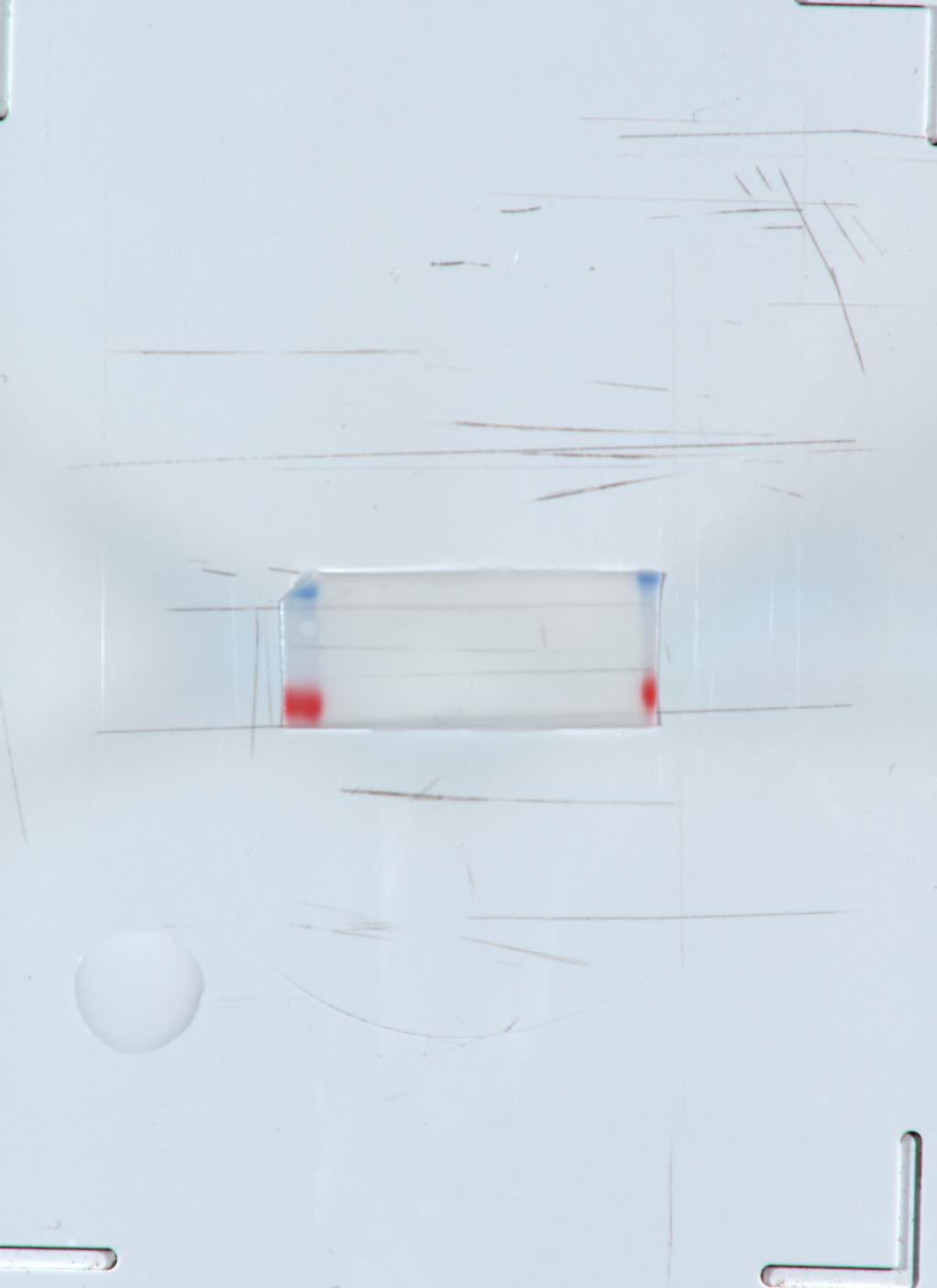

Supplement: Supplementary file 1 [file insects-16-00994-s001.zip › Figure S7/Figure 4D/3/SQSTM1/SQSTM1-3/p62-3 2024.12.16_18.24.14_Ch-Marker.jpg]

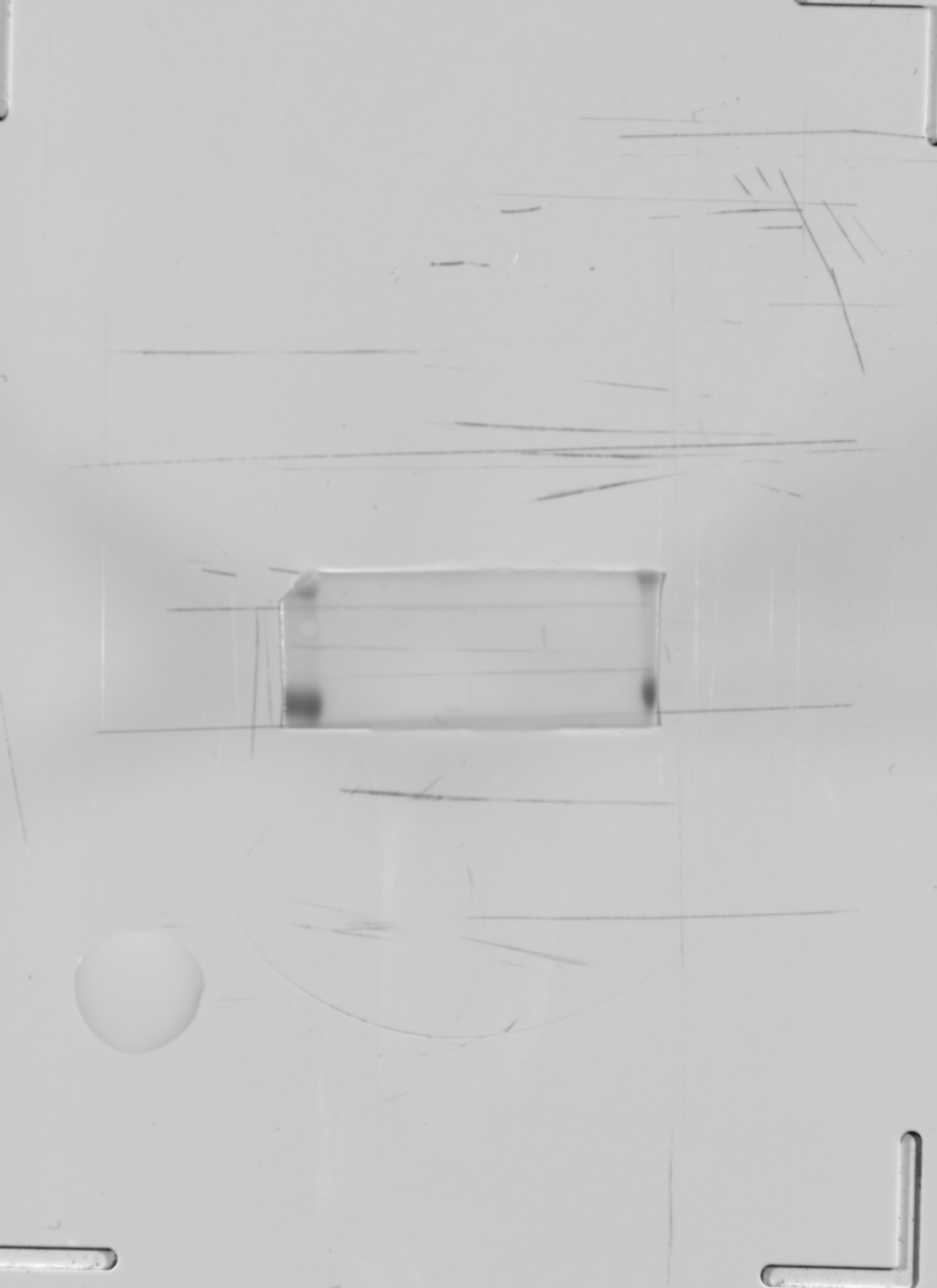

Supplement: Supplementary file 1 [file insects-16-00994-s001.zip › Figure S7/Figure 4D/3/SQSTM1/SQSTM1-3/p62-3 2024.12.16_18.24.14_Ch-Marker.tif]

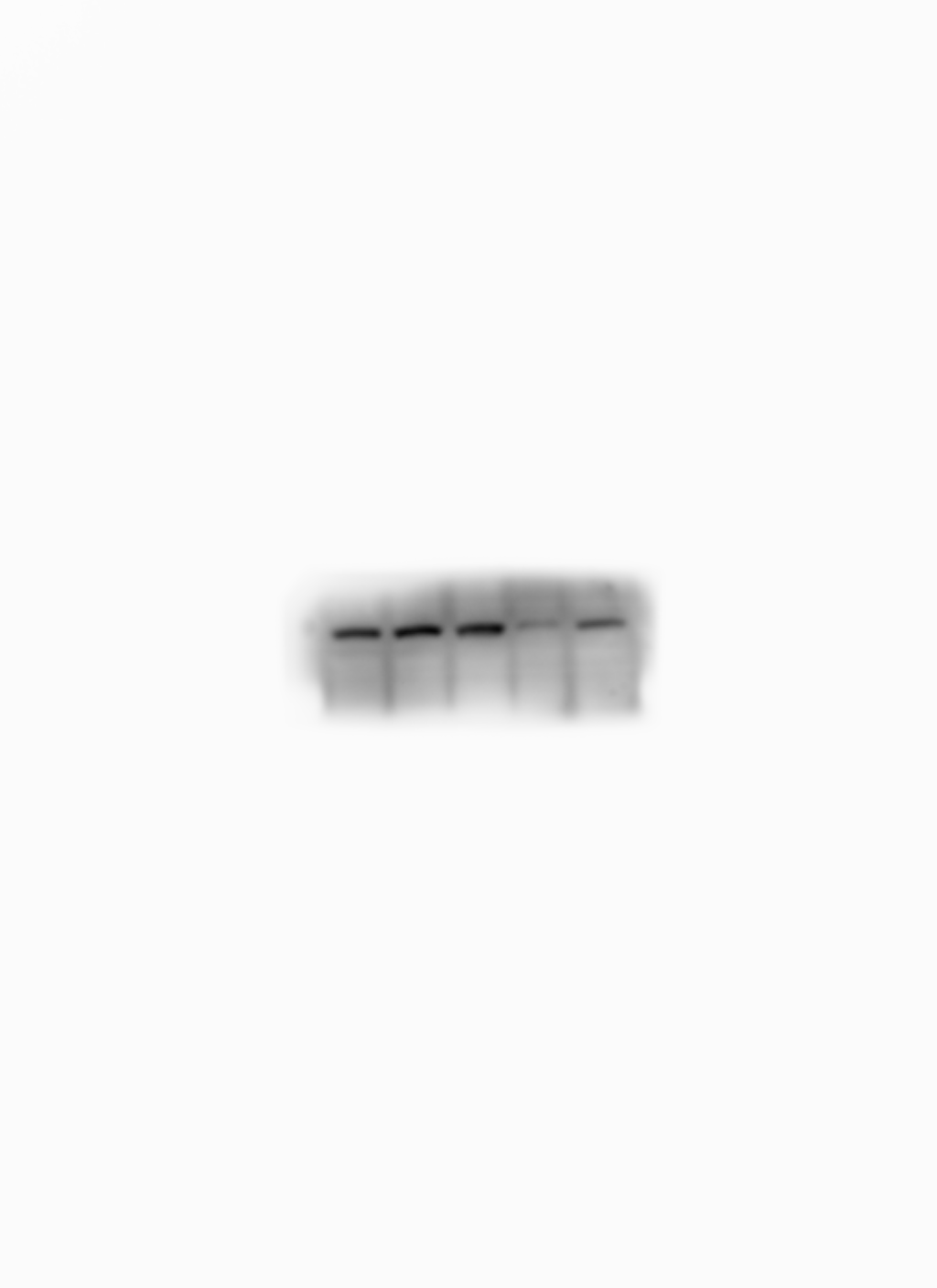

Supplement: Supplementary file 1 [file insects-16-00994-s001.zip › Figure S7/Figure 4D/3/SQSTM1/SQSTM1-3/p62-3 2024.12.16_18.24.14_Ch.tif]

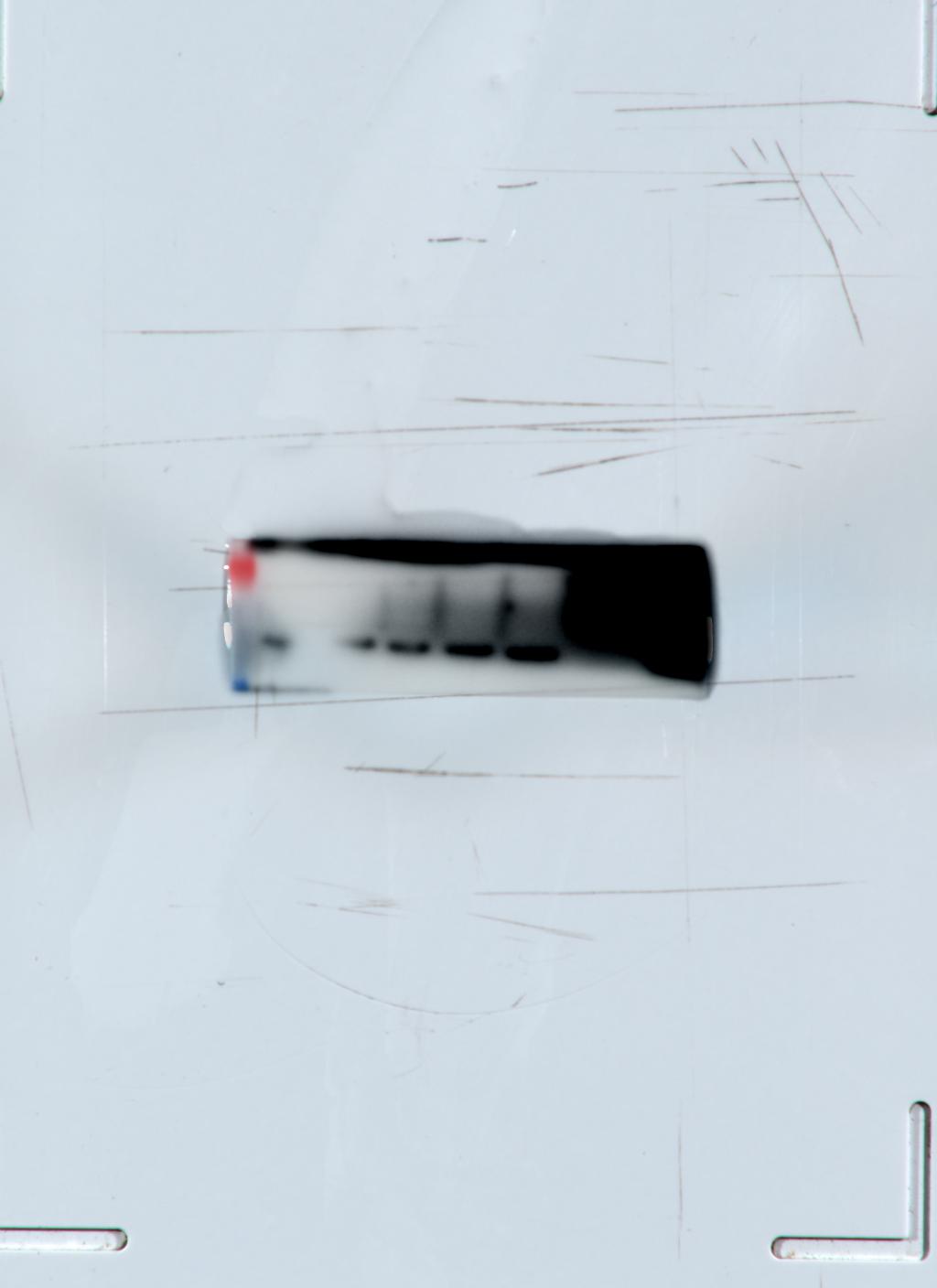

Supplement: Supplementary file 1 [file insects-16-00994-s001.zip › Figure S7/Figure 4D/3/SQSTM1/SQSTM1-4/p62-4 2024.12.16_18.29.12_Ch+Marker.jpg]

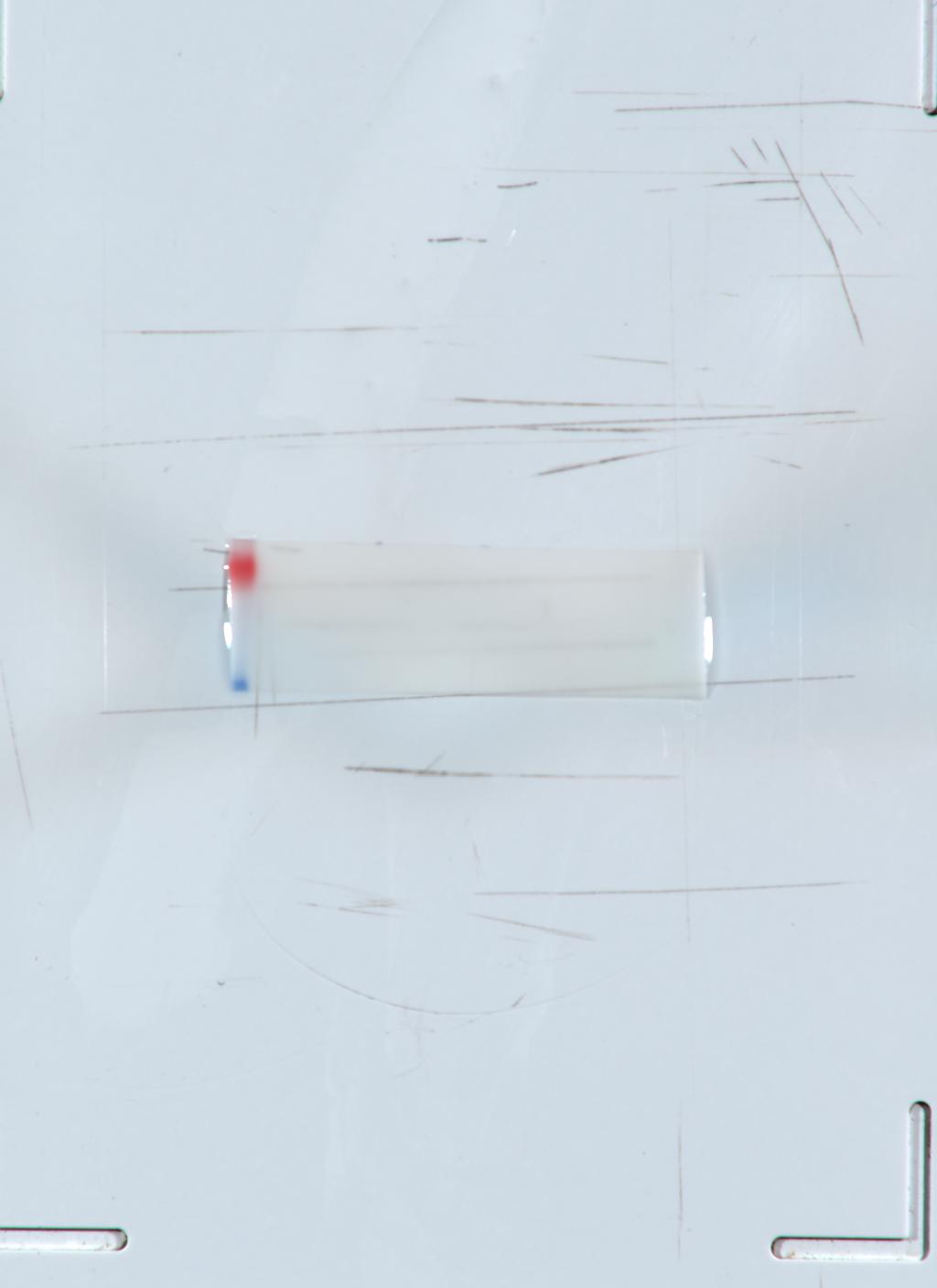

Supplement: Supplementary file 1 [file insects-16-00994-s001.zip › Figure S7/Figure 4D/3/SQSTM1/SQSTM1-4/p62-4 2024.12.16_18.29.12_Ch-Marker.jpg]

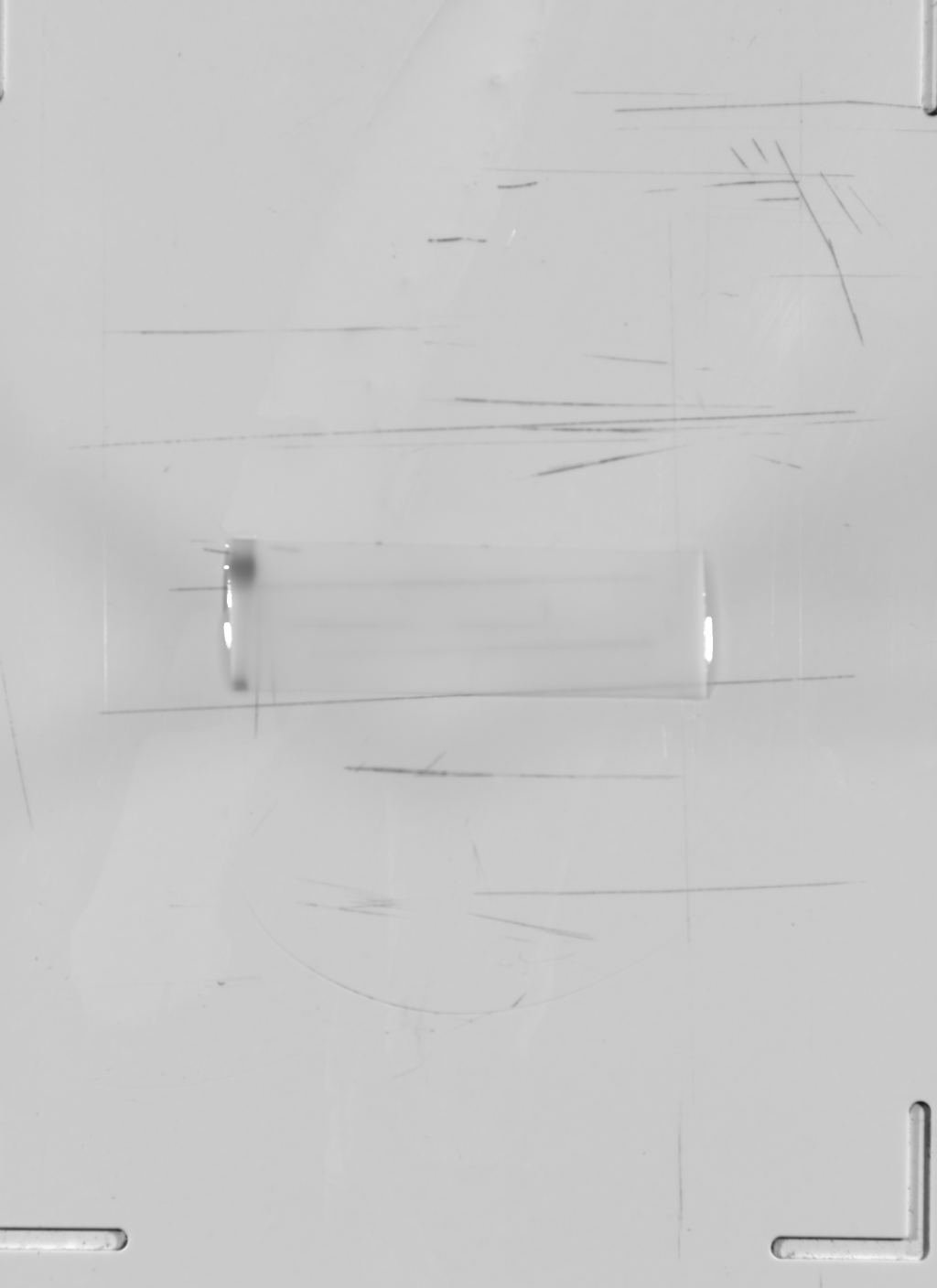

Supplement: Supplementary file 1 [file insects-16-00994-s001.zip › Figure S7/Figure 4D/3/SQSTM1/SQSTM1-4/p62-4 2024.12.16_18.29.12_Ch-Marker.tif]

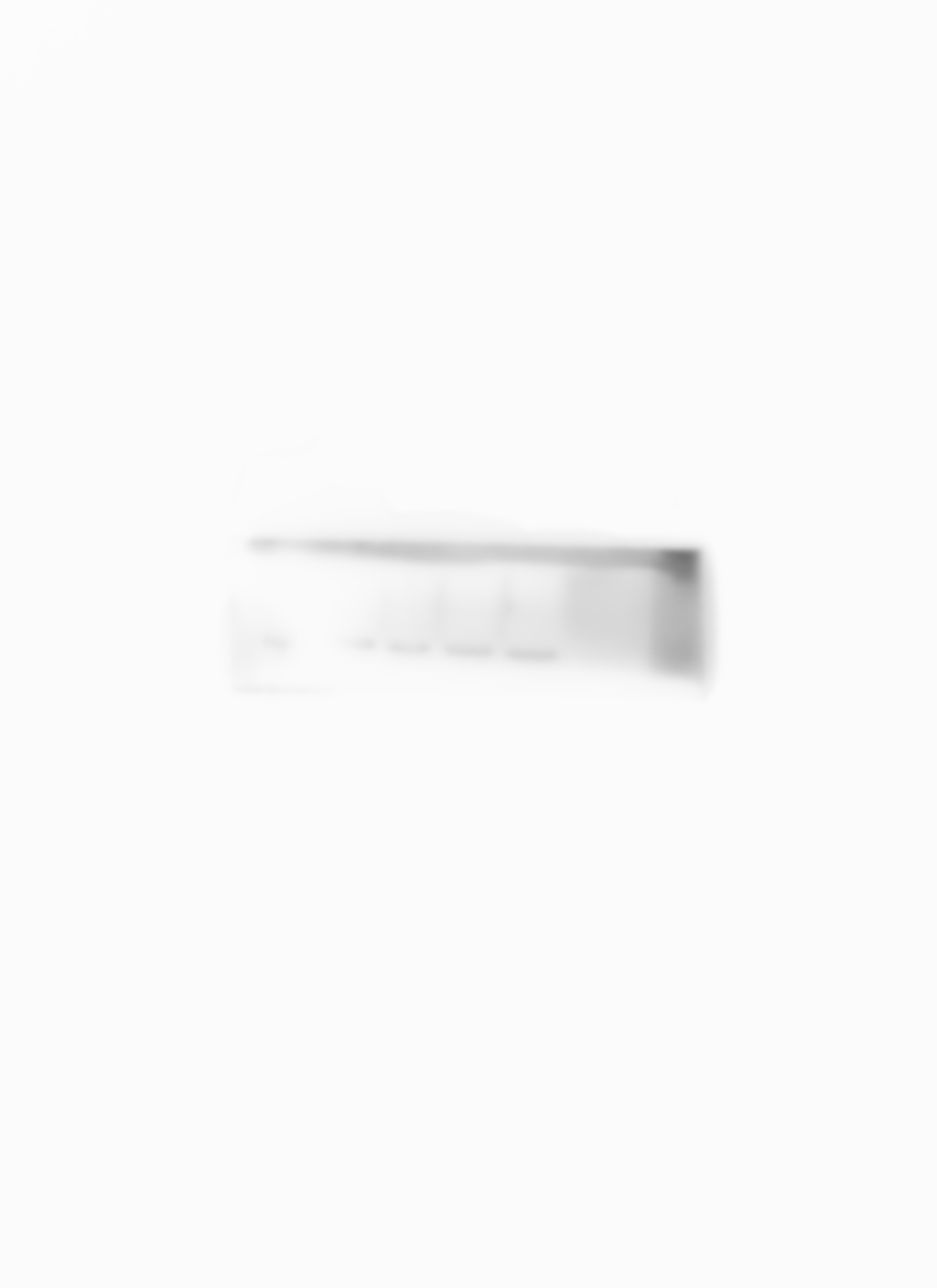

Supplement: Supplementary file 1 [file insects-16-00994-s001.zip › Figure S7/Figure 4D/3/SQSTM1/SQSTM1-4/p62-4 2024.12.16_18.29.12_Ch.tif]
