# Supplementary figures and images for: Identification, Expression of AaSQSTM1 in Aedes albopictus and Its Autophagic Function Analysis (part 1 of 2)
Source: Insects. 2025 Sep 24;16(10):994. doi: 10.3390/insects16100994 (PMC12564118; doi:10.3390/insects16100994)

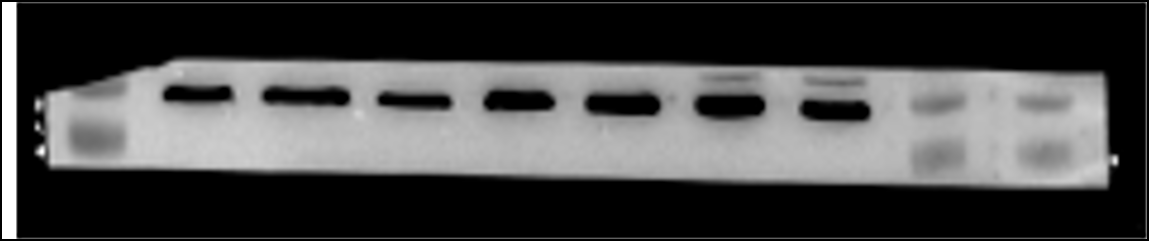

Supplement: Supplementary file 1 [file insects-16-00994-s001.zip › Figure S5/Figure 2B/1/actin(1).png]

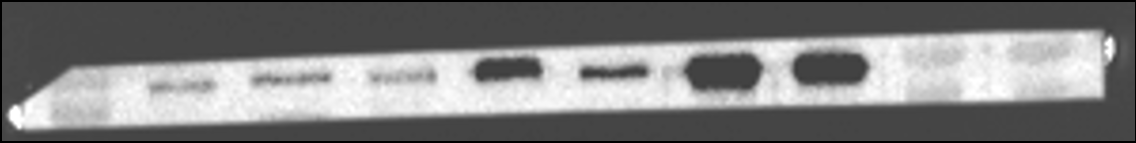

Supplement: Supplementary file 1 [file insects-16-00994-s001.zip › Figure S5/Figure 2B/1/sqstm1 (2).png]

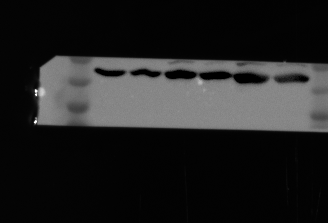

Supplement: Supplementary file 1 [file insects-16-00994-s001.zip › Figure S5/Figure 2B/2/ACTIN-1.tif]

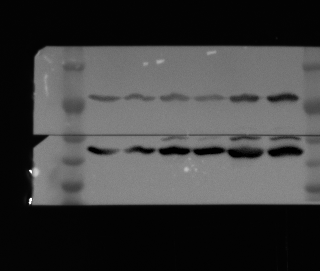

Supplement: Supplementary file 1 [file insects-16-00994-s001.zip › Figure S5/Figure 2B/2/ALL-1_8bit.tif]

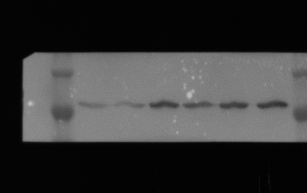

Supplement: Supplementary file 1 [file insects-16-00994-s001.zip › Figure S5/Figure 2B/2/SQSTM1-1.3.tif]

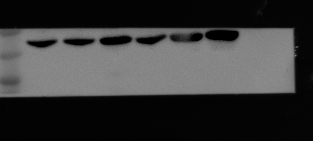

Supplement: Supplementary file 1 [file insects-16-00994-s001.zip › Figure S5/Figure 2B/3/ACTIN-2.tif]

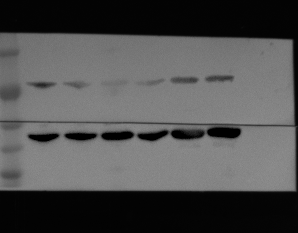

Supplement: Supplementary file 1 [file insects-16-00994-s001.zip › Figure S5/Figure 2B/3/ALL-2_8bit.tif]

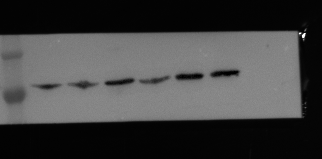

Supplement: Supplementary file 1 [file insects-16-00994-s001.zip › Figure S5/Figure 2B/3/SQSTM1-2_8bit.tif]

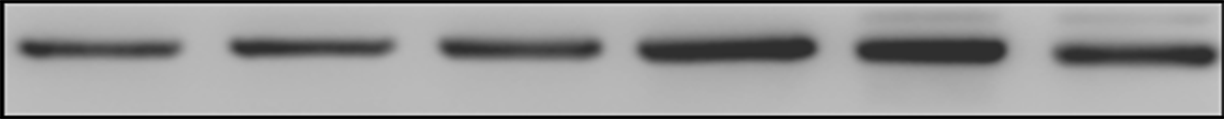

Supplement: Supplementary file 1 [file insects-16-00994-s001.zip › Figure S5/Figure 2E/1/actin-.png]

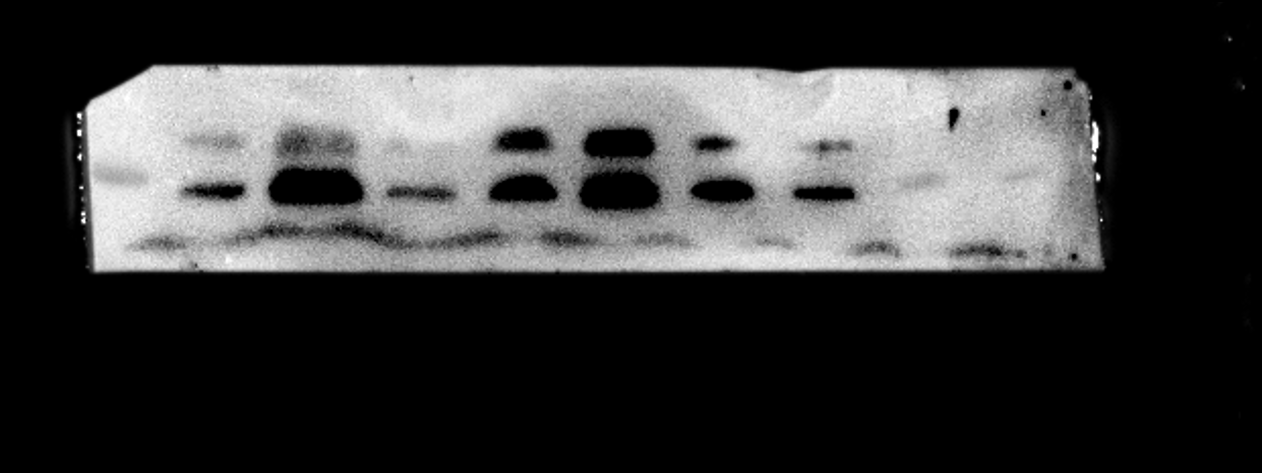

Supplement: Supplementary file 1 [file insects-16-00994-s001.zip › Figure S5/Figure 2E/1/atg8(1).png]

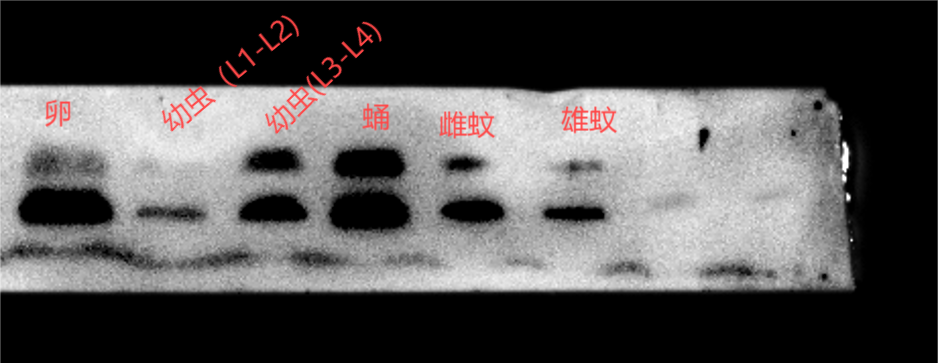

Supplement: Supplementary file 1 [file insects-16-00994-s001.zip › Figure S5/Figure 2E/1/atg8.png]

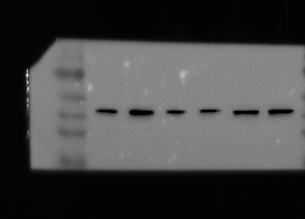

Supplement: Supplementary file 1 [file insects-16-00994-s001.zip › Figure S5/Figure 2E/2/actin-1_8bit.tif]

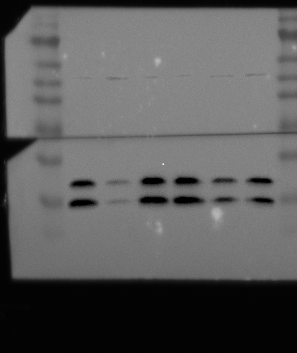

Supplement: Supplementary file 1 [file insects-16-00994-s001.zip › Figure S5/Figure 2E/2/all.png]

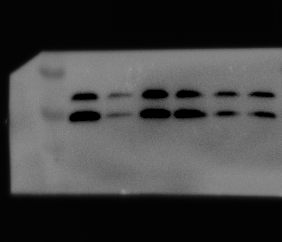

Supplement: Supplementary file 1 [file insects-16-00994-s001.zip › Figure S5/Figure 2E/2/atg8-1_8bit.tif]

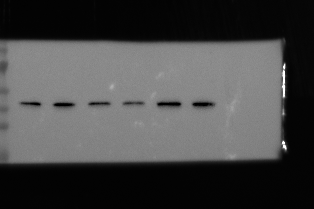

Supplement: Supplementary file 1 [file insects-16-00994-s001.zip › Figure S5/Figure 2E/3/actin-2_8bit.tif]

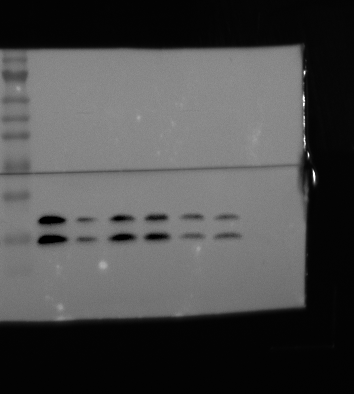

Supplement: Supplementary file 1 [file insects-16-00994-s001.zip › Figure S5/Figure 2E/3/all.png]

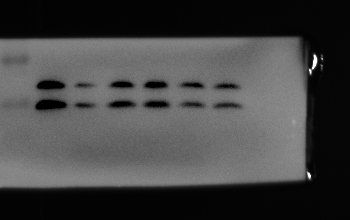

Supplement: Supplementary file 1 [file insects-16-00994-s001.zip › Figure S5/Figure 2E/3/atg8-2.tif]

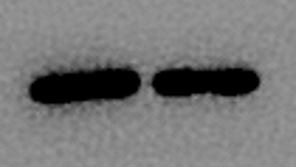

Supplement: Supplementary file 1 [file insects-16-00994-s001.zip › Figure S6/Figure 3B/1/Fatbody-actin.tif]

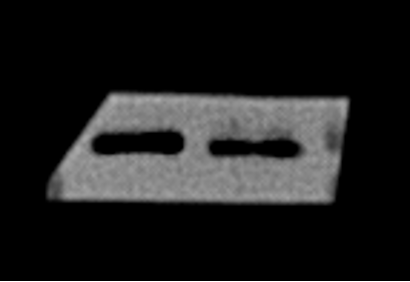

Supplement: Supplementary file 1 [file insects-16-00994-s001.zip › Figure S6/Figure 3B/1/Fatbody-sqstm1.tif]

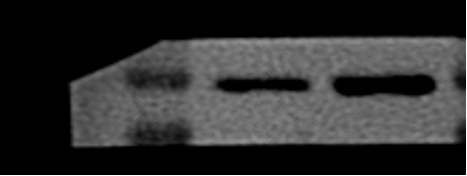

Supplement: Supplementary file 1 [file insects-16-00994-s001.zip › Figure S6/Figure 3B/1/Head-actin.tif]

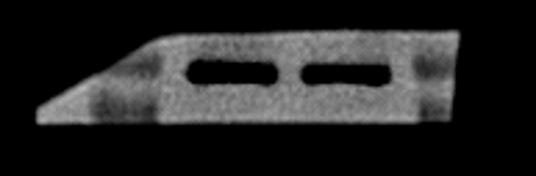

Supplement: Supplementary file 1 [file insects-16-00994-s001.zip › Figure S6/Figure 3B/1/Head-sqstm1.tif]

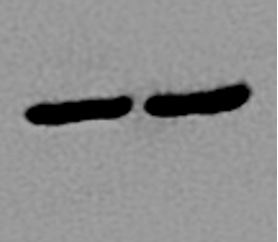

Supplement: Supplementary file 1 [file insects-16-00994-s001.zip › Figure S6/Figure 3B/1/Midgut-actin.tif]

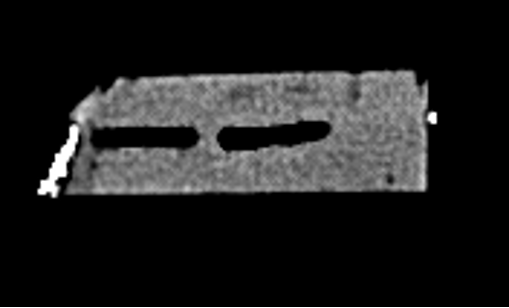

Supplement: Supplementary file 1 [file insects-16-00994-s001.zip › Figure S6/Figure 3B/1/Midgut-sqstm1.tif]

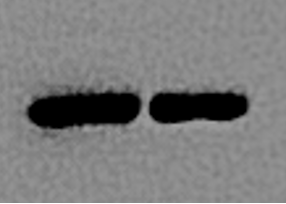

Supplement: Supplementary file 1 [file insects-16-00994-s001.zip › Figure S6/Figure 3B/1/Ovary-actin.tif]

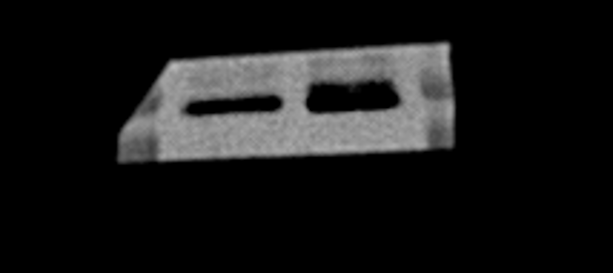

Supplement: Supplementary file 1 [file insects-16-00994-s001.zip › Figure S6/Figure 3B/1/Ovary-sqstm1.tif]

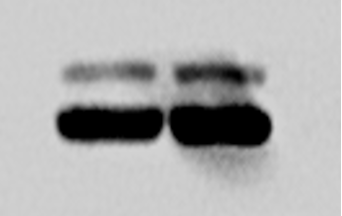

Supplement: Supplementary file 1 [file insects-16-00994-s001.zip › Figure S6/Figure 3B/1/Thorax-actin.tif]

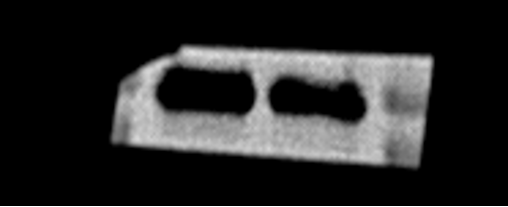

Supplement: Supplementary file 1 [file insects-16-00994-s001.zip › Figure S6/Figure 3B/1/Thorax-sqstm1.tif]

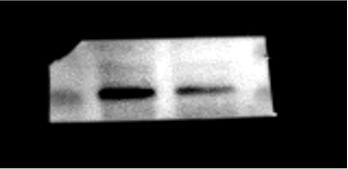

Supplement: Supplementary file 1 [file insects-16-00994-s001.zip › Figure S6/Figure 3B/2/Fatbody-actin.tif]

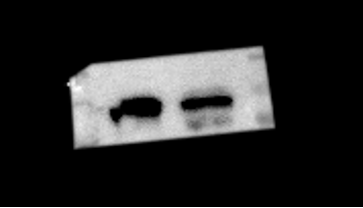

Supplement: Supplementary file 1 [file insects-16-00994-s001.zip › Figure S6/Figure 3B/2/Fatbody-p62.tif]

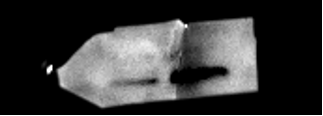

Supplement: Supplementary file 1 [file insects-16-00994-s001.zip › Figure S6/Figure 3B/2/Head-actin.tif]

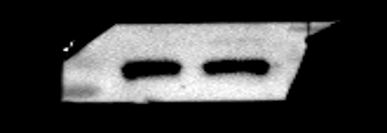

Supplement: Supplementary file 1 [file insects-16-00994-s001.zip › Figure S6/Figure 3B/2/Head-p62.tif]

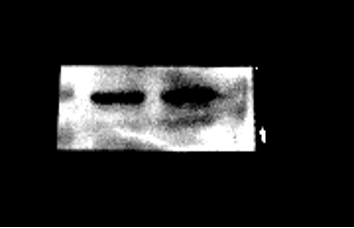

Supplement: Supplementary file 1 [file insects-16-00994-s001.zip › Figure S6/Figure 3B/2/Midgut-actin.tif]

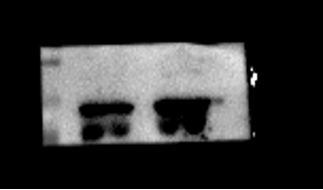

Supplement: Supplementary file 1 [file insects-16-00994-s001.zip › Figure S6/Figure 3B/2/Midgut-p62.tif]

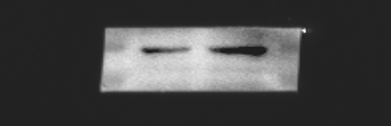

Supplement: Supplementary file 1 [file insects-16-00994-s001.zip › Figure S6/Figure 3B/2/Ovary-actin.tif]

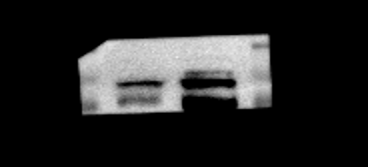

Supplement: Supplementary file 1 [file insects-16-00994-s001.zip › Figure S6/Figure 3B/2/Ovary-p62.tif]

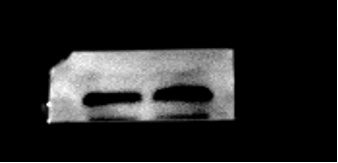

Supplement: Supplementary file 1 [file insects-16-00994-s001.zip › Figure S6/Figure 3B/2/Thorax-actin.tif]

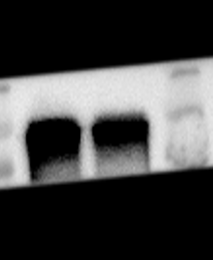

Supplement: Supplementary file 1 [file insects-16-00994-s001.zip › Figure S6/Figure 3B/2/Thorax-p62.tif]

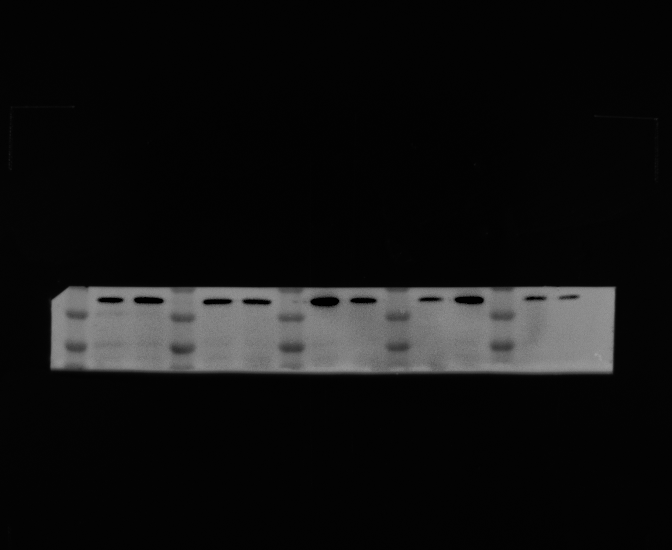

Supplement: Supplementary file 1 [file insects-16-00994-s001.zip › Figure S6/Figure 3B/3/ACTIN-3.tif]

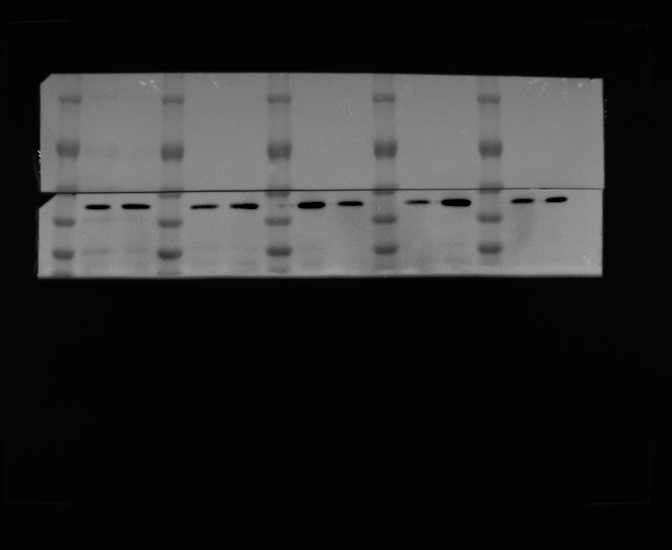

Supplement: Supplementary file 1 [file insects-16-00994-s001.zip › Figure S6/Figure 3B/3/ALL-3.tif]

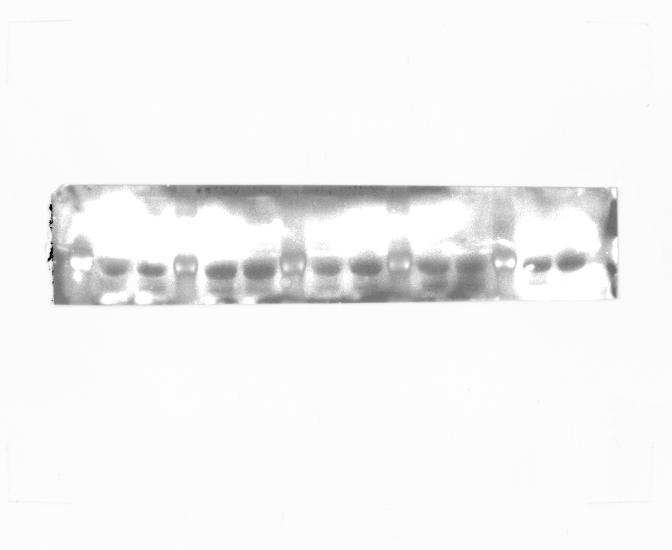

Supplement: Supplementary file 1 [file insects-16-00994-s001.zip › Figure S6/Figure 3B/3/SQSTM1-3.tif]

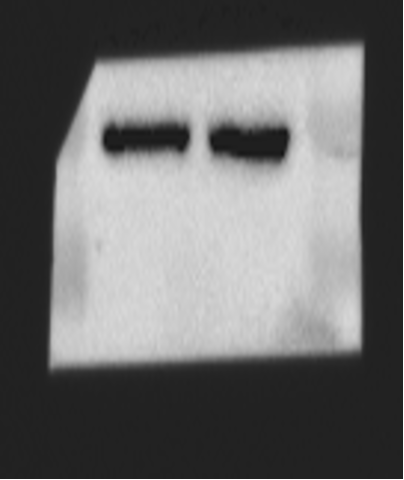

Supplement: Supplementary file 1 [file insects-16-00994-s001.zip › Figure S6/Figure 3E/1/Fatbody-actin.tif]

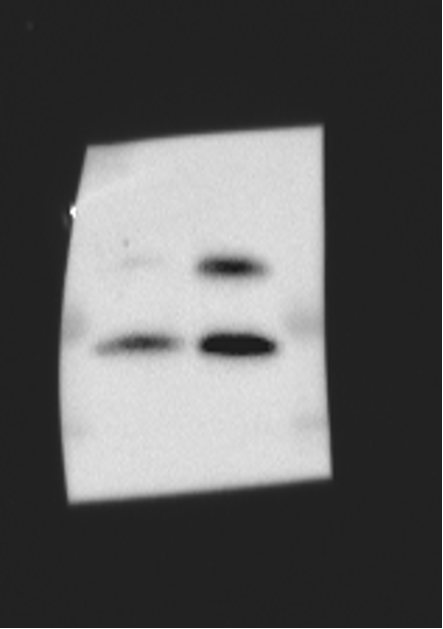

Supplement: Supplementary file 1 [file insects-16-00994-s001.zip › Figure S6/Figure 3E/1/Fatbody-atg8.tif]

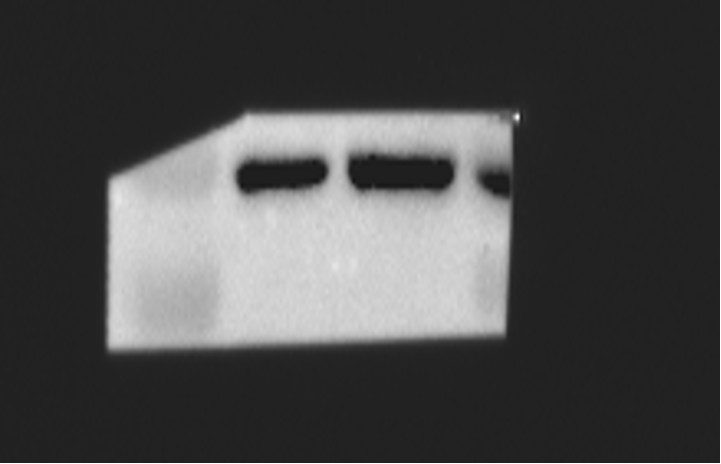

Supplement: Supplementary file 1 [file insects-16-00994-s001.zip › Figure S6/Figure 3E/1/Head-actin.tif]

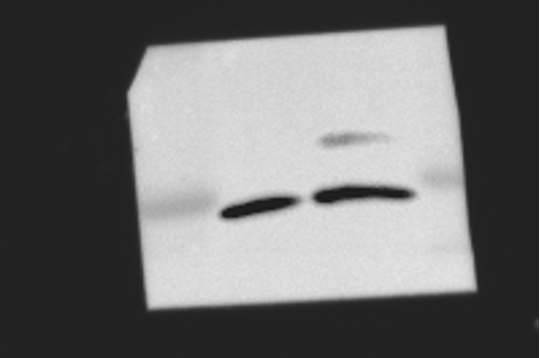

Supplement: Supplementary file 1 [file insects-16-00994-s001.zip › Figure S6/Figure 3E/1/Head-atg8.tif]

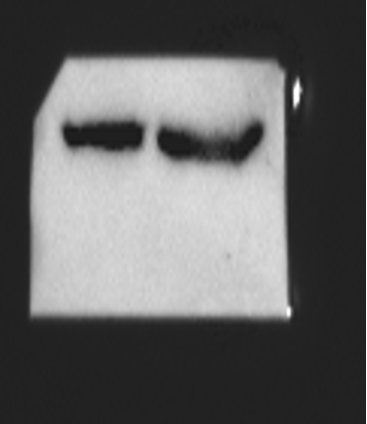

Supplement: Supplementary file 1 [file insects-16-00994-s001.zip › Figure S6/Figure 3E/1/Midgut-actin.tif]

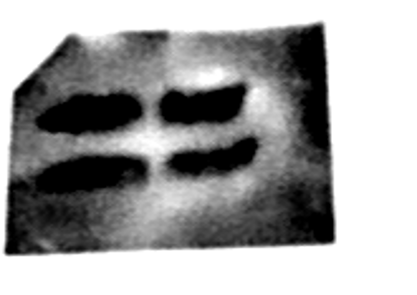

Supplement: Supplementary file 1 [file insects-16-00994-s001.zip › Figure S6/Figure 3E/1/Midgut-atg8.tif]

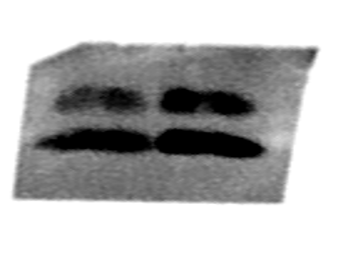

Supplement: Supplementary file 1 [file insects-16-00994-s001.zip › Figure S6/Figure 3E/1/Ovary-atg8.tif]

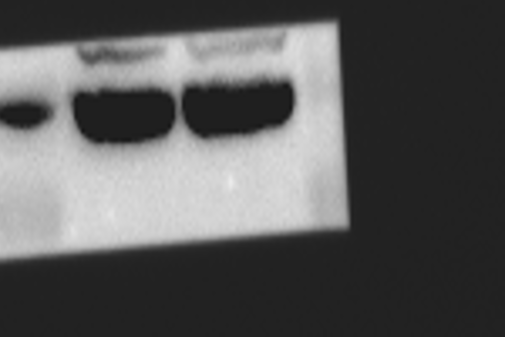

Supplement: Supplementary file 1 [file insects-16-00994-s001.zip › Figure S6/Figure 3E/1/Thorax-actin.tif]

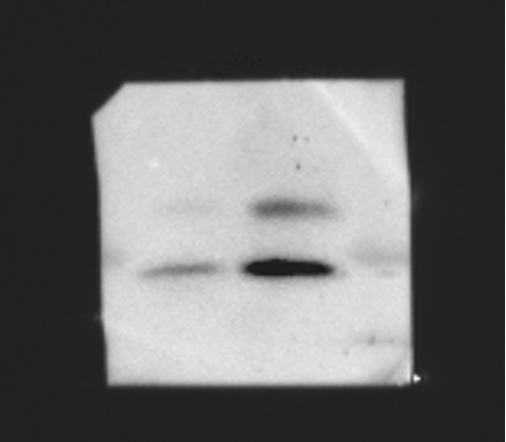

Supplement: Supplementary file 1 [file insects-16-00994-s001.zip › Figure S6/Figure 3E/1/Thorax-atg8.tif]

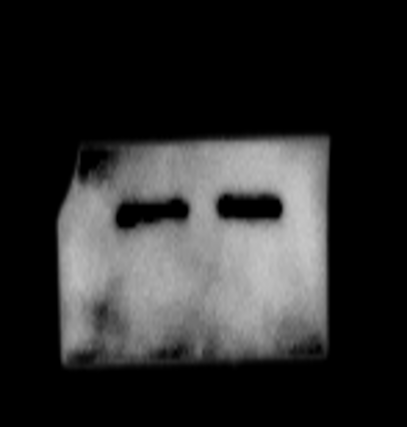

Supplement: Supplementary file 1 [file insects-16-00994-s001.zip › Figure S6/Figure 3E/2/Fatbody-actin.tif]

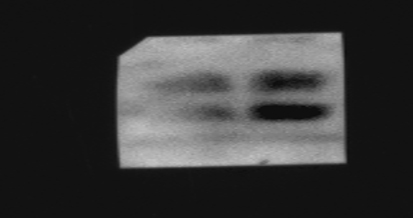

Supplement: Supplementary file 1 [file insects-16-00994-s001.zip › Figure S6/Figure 3E/2/Fatbody-atg8.tif]

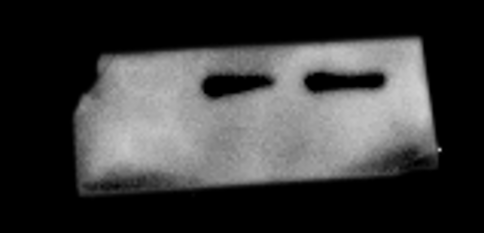

Supplement: Supplementary file 1 [file insects-16-00994-s001.zip › Figure S6/Figure 3E/2/Head-actin.tif]

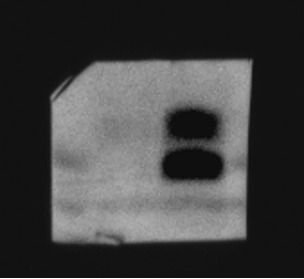

Supplement: Supplementary file 1 [file insects-16-00994-s001.zip › Figure S6/Figure 3E/2/Head-atg8.tif]

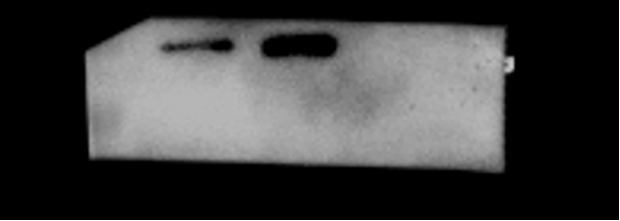

Supplement: Supplementary file 1 [file insects-16-00994-s001.zip › Figure S6/Figure 3E/2/Midgut-actin -.tif]

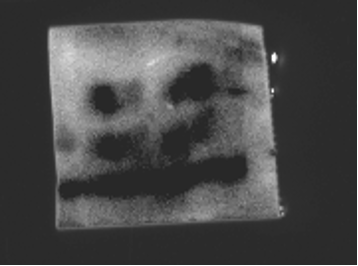

Supplement: Supplementary file 1 [file insects-16-00994-s001.zip › Figure S6/Figure 3E/2/Midgut-atg8.tif]

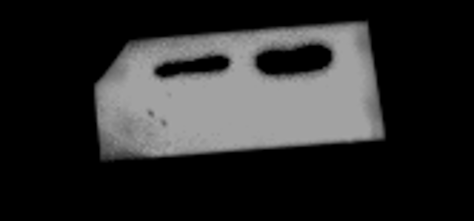

Supplement: Supplementary file 1 [file insects-16-00994-s001.zip › Figure S6/Figure 3E/2/Ovary-actin.tif]

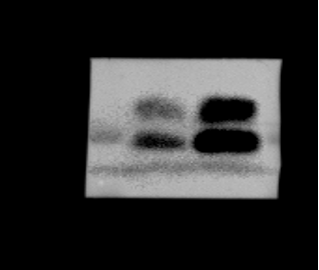

Supplement: Supplementary file 1 [file insects-16-00994-s001.zip › Figure S6/Figure 3E/2/Ovary-atg8.tif]

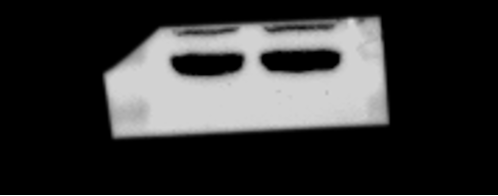

Supplement: Supplementary file 1 [file insects-16-00994-s001.zip › Figure S6/Figure 3E/2/Thorax-actin.tif]

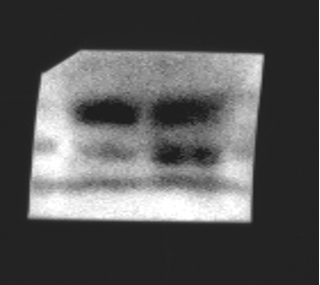

Supplement: Supplementary file 1 [file insects-16-00994-s001.zip › Figure S6/Figure 3E/2/Thorax-atg8.tif]

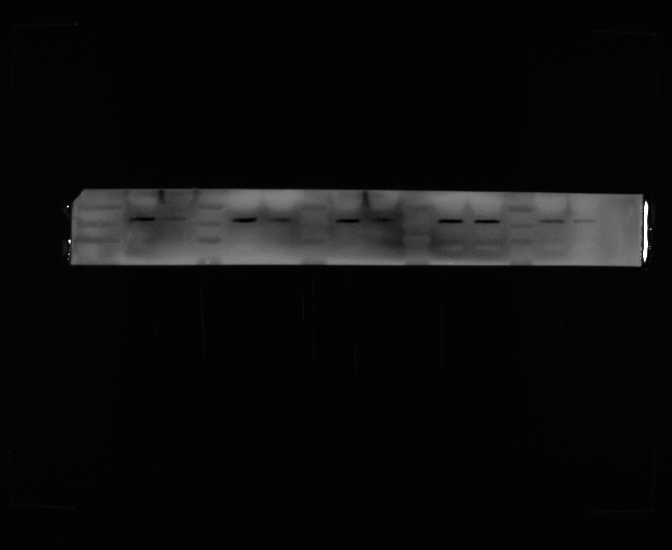

Supplement: Supplementary file 1 [file insects-16-00994-s001.zip › Figure S6/Figure 3E/3/ACTIN-3_8bit.tif]

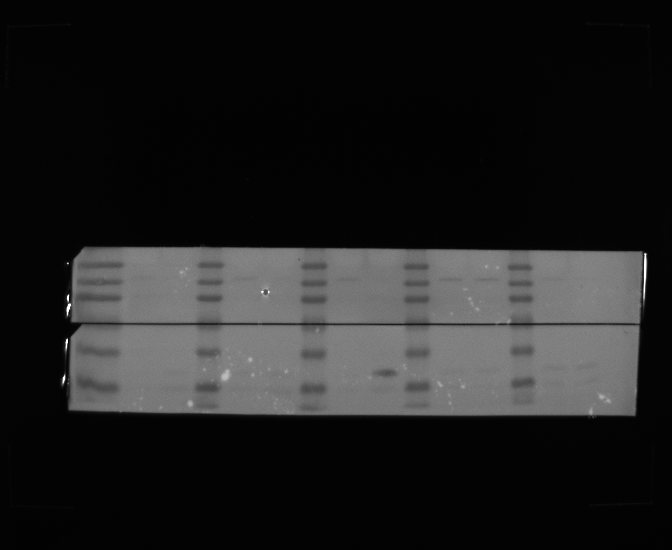

Supplement: Supplementary file 1 [file insects-16-00994-s001.zip › Figure S6/Figure 3E/3/ALL-3.tif]

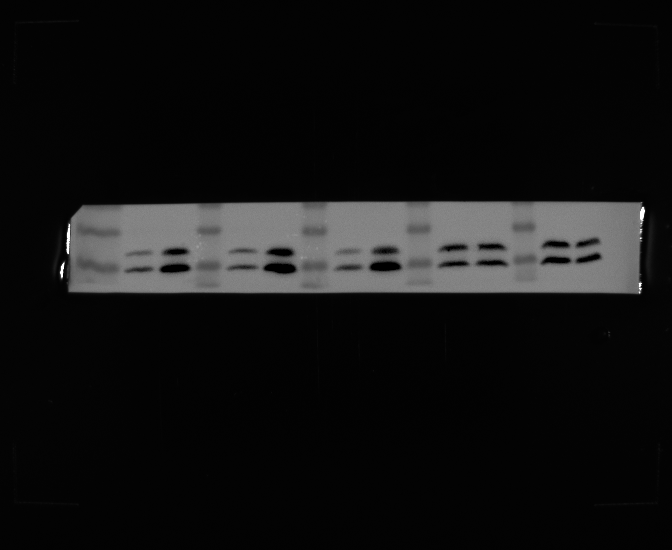

Supplement: Supplementary file 1 [file insects-16-00994-s001.zip › Figure S6/Figure 3E/3/ATG8-3.tif]

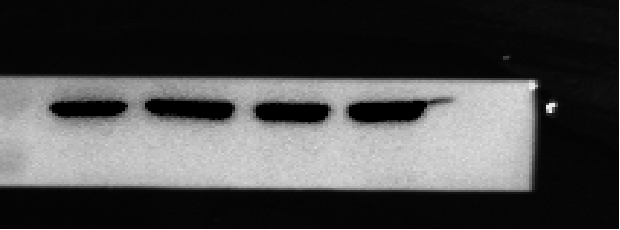

Supplement: Supplementary file 1 [file insects-16-00994-s001.zip › Figure S7/Figure 4A/1/HBSS+bafa actin .tif]

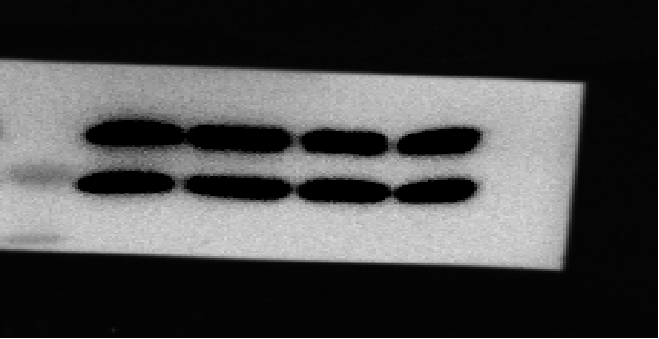

Supplement: Supplementary file 1 [file insects-16-00994-s001.zip › Figure S7/Figure 4A/1/HBSS+BafA-atg8 .tif]

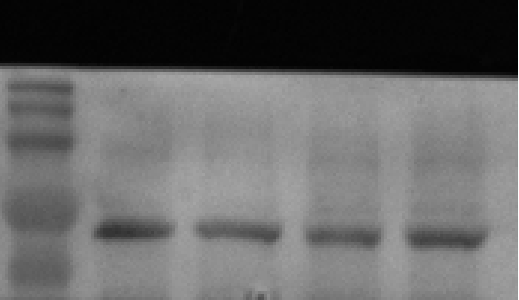

Supplement: Supplementary file 1 [file insects-16-00994-s001.zip › Figure S7/Figure 4A/1/HBSS+bafA-SQSTM1-.tif]

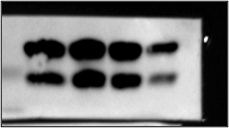

Supplement: Supplementary file 1 [file insects-16-00994-s001.zip › Figure S7/Figure 4A/1/HBSS+MG132---actin+ATG8.tif]

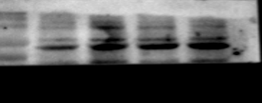

Supplement: Supplementary file 1 [file insects-16-00994-s001.zip › Figure S7/Figure 4A/1/HBSS+MG132---SQSTM1-1.tif]

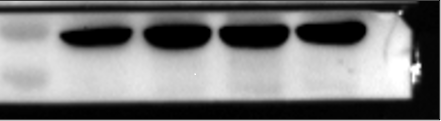

Supplement: Supplementary file 1 [file insects-16-00994-s001.zip › Figure S7/Figure 4A/1/HBSS+MG132-actin.png]

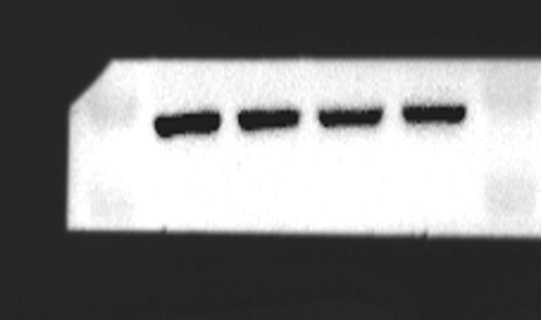

Supplement: Supplementary file 1 [file insects-16-00994-s001.zip › Figure S7/Figure 4A/1/HBSS-actin.tif]

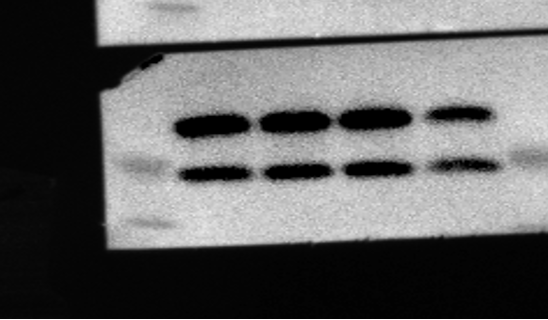

Supplement: Supplementary file 1 [file insects-16-00994-s001.zip › Figure S7/Figure 4A/1/HBSS-atg8.tif]

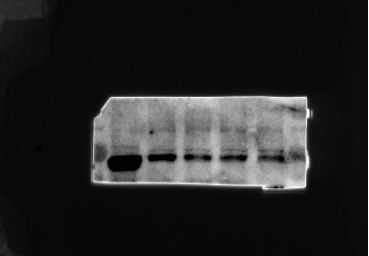

Supplement: Supplementary file 1 [file insects-16-00994-s001.zip › Figure S7/Figure 4A/1/HBSS-SQSTM1.tif]

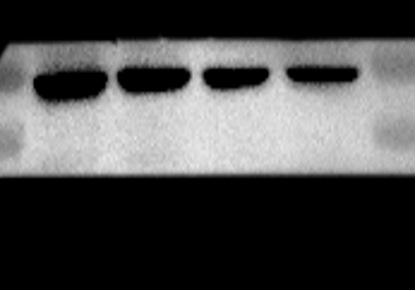

Supplement: Supplementary file 1 [file insects-16-00994-s001.zip › Figure S7/Figure 4A/1/mock-actin.tif]

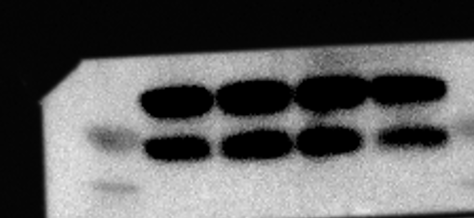

Supplement: Supplementary file 1 [file insects-16-00994-s001.zip › Figure S7/Figure 4A/1/mock-atg.tif]

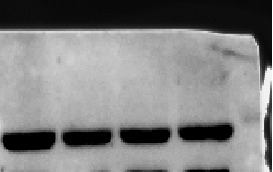

Supplement: Supplementary file 1 [file insects-16-00994-s001.zip › Figure S7/Figure 4A/1/mock-SQSTM1.tif]

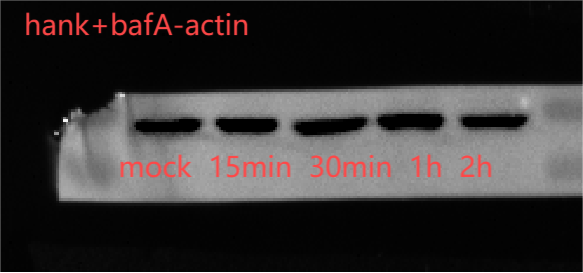

Supplement: Supplementary file 1 [file insects-16-00994-s001.zip › Figure S7/Figure 4A/2/HBSS+bafA actin.png]

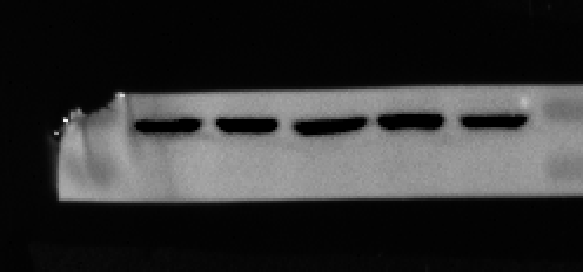

Supplement: Supplementary file 1 [file insects-16-00994-s001.zip › Figure S7/Figure 4A/2/HBSS+bafA actin.tif]

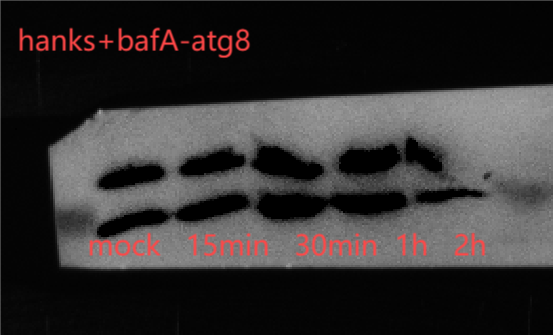

Supplement: Supplementary file 1 [file insects-16-00994-s001.zip › Figure S7/Figure 4A/2/HBSS+bafa-atg8.png]

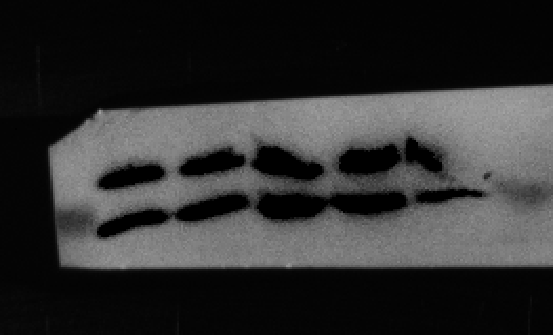

Supplement: Supplementary file 1 [file insects-16-00994-s001.zip › Figure S7/Figure 4A/2/HBSS+bafa-atg8.tif]

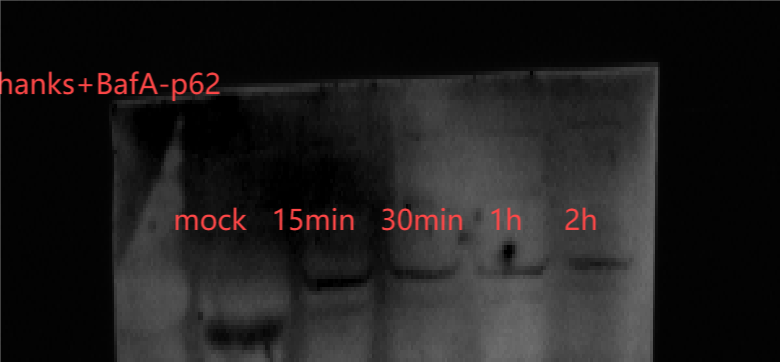

Supplement: Supplementary file 1 [file insects-16-00994-s001.zip › Figure S7/Figure 4A/2/HBSS+bafA-sqstm1.png]

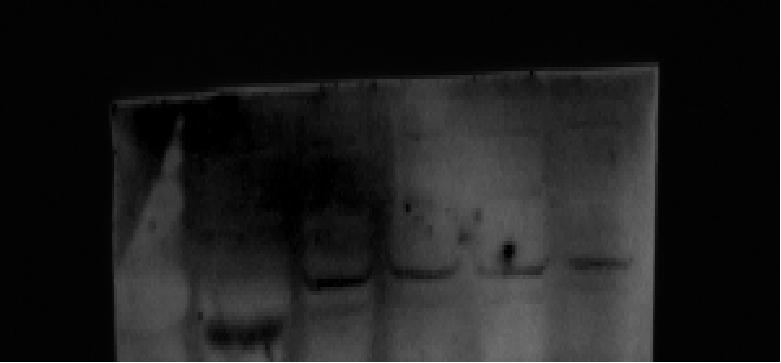

Supplement: Supplementary file 1 [file insects-16-00994-s001.zip › Figure S7/Figure 4A/2/HBSS+bafA-sqstm1.tif]

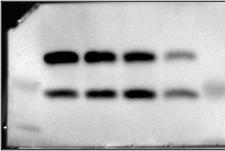

Supplement: Supplementary file 1 [file insects-16-00994-s001.zip › Figure S7/Figure 4A/2/HBSS+MG132---ATG8(1).tif]

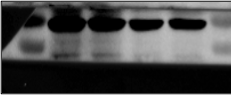

Supplement: Supplementary file 1 [file insects-16-00994-s001.zip › Figure S7/Figure 4A/2/HBSS+MG132-actin(1).tif]

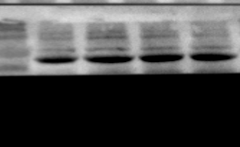

Supplement: Supplementary file 1 [file insects-16-00994-s001.zip › Figure S7/Figure 4A/2/HBSS+MG132-SQSTM1.tif]

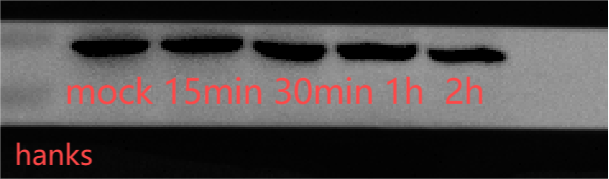

Supplement: Supplementary file 1 [file insects-16-00994-s001.zip › Figure S7/Figure 4A/2/HBSS-actin.png]

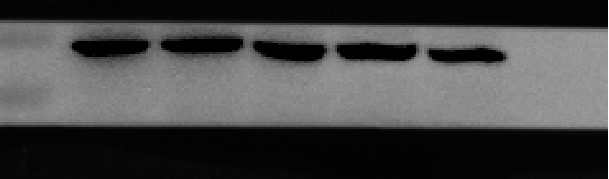

Supplement: Supplementary file 1 [file insects-16-00994-s001.zip › Figure S7/Figure 4A/2/HBSS-actin.tif]

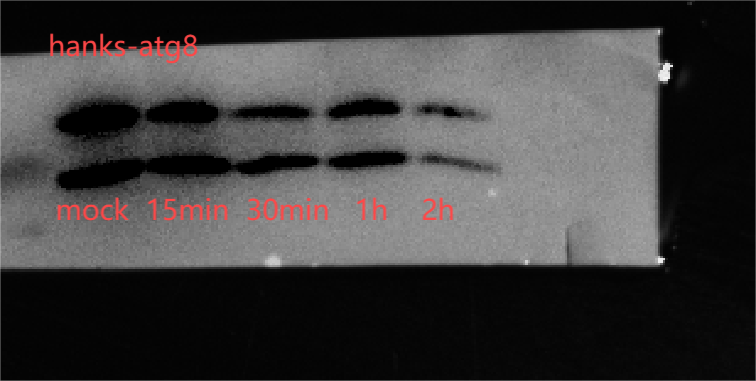

Supplement: Supplementary file 1 [file insects-16-00994-s001.zip › Figure S7/Figure 4A/2/HBSS-atg8.png]

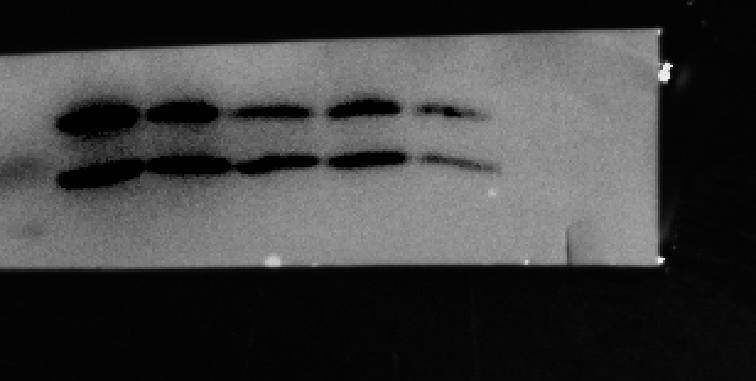

Supplement: Supplementary file 1 [file insects-16-00994-s001.zip › Figure S7/Figure 4A/2/HBSS-atg8.tif]

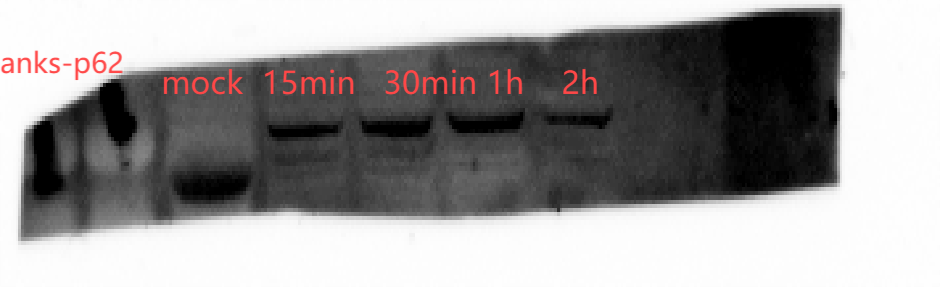

Supplement: Supplementary file 1 [file insects-16-00994-s001.zip › Figure S7/Figure 4A/2/HBSS-SQSTM1.png]

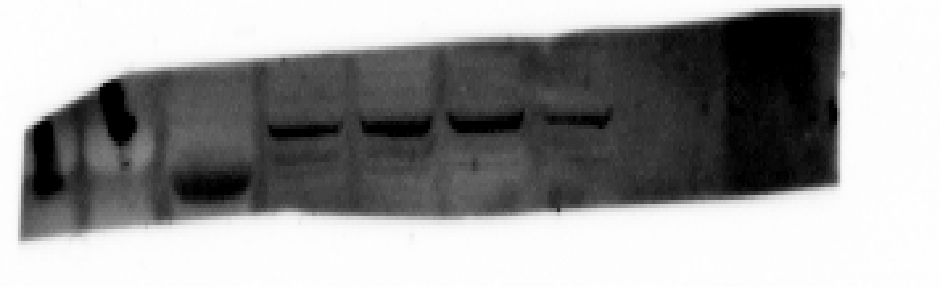

Supplement: Supplementary file 1 [file insects-16-00994-s001.zip › Figure S7/Figure 4A/2/HBSS-SQSTM1.tif]

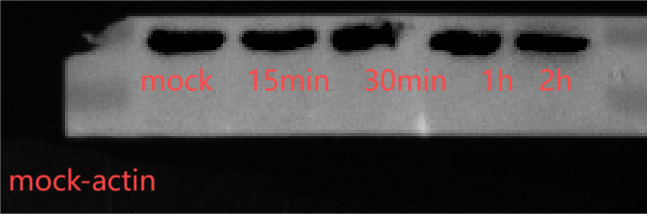

Supplement: Supplementary file 1 [file insects-16-00994-s001.zip › Figure S7/Figure 4A/2/mock-actin.png]

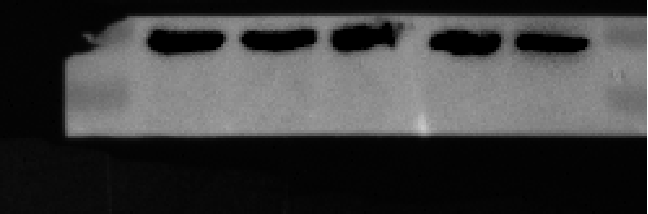

Supplement: Supplementary file 1 [file insects-16-00994-s001.zip › Figure S7/Figure 4A/2/mock-actin.tif]

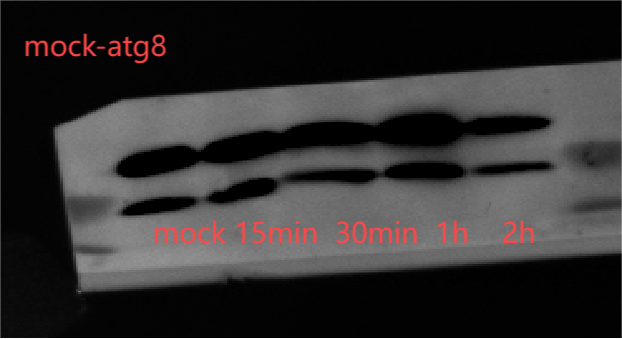

Supplement: Supplementary file 1 [file insects-16-00994-s001.zip › Figure S7/Figure 4A/2/mock-atg8.png]

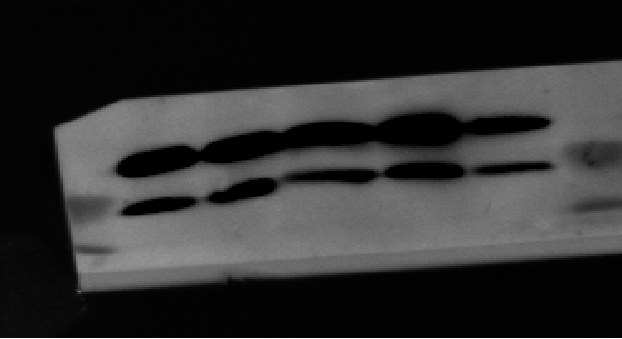

Supplement: Supplementary file 1 [file insects-16-00994-s001.zip › Figure S7/Figure 4A/2/mock-atg8.tif]

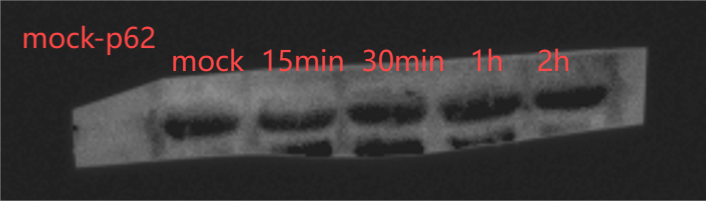

Supplement: Supplementary file 1 [file insects-16-00994-s001.zip › Figure S7/Figure 4A/2/mock-SQSTM1.png]

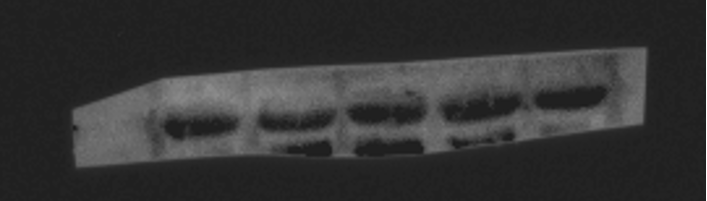

Supplement: Supplementary file 1 [file insects-16-00994-s001.zip › Figure S7/Figure 4A/2/mock-SQSTM1.tif]

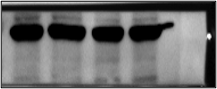

Supplement: Supplementary file 1 [file insects-16-00994-s001.zip › Figure S7/Figure 4A/3/actin(3).tif]

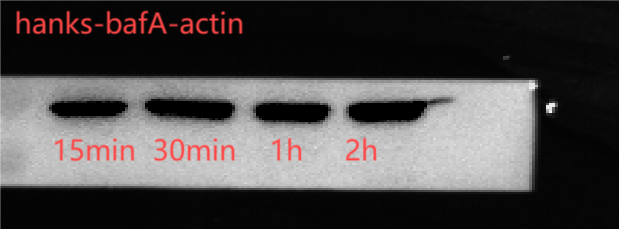

Supplement: Supplementary file 1 [file insects-16-00994-s001.zip › Figure S7/Figure 4A/3/HBSS+bafa actin.png]

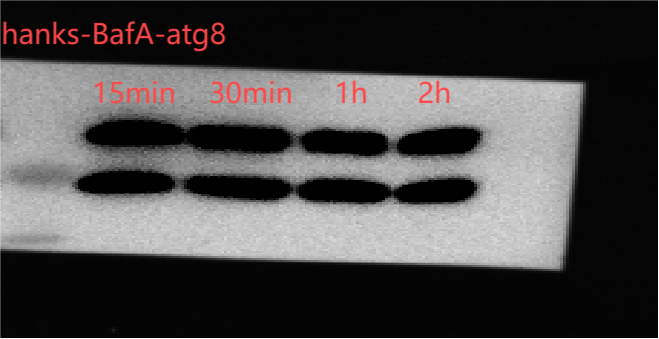

Supplement: Supplementary file 1 [file insects-16-00994-s001.zip › Figure S7/Figure 4A/3/HBSS+BafA-atg8.png]

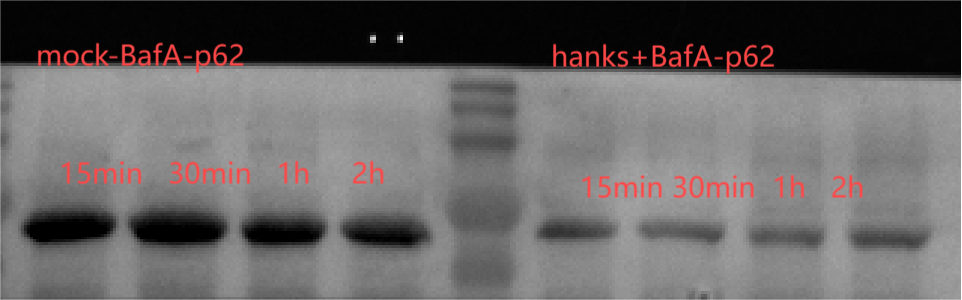

Supplement: Supplementary file 1 [file insects-16-00994-s001.zip › Figure S7/Figure 4A/3/HBSS+bafA-SQSTM1.png]

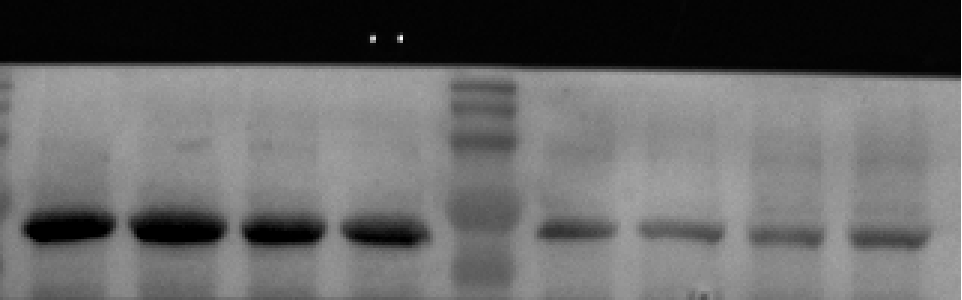

Supplement: Supplementary file 1 [file insects-16-00994-s001.zip › Figure S7/Figure 4A/3/HBSS+bafA-SQSTM1.tif]
